# Diastereoselective Cyclopropanation with Secondary Diazoacetamides to Access *endo*-Azabicyclo[3.1.0]hexane-6-carboxamides

**DOI:** 10.1021/acs.orglett.6c00392

**Published:** 2026-02-23

**Authors:** Terrence-Thang H. Nguyen, Takeru Saito, Warren Chang, Antonio Navarro, Huw M. L. Davies

**Affiliations:** ∇ Department of Chemistry, 1371 Emory University, Atlanta, Georgia 30322, United States; ‡ Lilly Research Laboratories, Eli Lilly and Company, Indianapolis, Indiana 46285, United States

## Abstract

A dirhodium­(II) tetracarboxylate-catalyzed reaction of secondary diazoacetamides with *N*-Boc-2,5-dihydro-1*H*-pyrrole results in a highly diastereoselective cyclopropanation for the synthesis of *endo*-azabicyclo­[3.1.0]­hexane-6-carboxamides. These reaction conditions work well for secondary diazoacetamides but are not compatible with their tertiary amide counterparts. A base-mediated equilibration of the *endo*-isomer allows access to the *exo*-azabicyclo­[3.1.0]­hexane-6-carboxamides. The utility of this cyclopropanation chemistry was illustrated by its application to the synthesis of the drug candidate, Mazisotine.

## Graphical abstract

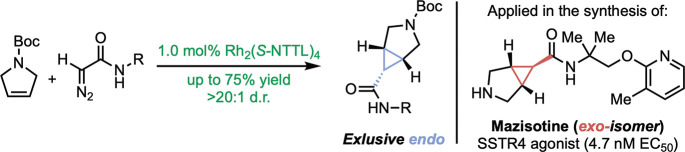

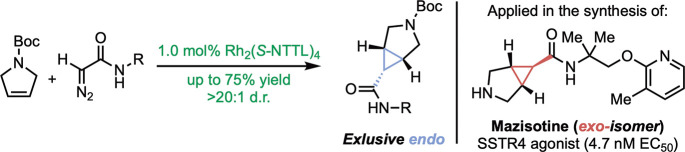

Bicyclic scaffolds have become increasingly popular in drug design due to their unique three-dimensional structure and conformational rigidity. Consequently, these bicyclic structures have been explored as bioisosteres of common, planar drug motifs. For example, 1,3-disubstituted bicyclo[1.1.1]­pentanes or 1,4-disubstituted bicyclo[2.2.2]­octanes have been frequently employed as 1,4-disubstituted benzene replacements.^1^ Within this area, 3-azabicyclo[3.1.0]­hexane-6-carboxylates have garnered attention as bioisosteres of piperidines or other aza-containing scaffolds.^2^ The substitution at the 6-position of this scaffold results in a meso compound that can adopt either the *exo* or *endo* form, each with distinct spatial orientations. As illustrated in representative examples in Figure 1, these substructures are found in a number of pharmaceutical compounds (**1**–**4**), exhibiting excellent bioactivity.^2c,3^

**Figure 1:**
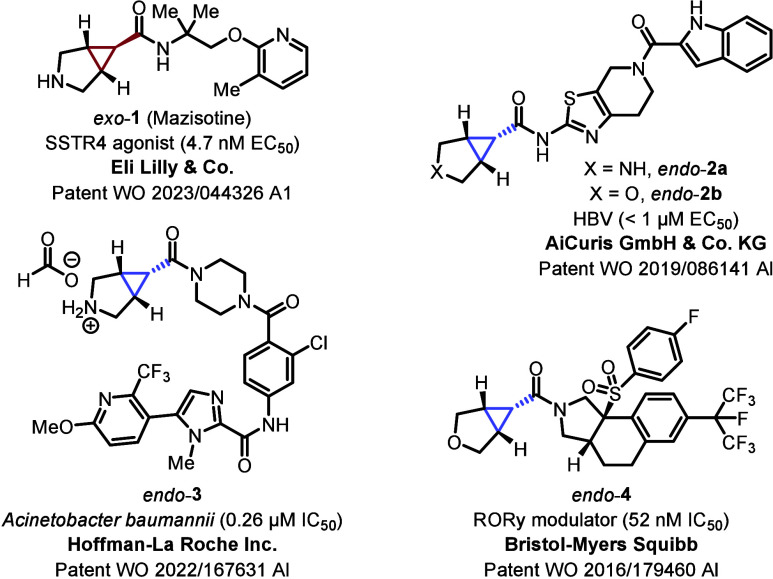
Relevant bioactive compounds with bicyclo-[3.1.0]­hexane-6-carboxamides (**1–4**)^2c,3^

Due to the biological significance of this substructure, considerable interest has been shown in developing a practical entry into this scaffold. The majority of the reported methods to generate 3-azabicyclo[3.1.0]­hexane-6-carboxylates have involved multistep sequences that do not start with a pyrrolidine derivative.^4^ A particularly direct approach, however, would be the cyclopropanation of dihydropyrrole with a diazocarbonyl derivative.^5^ Early examples of the cyclopropanation of *N*-Boc-2,5-dihydro-1*H*-pyrrole (**5**) with ethyl diazoacetate were not ideal because they required relatively high catalyst loadings (1 mol % or above).^2d,6^ Recently, we reported an improved procedure in which the reaction could be conducted under low catalyst loading (0.005 mol %)­(Figure 2A).^7^ This reaction affords a mixture of the *endo*- and *exo*-diastereomers **6**, the ratio of which could be controlled to some extent by judicious choice of the catalyst (5:1 to 1:1, *endo*/*exo*). The product **6** can also be either selectively hydrolyzed or isomerized to exclusively form either *endo*-**7** or *exo*-**7** without chromatographic purification.^7^

**Figure 2:**
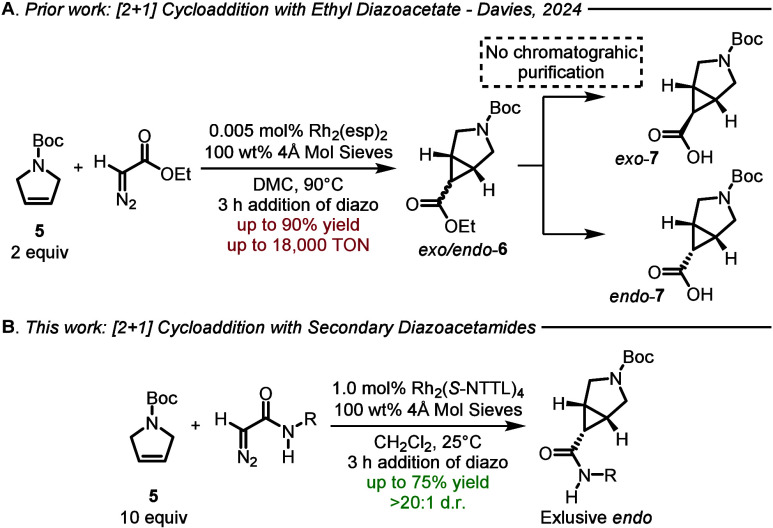
Previous and current work.

Even though the previous cyclopropanation approach offers an attractive entry to the 3-azabicyclo[3.1.0]­hexane scaffold required for the synthesis of the pharmaceutical targets **1–4**, the synthesis of these compounds would be more convergent if a diazoacetamide was used as the carbene precursor instead of a diazoacetate. Furthermore, it would be especially desirable for the reaction to selectively form the less stable *endo* isomer. Then, either diastereomeric series of the drug targets would be accessible as it should be possible to isomerize the *endo* isomer to the *exo* isomer. Even though diazoacetates have been the most widely used carbene souces for intermolecular cyclopropanation, early studies by Doyle showed that diazoamides can cyclopropanate monosubstituted alkenes with enhanced *endo* selectvity,^8^ which serves as good precedence for the proposed transformation. This manuscript describes a systematic study to develop a stereoselective cyclopropanation of dihydropyrrole **5** with elaborate diazoamides of pharmaceutical interest, followed by its application to the synthesis of the drug candidate Mazisotine (**1**).

We began our studies with a catalyst screen (Figure 3) to determine which catalyst would give the highest diastereoselectivity, using diazoacetamide **8** as the carbene precursor, as this would rapidly lead to the synthesis of Mazisotine (**1**).^2c^ A list of catalysts utilized with structures are provided in the SI, page S5–6. ^1^H NMR analysis of the reaction mixture prior to column chromatography was used to determine diastereoselectivity and these data are included in the SI, Figure S2 (page S7). Achiral catalysts Rh_2_(OAc)_4_ and Rh_2_(esp)_2_ (entries 1 and 2) produced **9** in low *endo*:*exo* ratios of 2.7:1 and 0.9:1, respectively. Many of the classic chiral catalysts also failed to achieve effective control of the diastereoselectivity (entries 3–6). Rh_2_(*S*-PTAD)_4_ and Rh_2_(*S*-NTTL)_4_, however, two flexible *C*_4_ symmetric shallow bowl-shaped catalysts showed high diastereoselectivity, 14.4:1 and >40:1 *endo*:*exo*, respectively (entries 7 and 8).

**Figure 3:**
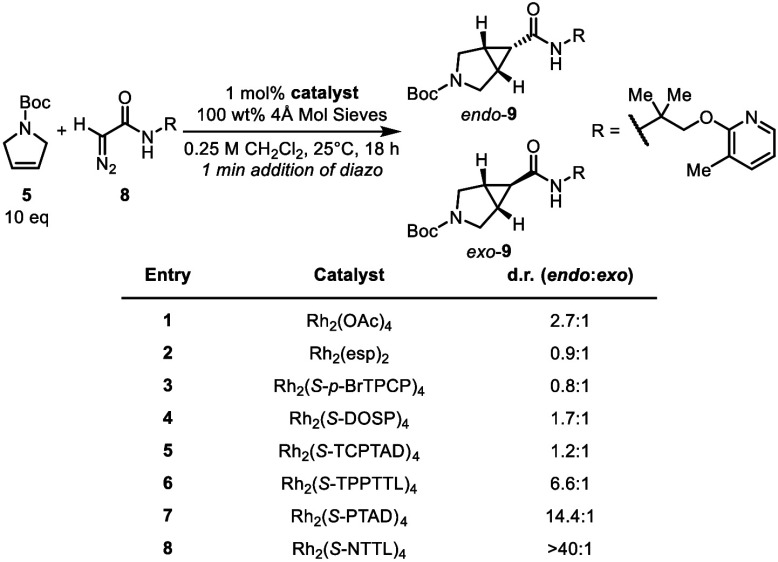
Catalyst screen of [2 + 1] cycloaddition with diazo compound **8**.

Using Rh_2_(*S*-NTTL)_4_, we next focused on optimizing the reaction conditions (Figure 4). In order to avoid carbene side reactions and aid reactivity, we found it was best to carry out the reaction with an excess of the dihydropyrrole **5** (entries 1–3). When 10 equiv of **5** was used, *endo*-**9** was isolated in 58% yield and the excess unreacted dihydropyrrole **5** could be cleanly recovered by a short-path distillation prior to chromatographic purification.

**Figure 4:**
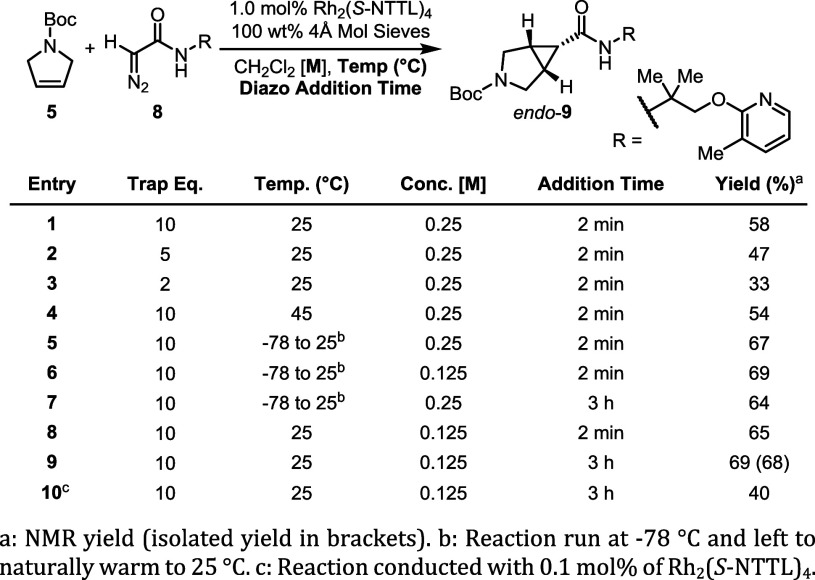
Reaction optimization of [2 + 1] cycloaddition.

While heating the reaction did not increase the yield of the desired product (entry 4), starting the reaction at −78 °C and allowing it to gradually warm to ambient temperature over the course of the reaction increased the yield to 67% yield (entry 5). Increasing the reaction concentration 2-fold showed slightly improved results (entry 6). A 3 h slow addition of **8** at −78 °C (entry 7) and a fast addition of **8** at 25 °C (entry 8) only decreased the yield by a small amount. Lastly, decreasing the catalyst loading 10-fold to 0.1 mol % lowered the yield to 40%. From this study, we obtained two comparable reaction conditions for running this reaction. The first method is a fast addition of the diazo compound at −78 °C and allowing the reaction to warm to 25 °C overnight (entry 6). The second is to introduce the diazo compound over 3 h but run the reaction at 25 °C (entry 9). We found that the former reaction conditions were not applicable to smaller alkyl amide diazo compounds because with these substrates, significant carbene dimerization occurred. Thus, we decided to explore the scope of the reaction with the second set of optimized conditions.

With optimal reaction conditions in hand, we next explored whether tertiary diazoacetamides generated from their corresponding secondary amines could also react in the same fashion. It is well established that with bulky tertiary amides, intramolecular C–H functionalization is a favorable reaction.^9^ Doyle discovered that, in the Rh_2_(OAc)_4_-catayzed cyclopropanation reactions of various alkenes, the yields increase as the alkenes become more electron-rich.^10^ In addition, as the alkyl substituents on the tertiary amide became larger, the intramolecular reactivity outcompeted the desired intermolecular cyclopropanation reaction. We decided to model this reaction with N,N-dimethylamide **10**, which was hypothesized to not strongly influence the system toward intramolecular C–H insertion (Scheme 1). However, the attempted reaction with an electron-deficient alkene such as **5** resulted in full consumption of the diazo compound **10** but no productive cyclopropanation was observed and the ^1^H NMR of the crude reaction showed broad signals, lacking any definitive peaks (see SI Figure S3).

**Scheme 1:**
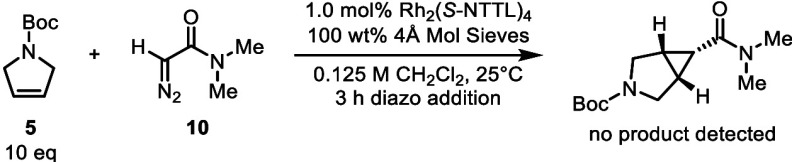
Comparison of Secondary and Tertiary Amide Systems

Having established that secondary diazoacetamides are favored for the cyclopropanation reaction, we conducted a study to determine the scope of the reaction (Figure 5). We first examined simple amides, gradually increasing the steric environment around the carbene center from methyl (**11**), ethyl (**12**), *iso*-propyl (**13**), then to *tert*-butylamide (**14**). These reactions all proceeded with good yield (51 – 72%) and excellent diastereoselectivity (>20:1 *endo*:*exo*). In addition, we observed minimal to no intramolecular reactivity with the more sterically bulky amides. With the *N*-allylamide derived diazoacetamide, we saw good conversion to the intermolecular cyclopropanation product **15** over the potential intramolecular C–H insertion or cycloaddition reactions. We also observed good conversion with the aniline and methyl glycine analogs to form cyclopropanes **16** and **17**, respectively, both of which exclusively formed the *endo* diastereomers. The last set of compounds explored (**18** – **19**) were selected as drug-like scaffolds related to **1**. These all showed favorable reactivity and diastereoselectivity profiles.

**Figure 5:**
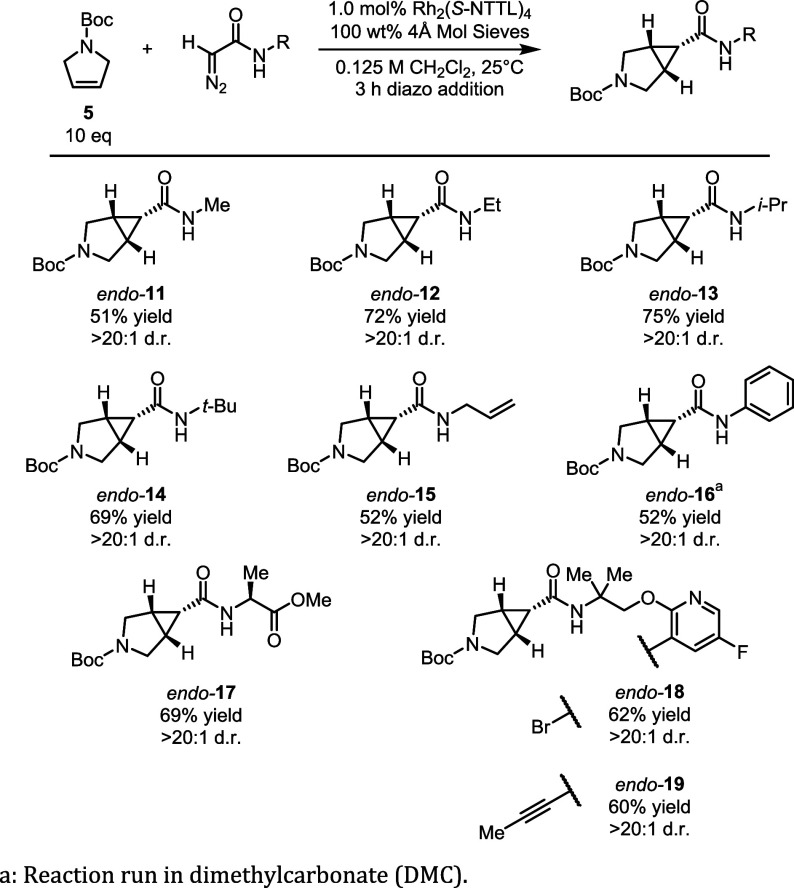
Exploration of amide scope.

In addition to reactions with **5**, we also explored other 5-membered 1,2-disubstituted cyclic alkene traps, shown in Figure 6. Dihydrofuran, dihydrothiophene dioxide, and cyclopentene were all effective substrates, resulting in the formation of **21**-**23**, exclusively as the *endo* isomers. The reaction with dihydrofuran is of particular interest because it could be used for the synthesis of pharmaceutical scaffolds related to **2b** and **4**.

**Figure 6:**
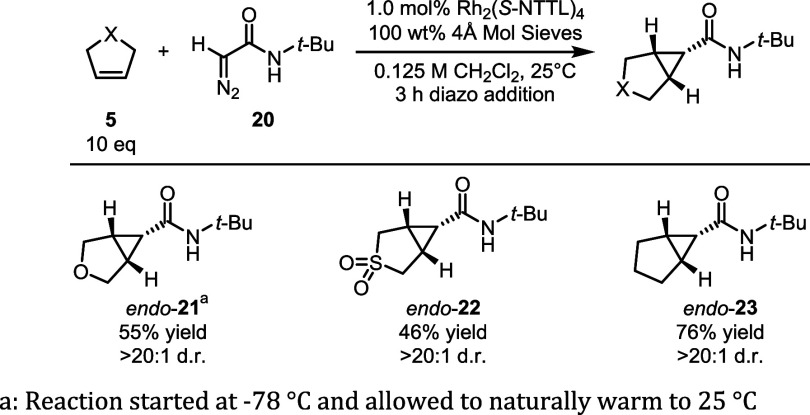
Expansion of trap scope.

Lastly, the cyclopropanation reaction was applied to a formal synthesis to Mazisotine (**1**), a SSTR4 agonist developed by Lilly,^2c^ as illustrated in Scheme 2. Diazo compound **24** can be obtained in two steps adapting the literature precedent (see SI for details),^11^ which can then be elaborated to diazo **8** via amidation with amine **25**. On larger scale, we elected to run the key cyclopropanation using 0.1 mol % of Rh_2_(*S*-NTTL)_4_ at 25 °C, which afforded *endo*-**9** in 50% yield with a > 20:1 *endo*:*exo* ratio. In order to complete the formal synthesis of Mazisotine, it would be necessary to epimerize *endo*-**9** to *exo*-**9**. After considerable experimentation, it was found that the base sodium *tert*-pentoxide was suitable for the epimerization of *endo*-**9** to generate *exo*-**9** in 75% yield with a > 20:1 *exo*:*endo* ratio,^12^ completing the formal synthesis. The final step is a deprotection of the *N*-Boc group to afford Mazisotine (**1**), which is a known literature procedure (not illustrated here).^2c^

**Scheme 2:**
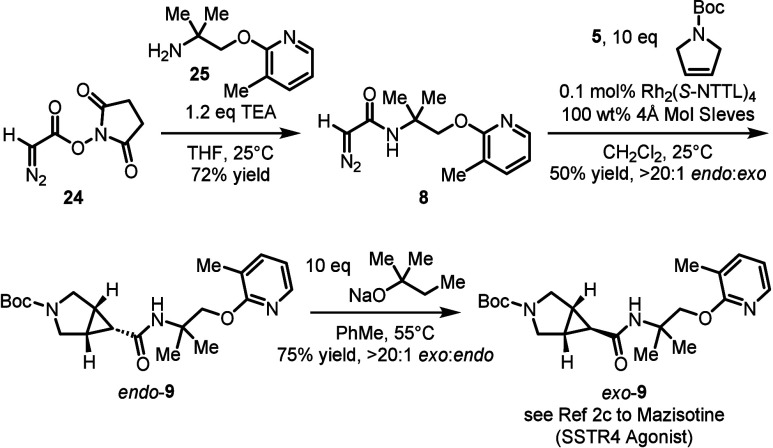
Application toward the Synthesis of Mazisotine

In conclusion, these studies demonstrate that secondary diazoacetamides are suitable carbene precursors for the cyclopropanation of *N*-Boc-2,5-dihydropyrrole, greatly shortening the synthesis of Mazisotine and other related drug-like scaffolds. The cyclopropanation with secondary diazoacetamides can be conducted with a range of different derivatives but the reaction fails with tertiary diazoacetamides. These studies further underscore the versatility of the cyclopropanation of dihydropyrrole for the synthesis of pharmaceutically significant *endo*-azabicyclo­[3.1.0]­hexane-6-carboxamides.

## Supplementary Material



## Data Availability

The data underlying this study are available in the published article and its online Supporting Information.

## Notes

Complete experimental procedures, materials and compound characterizations. (PDF)
