# Supplementary material for: Diastereoselective Cyclopropanation with Secondary Diazoacetamides to Access endo-Azabicyclo[3.1.0]hexane-6-carboxamides
Source: Org Lett. 2026 Feb 23;28(9):3063–7. doi: 10.1021/acs.orglett.6c00392 (PMC12973298; doi:10.1021/acs.orglett.6c00392)

Diastereoselective Cyclopropanation with Secondary Diazoacetamides to Access *Endo*-Azabicyclo[3.1.0]hexane-6-carboxamides

Terrence-Thang H. Nguyen[†1], Takeru Saito[†1], Warren Chang[1], Antonio Navarro[2], Huw M. L. Davies[1]*

[1]Department of Chemistry, Emory University, Atlanta, Georgia 30322, United States
[2]Lilly Research Laboratories, Eli Lilly and Company, Indianapolis, Indiana 46285, United States
[†]Equal contribution from authors

Corresponding author email: hmdavie@emory.edu

Supporting Information

Complete experimental procedures, materials, and compound characterizations

Table of Contents

1. General Considerations S2
2. Reagents Acquired through Vendors S4
3. Figures S5
4. References S9
5. Procedures and Compound Characterizations S11
6. Spectra S43

1. General Considerations

Warning: This project involves the use of diazo compounds. Diazo compounds are known to have thermal stability issues and are explosive hazards. Work with diazo compounds should be performed in a well-ventilated hood, require the use of PPE, and careful handling of the reagents.[1] Any excess diazo or azide reagent was quenched by treating it with O_3 using an ozone generator.

Reaction Setup and Purification

All reactions were performed under a positive pressure of inert atmosphere (nitrogen or argon) in flame-dried glassware fitted with rubber septa or septa-lined plastic vial caps, unless specified otherwise. Ambient temperature was measured as 25°C. Unless otherwise noted, refluxed reactions were done so on metal heating blocks in round-bottom flasks equipped with Heidolph findensers. -78°C was achieved by acetone and dry ice in a vacuum-sealed dewar. Solvents denoted as 'SPS grade' [dichloromethane (CH_2Cl_2), acetonitrile (MeCN), tetrahydrofuran (THF)] were dried by passing through alumina columns under argon in a Glass Contour Solvent Purification System (SPS). Solvents denoted as 'distilled' were done so from calcium hydride (CaH_2) and stored in a flask under an inert atmosphere over activated 4Å molecular sieves (Mol Sieves), bubbling with argon for 15 min, then letting sit at least 24 h before use. 4Å Mol Sieves were activated by high temperature (> 200°C) under vacuum and stored in an oven until use. The amount of 4Å Mol Sieves denoted as '100 wt%' was calculated as 1 g of 4Å Mol Sieves for 1 mmol of diazo utilized. All reagents were purchased from commercial suppliers (Sigma Aldrich, Oakwood, Ambeed, TCI, Alfa Aesar, CombiBlocks, Acros) and used directly without further purification, unless otherwise noted. Reactions were monitored by thin-layer chromatography (TLC) on silica-coated aluminum sheets, visualizing with 254 nm UV light and staining, as indicated. Automated flash column chromatography was performed on Biotage Isolera flash chromatography systems using Silicycle SiliaFlash P60 silica gel (60 Å pore size, 40-63 μm particle size, 230-400 mesh) and reagent grade solvents.

NMR Spectroscopy and Processing

Raw FID files were processed and analyzed using the MestReNova 15.1 software from Mestrelab Research S. L.. All ^{1}H NMR spectra were recorded at 400 MHz or 600 MHz on Bruker AVIII-400, Bruker NEO-400, or Bruker AVIII-600 spectrometers. ^{13}C NMR spectra were recorded at 101 MHz or 151 MHz, while ^{19}F NMR spectra were recorded at 376 MHz. NMR spectra were obtained using deuterated chloroform ($CDCl_3$), with or without 0.03% TMS with residual solvent serving as internal standard (7.26 ppm for ^{1}H and 77.16 ppm for ^{13}C). ^{1}H NMR chemical shifts are reported in parts per million (ppm). Abbreviations for signal multiplicity are as follows: s = singlet, d = doublet, t = triplet, q = quartet, p = pentet, hept = heptet, m = multiplet, dd = doublet of doublets, br s = broad singlet, app = apparent multiplicity, etc. ^{19}F NMR and ^{13}C NMR chemical shifts are reported in parts per million (ppm) and when appropriate, multiplicity. Coupling constants (J values) were calculated directly from the spectra. All deuterated solvents utilized were purchased from Sigma Aldrich. Deuterated chloroform ($CDCl_3$) was neutralized with a small addition of oven-dried potassium carbonate (K_2CO_3) and dried with activated 4Å Mol Sieves, letting sit overnight before use.

High Resolution Mass Spectrometry (HRMS)
Mass spectra were taken on a Thermo Finnigan LTQ-FTMS spectrometer with positive (+p) or negative (-p) Atmospheric Pressure Chemical Ionization (APCI) or Electron Spray Ionization (ESI)

Abbreviations
CV = Column Volume
d.r. = diastereomeric ratio
Mol Sieves = Molecular Sieves
RBF = Round Bottom Flask
R_f = Retention Factor
SM = Starting Material
SPS = Solvent Purification System
TLC = Thin-Layer Chromatography
UV = Ultraviolet
qNMR = quantitative NMR (using standard procedures from Sigma Aldrich)
https://www.sigmaaldrich.com/deepweb/assets/sigmaaldrich/marketing/global/documents/101/854/qnmr-brochure-rjo.pdf

Chemical Abbreviations
DBU = 1,8-Diazabicyclo[5.4.0]undec-7-ene (Cas No. 6674-22-2)
DCC = N,N'-Dicyclohexylcarbodiimide (Cas No. 538-75-0)
TEA = Triethylamine (Cas No. 121-44-8)

Solvent Abbreviations
DCM/CH_2Cl_2 = Dichloromethane
DMC = Dimethylcarbonate
EtOAc = Ethyl Acetate
Et_2O = Diethyl ether
Hex = Hexanes
MeCN = Acetonitrile
MeOH = Methanol
THF = Tetrahydrofuran

2. Reagents Acquired through Vendors

Reagent	Cas No.	Vendor(s)	Notes
tert-Butyl 2,5-dihydro-1*H*-pyrrole-1-carboxylate (*N*-Boc-2,5-dihyropyrrole or *N*-Boc-3-pyrroline) (**5**)	73286-70-1	Ambeed, TCI, Combi-Blocks	Impurities (mainly *N*-Boc pyrrole) present in samples from all vendors. Reagent should be a white crystalline solid. Repurified before use. See previous study for reagent purification.[2]
N,N'-Dicyclohexylcarbodiimide (DCC)	538-75-0	Sigma Aldrich	Used fresh for coupling reactions to make **24**. Weighed as a solid.
2-oxoacetic acid hydrate; glyoxylic acid monohydrate	298-12-4	Oakwood, Sigma Aldrich, Combi-Blocks	Must be crystalline cream solid. Incomplete reaction observed when liquid form was used.
N-Hydroxysuccinimide	6066-82-6	Oakwood, Sigma Aldrich	Used fresh for coupling reactions to make **24**.
Sodium *tert*-pentoxide, 40 wt% in toluene	14593-46-5	Oakwood	Solution contains crystals. Added to solution by weighing scale.

3. Figures

The following chiral rhodium(II) catalysts were used in this study:

Catalyst	Molecular Weight (g/mol)	Catalyst Information
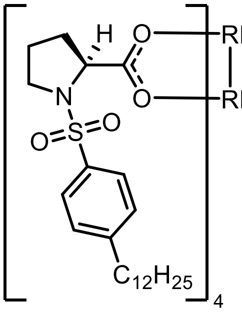	1896.22	Rh_2(*S*-DOSP)$_4$<br><br>**Generation:** Prolinato<br>*J. Am. Chem. Soc.* **1996**, *118*, 6897–6907[3]
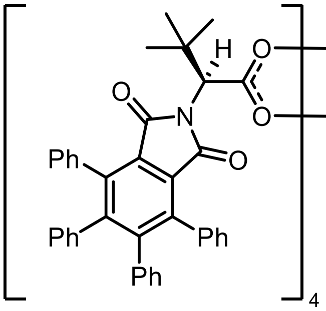	2464.45	Rh_2(*S*-TPPTTL)$_4$<br><br>**Generation:** Phthalimido<br>*Nature*, **2018**, *564*, 395-399[4]
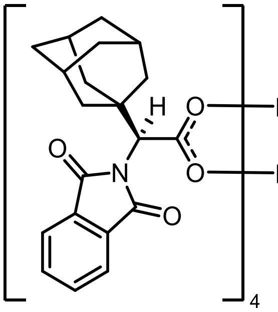	1559.34	Rh_2(*S*-PTAD)$_4$<br><br>**Generation:** Phthalimido<br>*Org. Lett.* **2006**, *8*, 5013–5016[5]
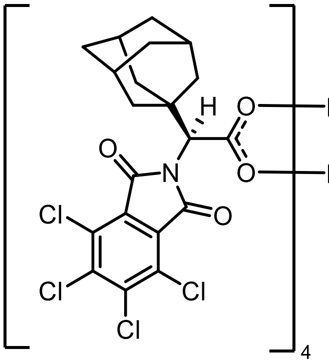	2110.44	Rh_2(*S*-TCPTAD)$_4$<br><br>**Generation:** Phthalimido<br>*Org. Lett.* **2006**, *8*, 5013–5016[5]

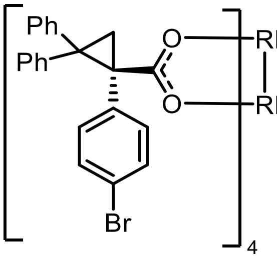	1774.87	Rh_2(*S*-*p*-BrTPCP)$_4$<br><br>**Generation:** TPCP<br>*J. Am. Chem. Soc.* **2014**, *136*, 9792–9796[6]
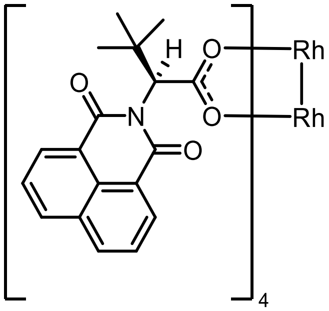	1447.13	Rh_2(*S*-NTTL)$_4$<br><br>**Generation:** Naphthalimido<br>*Chem Eur J*, **2010**, *16*, 3291[7]

Figure S1. Rhodium(II) catalyst structures used in catalyst screen.

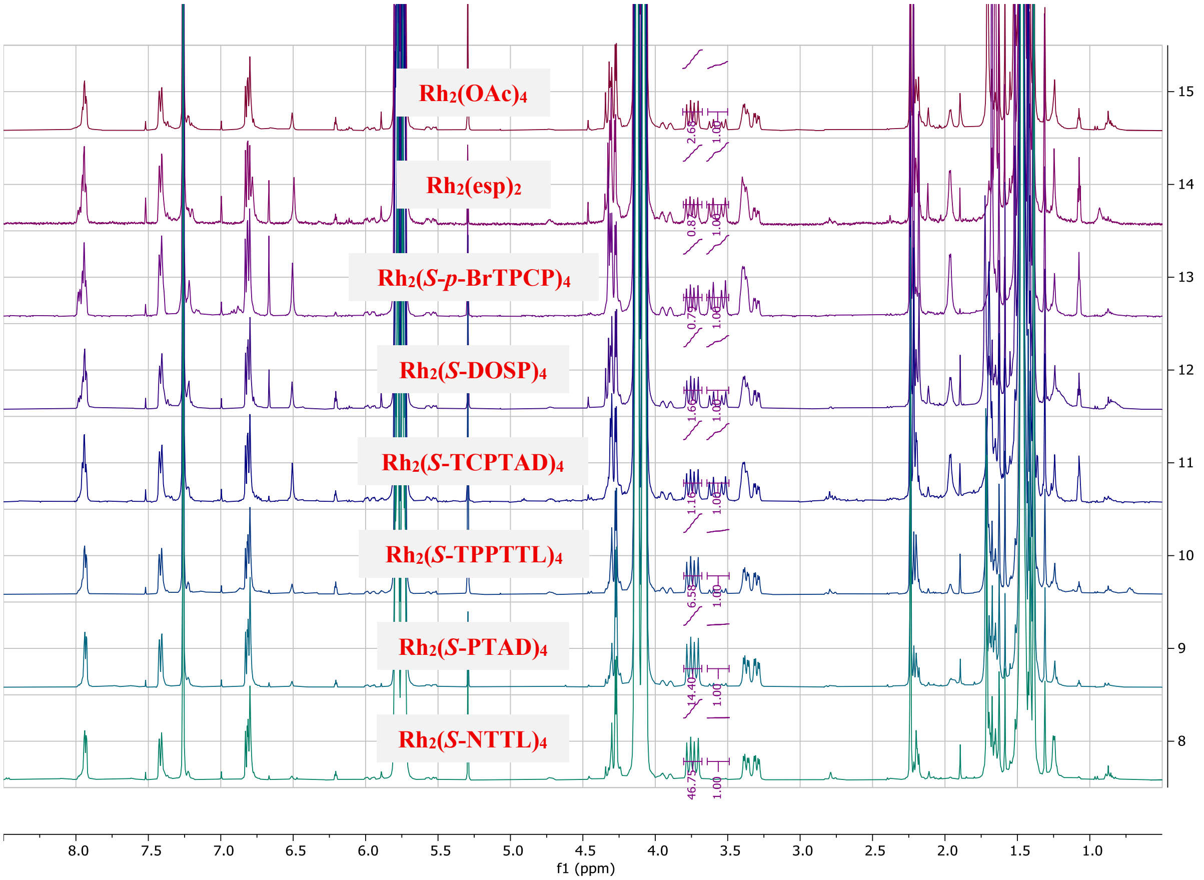

Figure S2. Crude ^{1}H NMR of catalyst screen with diazoacetamide **8** to access **9**.

Signal at 3.74 (dd, J = 20.8, 11.0 Hz) assigned as α to nitrogen proton of *endo* diastereomer
Signal at 3.57 (dd, J = 33.7, 11.2 Hz) assigned as α to nitrogen proton of *exo* diastereomer

Catalysts utilized and respective d.r., shown as endo:exo (from top to bottom):
$Rh_2(OAc)_4$: 2.7:1
$Rh_2(esp)_2$: 0.9:1
Rh_2(*S*-*p*-BrTPCP)$_4$: 0.8:1
Rh_2(*S*-DOSP)$_4$: 1.7:1
Rh_2(*S*-TCPTAD)$_4$: 1.2:1
Rh_2(*S*-TPPTTL)$_4$: 6.6:1
Rh_2(*S*-PTAD)$_4$: 14.4:1
Rh_2(*S*-NTTL)$_4$: >40:1

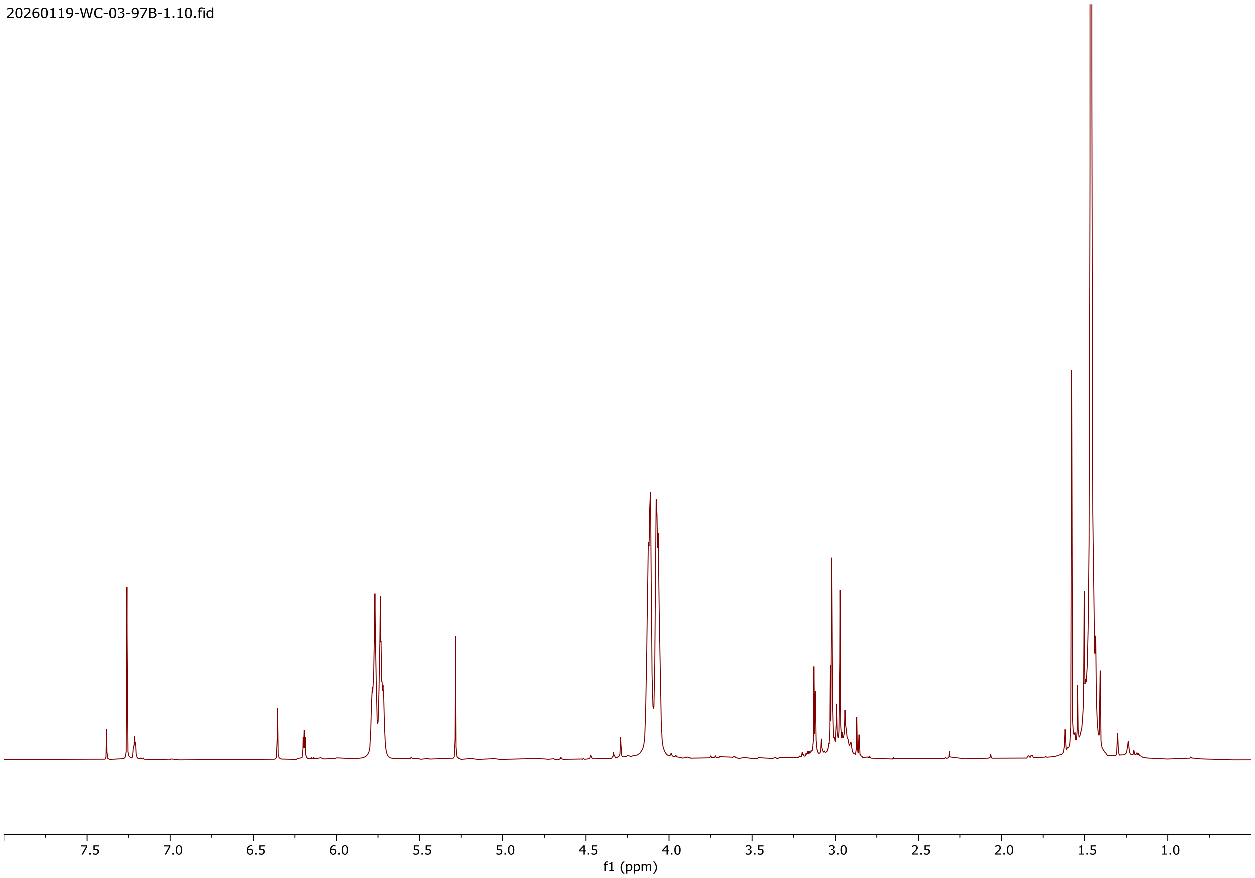

Figure S3. Crude ^{1}H NMR of cyclopropanation with 10

Reaction conducted with 3 eq of trap **5**, taking the crude ^{1}H NMR prior to Kugelrohr distillation.

Diazo **10** was fully consumed, as evidenced by missing signals at 4.95 (s, 1H) and 2.91 (s, 6H).

Signals at 5.83 – 5.69 (m, 1H), 4.09 (dtd, *J* = 21.3, 5.3, 2.8 Hz, 2H), and 1.46 (s, 9H) assigned as unreacted trap **5**.

No signals observed for intramolecular C-H insertion product (1-methylazetidin-2-one)[8]

No signals observed for diazo dimerization product (N^1,N^1,N^4,N^4-Tetramethylfumaramide)[9]

4. References

(1) Green, S. P.; Wheelhouse, K. M.; Payne, A. D.; Hallett, J. P.; Miller, P. W.; Bull, J. A. Thermal Stability and Explosive Hazard Assessment of Diazo Compounds and Diazo Transfer Reagents. *Organic Process Research & Development* **2020**, *24*, 67–84.

(2) Nguyen, T. H.; Navarro, A.; Ruble, J. C.; Davies, H. M. L. Stereoselective Synthesis of Either Exo- or Endo-3-Azabicyclo[3.1.0]hexane-6-carboxylates by Dirhodium(II)-Catalyzed Cyclopropanation with Ethyl Diazoacetate under Low Catalyst Loadings. *Org. Lett.* **2024**, *26*, 2832–2836.

(3) Davies, H. M. L.; Bruzinski, P. R.; Lake, D. H.; Kong, N.; Fall, M. J. Asymmetric Cyclopropanations by Rhodium(II) N-(Arylsulfonyl)prolinate Catalyzed Decomposition of Vinyldiazomethanes in the Presence of Alkenes. Practical Enantioselective Synthesis of the Four Stereoisomers of 2-Phenylcyclopropan-1-amino Acid. *J. Am. Chem. Soc.* **1996**, *118*, 6897–6907.

(4) Fu, J.; Ren, Z.; Bacsa, J.; Musaev, D. G.; Davies, H. M. L. Desymmetrization of cyclohexanes by site- and stereoselective C-H functionalization. *Nature* **2018**, *564*, 395–399.

(5) Reddy, R. P.; Davies, H. M. Dirhodium tetracarboxylates derived from adamantylglycine as chiral catalysts for enantioselective C-h aminations. *Org. Lett.* **2006**, *8*, 5013–5016.

(6) Qin, C.; Davies, H. M. Role of sterically demanding chiral dirhodium catalysts in site-selective C-H functionalization of activated primary C-H bonds. *J. Am. Chem. Soc.* **2014**, *136*, 9792–9796.

(7) Ghanem, A.; Gardiner, M. G.; Williamson, R. M.; Muller, P. First X-ray structure of a N-naphthaloyl-tethered chiral dirhodium(II) complex: structural basis for tether substitution improving asymmetric control in olefin cyclopropanation. *Chemistry* **2010**, *16*, 3291–3295.

(8) Chavelas-Hernández, L.; Valdéz-Camacho, J. R.; Hernández-Vázquez, L. G.; Dominguez-Mendoza, B. E.; Vasquez-Ríos, M. G.; Escalante, J. A New Approach Using Aromatic-Solvent-Induced Shifts in NMR Spectroscopy to Analyze β-Lactams with Various Substitution Patterns. *Synlett* **2019**, *31*, 158–164.

(9) Kinnell, A.; Harman, T.; Bingham, M.; Berry, A.; Nelson, A. Development of an organo- and enzyme-catalysed one-pot, sequential three-component reaction. *Tetrahedron* **2012**, *68*, 7719–7722.

(10) Jun, J. V.; Petri, Y. D.; Erickson, L. W.; Raines, R. T. Modular Diazo Compound for the Bioreversible Late-Stage Modification of Proteins. *J. Am. Chem. Soc.* **2023**, *145*, 6615–6621.

(11) Jun, J. V.; Raines, R. T. Two-Step Synthesis of alpha-Aryl-alpha-diazoamides as Modular Bioreversible Labels. *Org. Lett.* **2021**, *23*, 3110–3114.

(12) Gupta, A. K.; Yin, X.; Mukherjee, M.; Desai, A. A.; Mohammadlou, A.; Jurewicz, K.; Wulff, W. D. Catalytic Asymmetric Epoxidation of Aldehydes with Two VANOL-Derived Chiral Borate Catalysts. *Angew. Chem. Int. Ed.* **2019**, *58*, 3361–3367.

(13) Doben, N.; Yan, H.; Kischkewitz, M.; Mao, J.; Studer, A. Intermolecular Acetoxyaminoalkylation of alpha-Diazo Amides with (Diacetoxyiodo)benzene and Amines. *Org. Lett.* **2018**, *20*, 7933–7936.

(14) Xie, Y.; Zhang, L. H.; Xuan, J. Photoinduced Formal Cross-[3+3] Cycloaddition of Vinyldiazo Reagents with Acceptor-Only Diazoalkanes. *Org. Lett.* **2025**, *27*, 3117–3122.

(15) Munaretto, L. S.; Gallo, R. D. C.; Leao, L.; Jurberg, I. D. H-F bond insertions into alpha-diazo carbonyl compounds. *Org. Biomol. Chem.* **2022**, *20*, 6178–6182.

(16) Li, W.; He, M.; Zhou, Y.; Fu, R. 3-aza-bicyclo[3.1.0]hexane derivative, preparation method therefor and use thereof. WO2024245077A1, 2024.

(17) Xie, H.; Huang, E.; Fang, Q.; Zhang, Y. Pyrimidopyrazole compound, and pharmaceutical composition thereof and use thereof. WO2025067290A1, 2025.

5. Procedures and Compound Characterizations

General Procedure A: Synthesis of diazoacetamides from **24**

To a flame-dried 20 mL vial equipped with a stir bar was added **24** (1.0 eq, 2.0 mmol, 366 mg) and CH_2Cl_2 (0.5 M, 4.0 mL, SPS grade). The vial was sealed with a septa-lined cap and the reaction mixture was sparged with argon for 5 min, to which TEA (1.0 eq, 2.0 mmol, 202 mg, 0.28 mL) and **amine** were added sequentially. The reaction was then stirred at 25°C for 18 h, after which the mixture was filtered through celite, washed with CH_2Cl_2, and concentrated *in vacuo*. The crude residue was purified by silica gel flash column chromatography. The fractions containing product were combined and concentrated *in vacuo* to yield the desired diazo product.

General Procedure B: Cyclopropanation with **5**

To a flame-dried 4 mL vial equipped with a stir bar and activated 4 Å Mol Sieves (100 wt%, 250 mg) was added **5** (10.0 eq, 2.5 mmol, 423 mg), Rh_2(*S*-NTTL)$_4$ (1 mol%, 2.5 μmol, 3.6 mg), and CH_2Cl_2 (0.25 M to diazo, 1.0 mL, distilled). In a separate flame-dried 4 mL vial was added the **diazo** (1.0 eq, 0.25 mmol) and CH_2Cl_2 (0.25 M to diazo, 1.0 mL, distilled). Both vials were sealed with a septa-lined cap and sparged with argon for 5 min. The diazo solution was then added to the catalyst solution via dual syringe pump over 3 h [settings: 1 mL syringe, diameter 4.71 mm; Air-Tite/SilverPoint 22 G x 4" long hypodermic needle] and subsequently stirred for 15 h. After the elapsed time, the reaction solution was filtered through a plug of celite, washed with CH_2Cl_2, and concentrated *in vacuo*. The crude residue was subjected to a Kugelrohr distillation at 0.16 bar (120 torr) and 110°C for at least 1 h to distill off the unreacted trap **5**. The remaining residue after Kugelrohr distillation was purified by silica gel flash column chromatography to yield the desired cyclopropanation product.

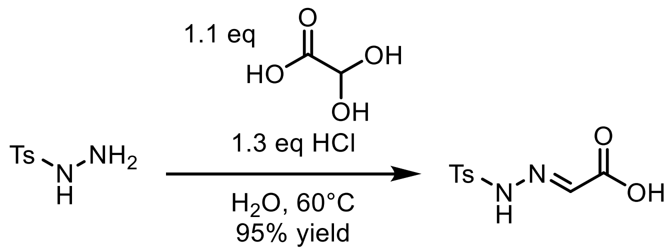

(*E*)-2-(2-tosylhydrazineylidene)acetic acid (SI-1)

To a 2 L beaker charged with an overhead stirrer [**Note**: *we observed that this was optimal for stirring due to clumpy suspension being formed in the reaction*] and a temperature probe, was added 2-oxoacetic acid hydrate (1.1 eq, 440 mmol, 40.5 g) and water (1.1 M to acid, 400.0 mL). The solution was vigorously stirred and heated to 60°C. The homogenous, clear, pale-yellow solution was stirred at 60°C for at least 10 min. To this solution was added 4-methylbenzenesulfonohydrazide (1.0 eq, 400 mmol, 74.5 g) divided in four portions to allow for even stirring [**Note:** *adding all hydrazide in one portion results in clumping and inadequate stirring*]. After complete addition of the hydrazide, HCl (1.3 eq, 520 mmol, 2.5 M aqueous, 208.0 mL) was added to the reaction mixture and the solution was left to vigorously stir at 60°C for 1 h. After the elapsed time, the heating was turned off so that the mixture could cool to 25°C on the heating plate over 3 h while stirring [**Note:** *one run was performed where the reaction mixture was taken off the heating block immediately and stirred at 25°C for 2 h. This resulted in a 13:1 ratio of product:hydrazide*]. The suspension was vacuum filtered using a Büchner funnel fitted with a filter paper, washing with excess water. The filter cake on the Büchner funnel was vacuum-dried open to air overnight to afford **SI-1** (0.38 mol, 93 g) as a white solid in 95% yield. The ^{1}H NMR is in good agreement with literature characterizations.[10]

^{1}H NMR (400 MHz, DMSO-*d*$_6$) δ 12.29 (s, 1H), 7.71 (d, *J* = 8.3 Hz, 2H), 7.44 (d, *J* = 8.3 Hz, 2H), 7.18 (s, 1H), 2.39 (s, 3H).

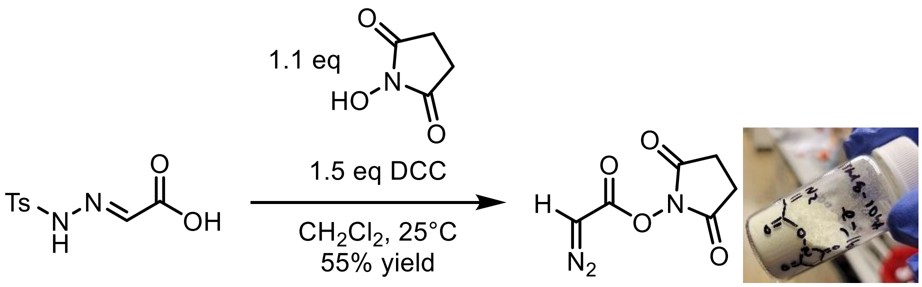

2,5-dioxopyrrolidin-1-yl 2-diazoacetate (24)

**Fresh bottles of DCC and NHS were used and dichloromethane was distilled from CaH_2 onto molecular sieves. Adapted as a modified procedure by Raines.*[11]

To a flame-dried 500 mL RBF charged with a stir-bar was added **SI-1** (1.0 eq, 13.33 mmol, 3.2299 g) and N-Hydroxysuccinimide (1.1 eq, 14.67 mmol, 1.6879 g). This was diluted with CH_2Cl_2 (0.1 M to acid, 133.3 mL, distilled) and cooled to 0°C. A separately prepared and degassed solution of DCC (1.5 eq, 20.0 mmol, 4.1265 g) in CH_2Cl_2 (1 M to DCC, 20.0 mL, distilled) was then added dropwise to the reaction and subsequently stirred at 0°C for 1 h. The reaction was allowed to naturally warm to 25°C over 18 h. After the elapsed time, the reaction was quenched by filtering through a plug of celite, washing with CH_2Cl_2 and the filtrate concentrated *in vacuo*. The concentrated filtrate was diluted with 50 mL of sat. aq. $NaHCO_3$ solution and 50 mL of CH_2Cl_2 then extracted with 50 mL of CH_2Cl_2 three times. The combined organic phases were washed with 30 mL of sat. aq. $NaHCO_3$ three times then washed with 50 mL of brine. The combined organic phases were dried with Na_2SO_4, filtered, and concentrated *in vacuo*. The crude residue was purified by silica gel flash column chromatography, eluting with a 6:4:1 Hex:EtOAc:CH_2Cl_2 eluent mixture. The collected fractions were concentrated *in vacuo* to afford **24** (7.3 mmol, 1405.6 mg) as a light-yellow solid in 55% yield. When the solids are powdered, the product is an off-white powder. The ^{1}H NMR is in good agreement with literature characterizations.[11]

R_f = 0.3, 50% EtOAc in Hex, 254 nm UV visualization, vanillin stain (red)

^{1}H NMR (400 MHz, $CDCl_3$) δ 5.12 (brs, 1H), 2.85 (s, 4H).

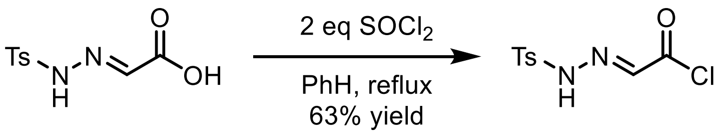

2-(2-Tosylhydrazineylidene)acetyl chloride (SI-2)

To a flame-dried 250 mL RBF equipped with a stir bar was added **SI-1** (1.0 eq, 64.81 mmol, 15.7 g). This was degassed and backfilled with argon three times, then left under an argon atmosphere via balloon. Benzene (0.86 M, 75.0 mL, reagent grade) was added and the reaction mixture was heated to reflux for 5 min. To this, $SOCl_2$ (2.0 eq, 129.6 mmol, 15.42g, 9.46 mL) was added and the reaction mixture was further stirred at reflux for 2 h, until the solids were dissolved. The reaction mixture was then cooled to 25°C and concentrated *in vacuo*. The solids were filtered through a Büchner funnel fitted with filter paper, washing thoroughly with benzene to yield **SI-2** (10.56 g, 40.51 mmol) as a white solid in 63% yield. The ^{1}H NMR is in good agreement with literature characterizations.[12]

^{1}H NMR (400 MHz, $CDCl_3$) δ 9.11 (s, 1H), 7.87 (d, *J* = 8.4 Hz, 2H), 7.38 (d, *J* = 8.1 Hz, 2H), 7.23 (d, *J* = 0.9 Hz, 1H), 2.46 (s, 3H)

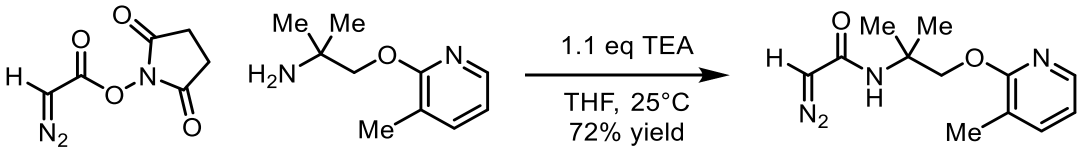

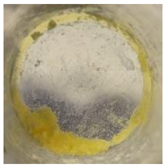

2-diazo-N-(2-methyl-1-((3-methylpyridin-2-yl)oxy)propan-2-yl)acetamide (8)
2-methyl-1-((3-methylpyridin-2-yl)oxy)propan-2-amine was graciously provided to us by Lilly.

To a flame-dried 4 mL vial equipped with a stir-bar was added 2-methyl-1-((3-methylpyridin-2-yl)oxy)propan-2-amine (1.2 eq, 0.6 mmol, 108.0 mg) and **24** (1.0 eq, 0.5 mmol, 91.6 mg). This was degassed and backfilled with argon three times, then topped with an argon balloon. The reaction was diluted with CH_2Cl_2 (0.2 M to diazo, 2.5 mL, SPS Grade), after which the reaction solution was stirred at 25°C for at least 5 min. Afterwards, TEA (1.1 eq, 0.55 mmol, 55.7 mg, 77 µL) was added to the mixture and the reaction was left to stir at 25°C for 15 h. After the elapsed time, the reaction solution was directly concentrated *in vacuo*. The crude residue was then purified by silica gel flash column chromatography, eluting 25% EtOAc in Hex. The collected fractions were concentrated *in vacuo* to afford **8** (89.5 mg, 0.36 mmol) as a yellow solid in 72% yield.

R_f = 0.29, 50% EtOAc in Hex, 254 nm UV visualization

^{1}H NMR (400 MHz, CDCl$_3$) δ 7.98 – 7.93 (m, 1H), 7.50 – 7.36 (m, 1H), 6.83 (dd, *J* = 7.2, 5.0 Hz, 1H), 6.25 (brs, 1H), 4.57 (s, 1H), 4.31 (s, 2H), 2.21 (s, 3H), 1.52 (s, 6H).

^{13}C NMR (101 MHz, CDCl$_3$) δ 164.8, 162.2, 143.7, 139.0, 121.1, 117.3, 72.8, 54.9, 47.6, 23.9, 15.9.

HRMS (+p APCI) calculated for $C_{12}H_{17}N_4O_2$ (M+H) 249.1353, found 249.1351

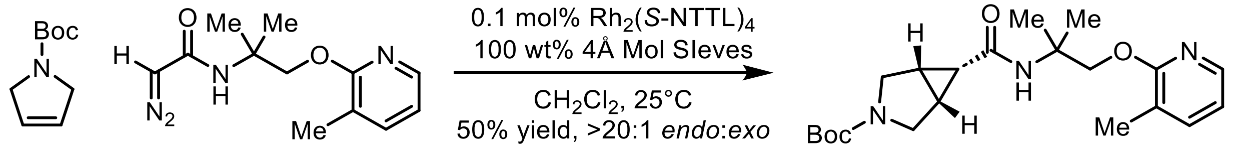

***tert*-Butyl *endo*-6-((2-methyl-1-((3-methylpyridin-2-yl)oxy)propan-2-yl)carbamoyl)-3-azabicyclo[3.1.0]hexane-3-carboxylate (*endo*-9)**

The procedure outlined here applies for the scale-up synthesis shown in Scheme 2 in the main text. The reactions for Figure 4 in the main text followed General Procedure B, deviating the reaction conditions as appropriate.

To a flame-dried 50 mL RBF equipped with a stir-bar was added activated 4Å Mol Sieves (100 wt%, 2.0 g), **5** (10 eq, 20.0 mmol, 3.38 g), and Rh_2(*S*-NTTL)$_4$ (0.1 mol%, 2.9 mg). The vessel was degassed and backfilled with argon three times, then left under an argon atmosphere via balloon. This was diluted with CH_2Cl_2 (0.25 M to diazo, 8.0 mL, distilled), and left to stir at 25°C for 10 min. To this, a previously-prepared solution of **8** (1.0 eq, 2.0 mmol, 497 mg) in CH_2Cl_2 (0.25 M to diazo, 8.0 mL, distilled) was added dropwise over 30 sec (**Note:** *subtle bubbling was observed*). After complete addition of the catalyst, the reaction vessel was left to stir for 18 h. After the elapsed time, the reaction solution was filtered through a plug of celite, washing with CH_2Cl_2, and the filtrate concentrated *in vacuo*. The crude material was subjected to a Kugelrohr distillation at 110°C for at least 1 h to distill off the unreacted trap. The resulting pot residue is an orange-red solid. The crude material was dry-loaded onto silica gel and purified by automated flash column chromatography via Biotage. The fractions containing product were combined and concentrated *in vacuo* to afford *endo*-**9** (393 mg, 1.01 mmol) as a clear light tan viscous oil in 50% yield and >20:1 d.r. (*endo*:*exo*).

Purification Gradient:
0% Et_2O in Hex [3 CV] → 0 to 100% Et_2O in Hex [5 CV] →100% Et_2O in Hex [5 CV]

^{1}H NMR (400 MHz, $CDCl_3$) δ 7.94 (ddd, *J* = 5.1, 2.0, 0.8 Hz, 1H), 7.42 (ddt, *J* = 7.1, 1.6, 0.8 Hz, 1H), 6.81 (dd, *J* = 7.1, 5.0 Hz, 1H), 6.77 (s, 1H), 4.28 (d, *J* = 1.9 Hz, 2H), 3.75 (dd, *J* = 21.6, 11.0 Hz, 2H), 3.43 – 3.24 (m, 2H), 2.24 (s, 3H), 1.68 (td, *J* = 9.4, 5.5 Hz, 3H), 1.45 (d, *J* = 22.4 Hz, 6H), 1.39 (s, 9H).

^{13}C NMR (101 MHz, $CDCl_3$) δ 166.2, 162.2, 154.1, 143.5, 139.0, 121.2, 117.2, 79.3, 73.0, 54.3, 45.2, 45.1, 28.4, 24.2, 23.2, 22.8, 20.6, 19.9, 16.0.

HRMS (+p APCI) calculated for $C_{21}H_{32}N_3O_4$ (M+H) 390.2395, found 390.2396

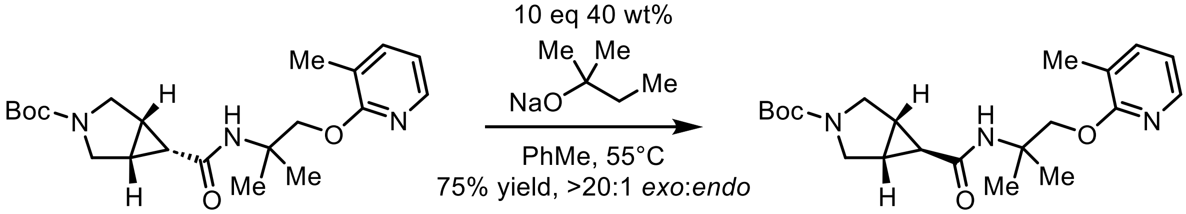

***tert*-Butyl *exo*-6-((2-methyl-1-((3-methylpyridin-2-yl)oxy)propan-2-yl)carbamoyl)-3-azabicyclo[3.1.0]hexane-3-carboxylate (*exo*-9)**

To a flame-dried vial charged with a stir-bar was added *endo*-**9** (1.0 eq, 986 μmol, 384 mg) and sodium *tert*-pentoxide (10 eq, 9.86 mmol, 2.71 g, 40% in toluene) open to air. The reaction vessel was sealed, topped with a nitrogen balloon, heated at 55°C for 18 h. After the elapsed time, the solution was diluted with 50 ml of sat. aq. $NaHCO_3$ and extracted with 50 mL of EtOAc three times. The combined organic phases were washed with 50 mL of brine, dried over $MgSO_4$, filtered, and concentrated *in vacuo*. The crude material was dry-loaded onto silica gel and purified by automated flash chromatography via Biotage, eluting on a gradient. The fractions containing product were combined and concentrated *in vacuo* to afford *exo*-**9** [286 mg, 0.734 mmol] as a clear light tan viscous oil in 75% yield and >20:1 d.r. (*exo*:*endo*).

Purification Gradient:
0% Et_2O in Hex [3 CV] → 0 to 100% Et_2O in Hex [5 CV] →100% Et_2O in Hex [5 CV]

^{1}H NMR (400 MHz, $CDCl_3$) δ 7.95 (dd, J = 5.1, 1.9 Hz, 1H), 7.41 (brd, 1H), 6.82 - 6.80 (m, 1H), 6.53 (brs, 1H), 4.31 (m, 2H), 3.61 (d, J = 11.1 Hz, 1H), 3.53 (d, J = 11.1 Hz, 1H), 3.38 (m, 2H), 2.21 (s, 3H), 1.96 (brm, 2H), 1.47 (s, 6H), 1.42 (s, 9H), 1.08 (t, J = 3.1 Hz, 1H).

^{13}C NMR (101 MHz, $CDCl_3$) δ 170.5, 162.1, 154.8, 143.8, 138.9, 121.0, 117.2, 79.5, 72.2, 54.2, 48.1, 47.8, 28.5, 27.2, 25.0, 24.4, 23.9, 23.7, 15.9.

HRMS (-p APCI) calculated for $C_{21}H_{30}N_3O_4$ (M-H) 388.2235, found 388.2226

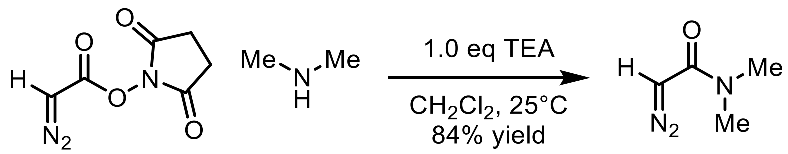

2-Diazo-N,N-dimethylacetamide (10)

Synthesized according to **General Procedure A** from dimethylamine (1.0 eq, 2.0 mmol, 0.55 mL, 40 wt% in water). The crude residue was purified via silica gel flash column chromatography, eluting 60% EtOAc in Hex. The collected fractions were concentrated *in vacuo* to afford **10** (190.6 mg, 1.68 mmol) as a yellow oil in 84% yield. The ^{1}H NMR is in good agreement with literature characterizations.[13]

^{1}H NMR (400 MHz, $CDCl_3$) δ 4.95 (s, 1H), 2.91 (s, 6H).

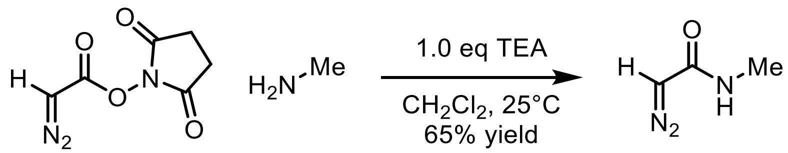

2-Diazo-N-methylacetamide (SI-3)

Synthesized according to **General Procedure A** from methylamine (5.0 eq, 10.0 mmol, 5.0 mL, 2.0 M in THF). The crude residue was purified via silica gel flash column chromatography, eluting 80% EtOAc in Hex. The collected fractions were concentrated *in vacuo* to afford **SI-3** (128.6 mg, 1.30 mmol) as a yellow solid in 65% yield.

^{1}H NMR (400 MHz, $CDCl_3$) δ 5.45 (s, 1H), 4.77 (s, 1H), 2.84 (d, J = 4.9 Hz, 3H).

^{13}C NMR (101 MHz, $CDCl_3$) δ 166.4, 47.1, 26.8.

HRMS (-p APCI) calculated for $C_3H_4N_3O$ (M-H) 98.0353, found 98.0368

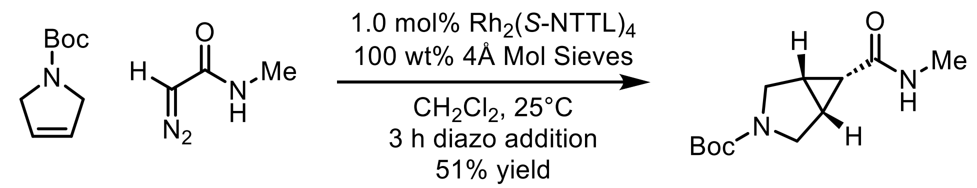

***tert*-Butyl *endo*-6-(methylcarbamoyl)-3-azabicyclo[3.1.0]hexane-3-carboxylate (*endo*-11)**

Synthesized according to **General Procedure B** from **SI-3** (1.0 eq, 0.25 mmol, 24.8 mg). The crude residue was purified via silica gel flash column chromatography eluting 100% EtOAc to yield ***endo*-11** (30.4 mg, 0.13 mmol) as a white solid in 51% yield.

^{1}H NMR (400 MHz, CDCl$_3$) δ 5.47 (s, 1H), 3.83 (dd, J = 12.0, 6.5 Hz, 2H), 3.47 – 3.25 (m, 2H), 2.75 (d, J = 4.9 Hz, 3H), 1.83 – 1.65 (m, 3H), 1.42 (s, 9H).

^{13}C NMR (101 MHz, CDCl$_3$) δ 167.9, 154.2, 79.6, 45.3 (d, J = 22.2 Hz), 28.6, 26.4, 23.4, 20.5 (d, J = 66.8 Hz).

HRMS (-p APCI) calculated for $C_{12}H_{19}N_2O_3$ (M-H) 239.1394, found 239.1405

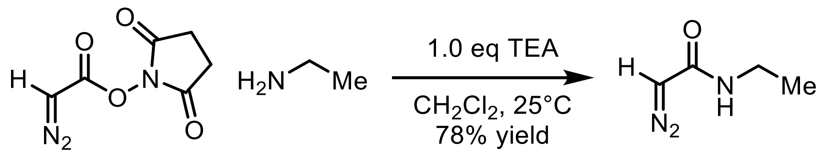

2-Diazo-N-ethylacetamide (SI-4)

Synthesized according to **General Procedure A** from ethylamine (2.0 eq, 4.0 mmol, 2.0 mL, 2.0 M in MeCN). The crude residue was purified via silica gel flash column chromatography, eluting 60% EtOAc in Hex. The collected fractions were concentrated *in vacuo* to afford **SI-4** (177.2 mg, 1.57 mmol) as a yellow solid in 78% yield. The ^{1}H NMR is in good agreement with literature characterizations.[14]

^{1}H NMR (400 MHz, CDCl$_3$) δ 5.31 (s, 1H), 4.73 (s, 1H), 3.44 – 3.18 (m, 2H), 1.14 (t, J = 7.3 Hz, 3H).

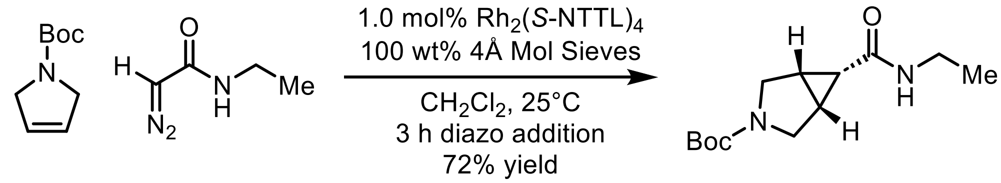

***tert*-Butyl *endo*-6-(ethylcarbamoyl)-3-azabicyclo[3.1.0]hexane-3-carboxylate (*endo*-12)**

Synthesized according to **General Procedure B** from **SI-4** (1.0 eq, 0.25 mmol, 28.3 mg). The crude residue was purified via silica gel flash column chromatography eluting 60% EtOAc in Hex to yield ***endo*-12** (45.5 mg, 0.18 mmol) as a white solid in 72% yield.

^{1}H NMR (400 MHz, $CDCl_3$) δ 5.51 (s, 1H), 3.89 – 3.73 (m, 2H), 3.48 – 3.29 (m, 2H), 3.29 – 3.12 (m, 2H), 1.81 – 1.65 (m, 3H), 1.40 (s, 9H), 1.10 (t, *J* = 7.3 Hz, 3H).

^{13}C NMR (101 MHz, $CDCl_3$) δ 167.0, 154.2, 79.5, 45.5 (d, *J* = 15.7 Hz), 34.4, 28.5, 23.5, 20.5 (d, *J* = 65.8 Hz), 15.0.

HRMS (-p APCI) calculated for $C_{13}H_{21}N_2O_3$ (M-H) 253.1550, found 253.1556

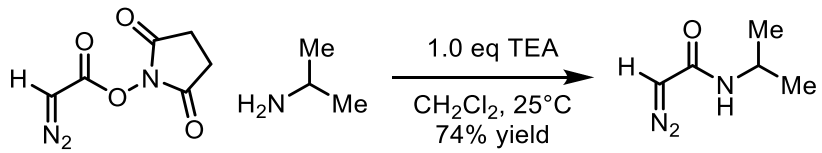

2-Diazo-N-isopropylacetamide (SI-5)

Synthesized according to **General Procedure A** from isopropylamine (1.2 eq, 2.4 mmol, 140 mg, 0.20 mL). The crude residue was purified via silica gel flash column chromatography eluting 40% EtOAc in Hex to yield **SI-5** (188 mg, 1.48 mmol) as a yellow solid in 74% yield.

^{1}H NMR (400 MHz, CDCl$_3$) δ 5.34 (s, 1H), 4.75 (s, 1H), 4.07 (s, 1H), 1.15 (d, *J* = 6.6 Hz, 6H).

^{13}C NMR (101 MHz, CDCl$_3$) δ 165.0, 47.1, 42.1, 23.1.

HRMS (-p APCI) calculated for $C_5H_8N_3O$ (M-H) 126.0666, found 126.0677

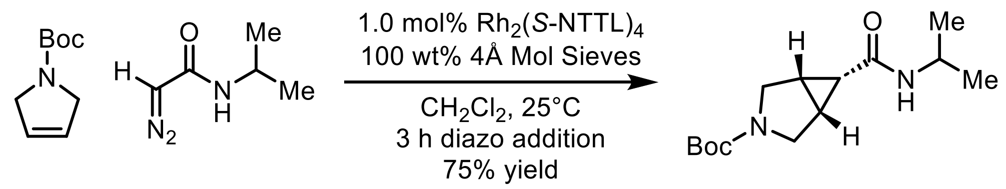

***tert*-Butyl *endo*-6-(isopropylcarbamoyl)-3-azabicyclo[3.1.0]hexane-3-carboxylate (*endo*-13)**

Synthesized according to **General Procedure B** from **SI-5** (1.0 eq, 0.25 mmol, 31.8 mg). The crude residue was purified via silica gel flash column chromatography eluting 70% EtOAc in Hex to yield ***endo*-13** (50.2 mg, 0.19 mmol) as a white solid in 75% yield.

^{1}H NMR (400 MHz, $CDCl_3$) δ 5.30 (d, J = 7.9 Hz, 1H), 3.99 (dp, J = 7.9, 6.5 Hz, 1H), 3.78 (t, J = 11.3 Hz, 2H), 3.42 (d, J = 11.4 Hz, 1H), 3.34 (dd, J = 11.1, 4.2 Hz, 1H), 1.83 – 1.64 (m, 3H), 1.40 (s, 9H), 1.12 (d, J = 6.6 Hz, 6H).

^{13}C NMR (101 MHz, $CDCl_3$) δ 166.1, 154.1, 79.5, 45.4 (d, J = 8.3 Hz), 41.5, 28.5, 23.6, 22.9, 20.5 (d, J = 66.9 Hz).

HRMS (-p APCI) calculated for $C_{14}H_{23}N_2O_3$ (M-H) 267.1707, found 267.1716

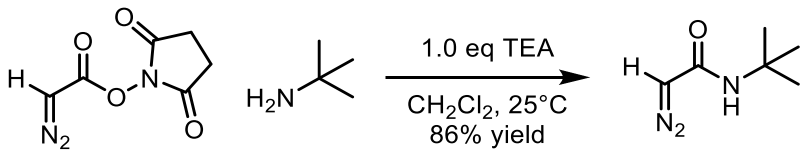

N-(*tert*-butyl)-2-diazoacetamide (20)

Synthesized according to **General Procedure A** from *tert*-butylamine (1.2 eq, 2.4 mmol, 176 mg, 0.25 mL). The crude residue was purified via silica gel flash column chromatography eluting 25% EtOAc in Hex to yield **20** (243.5 mg, 1.73 mmol) as a yellow solid in 86% yield.

^{1}H NMR (400 MHz, CDCl$_3$) δ 5.23 (s, 1H), 4.71 (s, 1H), 1.35 (s, 9H).

^{13}C NMR (101 MHz, CDCl$_3$) δ 165.0, 52.0, 47.6, 29.3.

HRMS (-p APCI) calculated for $C_6H_{10}N_3O$ (M-H) 140.0822, found 140.0833

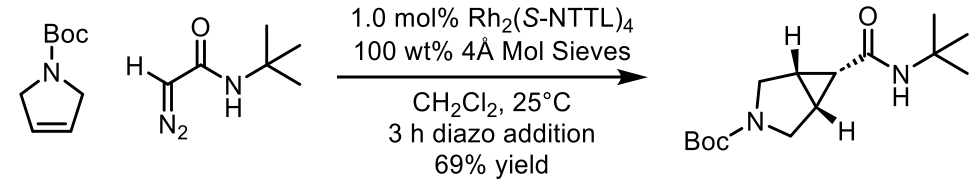

***tert*-Butyl *endo*-6-(*tert*-butylcarbamoyl)-3-azabicyclo[3.1.0]hexane-3-carboxylate (*endo*-14)**

Synthesized according to **General Procedure B** from **20** (1.0 eq, 0.25 mmol, 35.3 mg). The crude residue was purified via silica gel flash column chromatography eluting 60% EtOAc in Hex to yield ***endo*-14** (48.9 mg, 0.17 mmol) as a white solid in 69% yield.

^{1}H NMR (400 MHz, $CDCl_3$) δ 5.31 (s, 1H), 3.74 (dd, *J* = 25.0, 11.0 Hz, 2H), 3.42 (dt, *J* = 11.2, 2.3 Hz, 1H), 3.32 (dd, *J* = 10.7, 4.3 Hz, 1H), 1.77 – 1.63 (m, 3H), 1.38 (s, 9H), 1.28 (s, 9H).

^{13}C NMR (101 MHz, $CDCl_3$) δ 166.1, 154.2, 79.5, 51.4, 45.4, 28.8, 28.5, 24.3, 20.4 (d, *J* = 66.0 Hz).

HRMS (-p APCI) calculated for $C_{15}H_{25}N_2O_3$ (M-H) 281.1863, found 281.1865

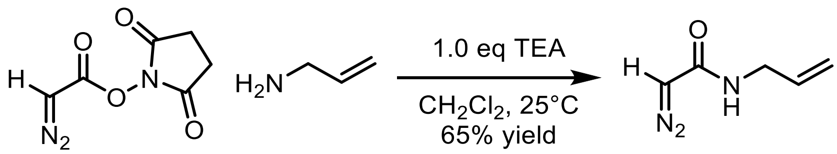

N-allyl-2-diazoacetamide (SI-6)

Synthesized according to **General Procedure A** from prop-2-ene-1-amine (1.2 eq, 2.40 mmol, 137 mg, 0.180 mL). The crude residue was purified via silica gel flash column chromatography eluting 40% EtOAc in Hex to yield **SI-6** (163.2 mg, 1.30 mmol) as a yellow solid in 65% yield. The ^{1}H NMR is in good agreement with literature characterizations.[15]

^{1}H NMR (400 MHz, CDCl$_3$) δ 5.82 (ddt, *J* = 17.2, 10.3, 5.5 Hz, 2H), 5.66 (s, 1H), 5.18 (dq, *J* = 17.1, 1.6 Hz, 1H), 5.12 (dq, *J* = 10.3, 1.5 Hz, 1H), 4.83 (s, 2H), 3.89 (t, *J* = 5.9 Hz, 4H).

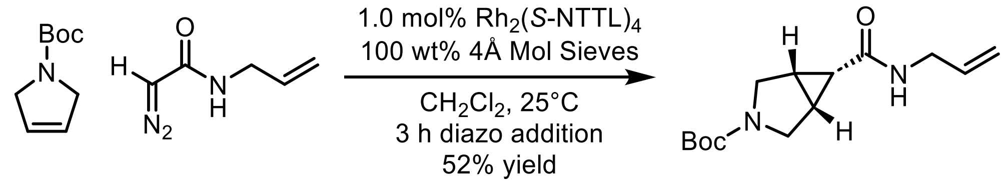

***tert*-Butyl *endo*-6-(allylcarbamoyl)-3-azabicyclo[3.1.0]hexane-3-carboxylate (*endo*-15)**

Synthesized according to **General Procedure B** from **SI-6** (1.0 eq, 0.25 mmol, 31.3 mg). The crude residue was purified via silica gel flash column chromatography eluting 80% EtOAc in Hex to yield ***endo*-15** (34.5 mg, 0.13 mmol) as a white solid in 52% yield.

^{1}H NMR (400 MHz, $CDCl_3$) δ 5.81 (ddt, J = 17.2, 10.2, 5.9 Hz, 1H), 5.56 (s, 1H), 5.17 (dq, J = 17.2, 1.6 Hz, 1H), 5.11 (dq, J = 10.2, 1.4 Hz, 1H), 3.92 – 3.72 (m, 4H), 3.47 – 3.27 (m, 2H), 1.74 (dq, J = 23.5, 7.1 Hz, 3H), 1.41 (s, 9H).

^{13}C NMR (101 MHz, $CDCl_3$) δ 167.1, 154.2, 134.5, 116.9, 79.6, 45.4 (d, J = 18.1 Hz), 42.1, 28.5, 23.4, 20.6 (d, J = 64.7 Hz).

HRMS (-p APCI) calculated for $C_{14}H_{21}N_2O_3$ (M-H) 265.1550, found 265.1558

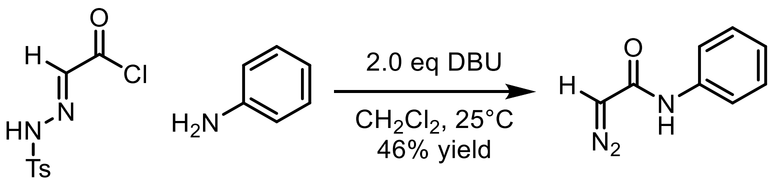

2-diazo-N-phenylacetamide (SI-7)

To a flame-dried 20 mL vial charged with a stir bar was added **SI-2** (1.0 eq, 2.0 mmol, 521.4 mg) and CH_2Cl_2 (0.4 M, 5 mL, distilled). The reaction mixture was stirred at 0°C for 5 min, after which aniline (1.1 eq, 2.2 mmol, 204.9 mg, 0.20 mL) was added, followed by DBU (2.0 eq, 4.0 mmol, 609 mg, 0.60 mL). The reaction mixture was stirred at 0°C for 18 h. Upon completion, the reaction mixture was washed with 10 mL of sat. aq. NH_4Cl and extracted with 20 mL of CH_2Cl_2 three times. The combined organic phases were washed with 20 mL of brine, dried over anhydrous Na_2SO_4, and concentrated *in vacuo.* The crude material was purified via silica gel flash column chromatography eluting 25% EtOAc in Hex. The eluting yellow fractions were combined and concentrated *in vacuo.* The resulting solids were then thoroughly washed with Et_2O through a Büchner funnel fitted with a filter paper to yield **SI-7** (149 mg, 0.93 mmol) as a yellow solid in 46% yield. The ^{1}H NMR is in good agreement with literature characterizations.[12]

^{1}H NMR (400 MHz, $CDCl_3$) δ 7.42 (d, J = 8.0 Hz, 2H), 7.32 (t, J = 7.9 Hz, 2H), 7.12 (t, J = 7.4 Hz, 1H), 6.69 (s, 1H), 4.89 (s, 1H).

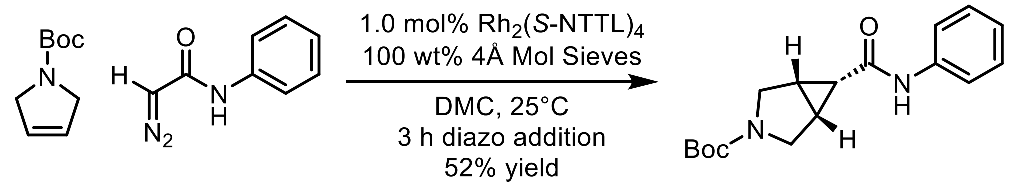

***tert*-Butyl *endo*-6-(phenylcarbamoyl)-3-azabicyclo[3.1.0]hexane-3-carboxylate (*endo*-16)**

Synthesized according to a modified **General Procedure B** utilizing instead DMC (distilled) as solvent instead of CH_2Cl_2. The crude material was purified via silica gel flash column chromatography eluting 50% EtOAc in Hex to yield ***endo*-16** (38.9 mg, 0.13 mmol) as a white solid in 52% yield.

^{1}H NMR (400 MHz, $CDCl_3$) δ 7.51 – 7.44 (m, 2H), 7.43 (s, 1H), 7.25 (t, J = 7.9 Hz, 2H), 7.05 (t, J = 7.4 Hz, 1H), 3.94 (s, 2H), 3.42 (d, J = 11.2 Hz, 2H), 1.85 (h, J = 7.5 Hz, 3H), 1.27 (s, 9H).

^{13}C NMR (101 MHz, $CDCl_3$) δ 165.8, 154.1, 137.9, 128.9, 124.3, 120.2, 79.7, 45.4, 28.3, 24.4, 21.1 (d, J = 50.7 Hz).

HRMS (-p APCI) calculated for $C_{17}H_{21}N_2O_3$ (M-H) 301.1550, found 301.1560

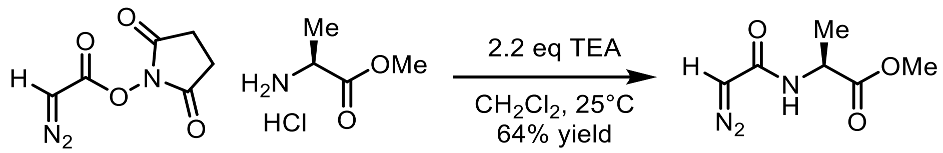

Methyl (2-diazoacetyl)-L-alaninate (SI-8)

Synthesized according to a modified **General Procedure A** utilizing instead methyl L-alaninate hydrochloride (1.0 eq, 2.0 mmol, 279 mg) and TEA (2.2 eq, 4.4 mmol, 445 mg, 0.61 mL). The crude material was purified via silica gel flash column chromatography eluting 40% EtOAc in Hex to yield **SI-8** (220 mg, 1.29 mmol) as a yellow oil in 64% yield.

^{1}H NMR (400 MHz, $CDCl_3$) δ 5.90 (s, 1H), 4.85 – 4.76 (m, 1H), 4.64 (tt, *J* = 9.8, 4.9 Hz, 1H), 3.78 – 3.72 (m, 3H), 1.41 (ddd, *J* = 7.3, 3.2, 2.4 Hz, 3H).

^{13}C NMR (101 MHz, $CDCl_3$) δ 174.0, 165.3, 52.7, 48.5, 47.5, 18.8.

HRMS (-p APCI) calculated for $C_6H_8N_3O_3$ (M-H) 170.0564, found 170.0575

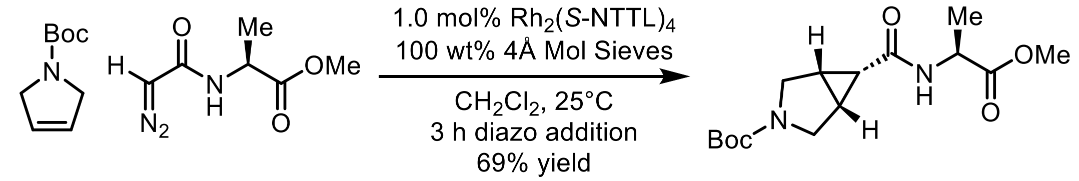

***tert*-Butyl *endo*-6-(((*S*)-1-methoxy-1-oxopropan-2-yl)carbamoyl)-3-azabicyclo[3.1.0]hexane-3-carboxylate (*endo*-17)**

Synthesized according to **General Procedure B** from **SI-8** (1.0 eq, 0.25 mmol, 42.8 mg). The crude material was purified via silica gel flash column chromatography eluting 70% EtOAc in Hex to yield ***endo*-17** (54 mg, 0.17 mmol) as a clear oil in 69% yield.

^{1}H NMR (400 MHz, CDCl$_3$) δ 6.21 – 6.11 (m, 1H), 4.52 (p, J = 7.2 Hz, 1H), 3.82 (d, J = 11.1 Hz, 1H), 3.76 (d, J = 11.3 Hz, 1H), 3.74 (d, J = 2.9 Hz, 3H), 3.45 – 3.28 (m, 2H), 1.84 – 1.67 (m, 3H), 1.38 (s, 9H), 1.35 (d, J = 7.1 Hz, 3H).

^{13}C NMR (101 MHz, CDCl$_3$) δ 173.8, 166.6 (d, *J* = 14.3 Hz), 154.1, 79.4, 52.6, 48.0, 45.8 – 45.2 (m), 28.5, 23.1, 20.7 (dd, *J* = 63.3, 22.7 Hz), 18.9 (d, *J* = 26.0 Hz).

HRMS (-p APCI) calculated for $C_{15}H_{23}N_2O_5$ (M-H) 311.1605, found 311.1612

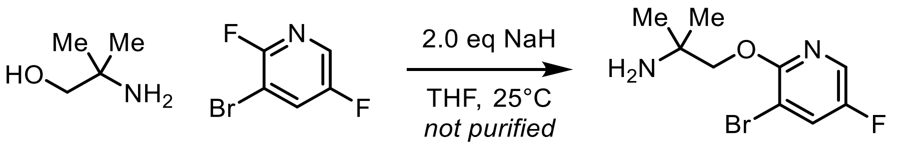

1-((3-Bromo-5-fluoropyridin-2-yl)oxy)-2-methylpropan-2-amine (SI-9)
SI-9 was prepared adapting a literature procedure.[16]

To a flame-dried 50 mL RBF equipped with a stir bar was added 2-amino-2-methyl-1-propanol (2.0 eq, 10.0 mmol, 891 mg) and NaH (2.0 eq, 10.0 mmol, 400 mg, 60 wt% in mineral oil). The solids were dissolved in THF (0.5 M to alcohol, 20.0 mL, SPS grade) and stirred at 25°C for 30 min. To this, a solution of 3-bromo-2,5-difluoropyridine (1.0 eq, 5.0 mmol, 970 mg) in THF (1.67 M to fluoride, 3.0 mL, SPS grade) was added to the reaction mixture. The resulting solution was stirred at 25°C for 2 h. Upon completion, the reaction mixture was added dropwise to 5 mL of a sat. aq. NH_4Cl solution in a separatory funnel. The aqueous layer was washed twice with 10 mL of EtOAc. The combined organic phases were washed with 20 mL of brine, dried over anhydrous Na_2SO_4, and concentrated *in vacuo*. The crude material was then re-dissolved in 10 mL of MeCN and washed three times with 10 mL of Hex. The combined MeCN phases were concentrated *in vacuo* to yield **SI-9** as a yellow oil which was used in the next step without further purification.

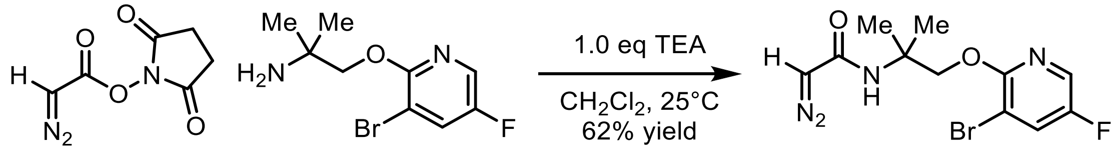

N-(1-((3-bromo-5-fluoropyridin-2-yl)oxy)-2-methylpropan-2-yl)-2-diazoacetamide (SI-10)

Synthesized according to **General Procedure A** from **SI-9** (1.2 eq, 2.4 mmol, 632 mg). The crude material was purified via silica gel flash column chromatography eluting 25% EtOAc in Hex to yield **SI-10** (409.8 mg, 1.24 mmol) as a yellow solid in 62% yield over two steps.

^{1}H NMR (400 MHz, CDCl$_3$) δ 7.93 (d, *J* = 2.7 Hz, 1H), 7.64 (dd, *J* = 7.0, 2.7 Hz, 1H), 5.35 (s, 1H), 4.66 (d, *J* = 1.5 Hz, 1H), 4.37 (s, 2H), 1.49 (s, 6H).

^{13}C NMR (101 MHz, CDCl$_3$) δ 165.3, 156.3 (d, *J* = 1.2 Hz), 154.5 (d, *J* = 252.1 Hz), 132.0 (d, *J* = 25.2 Hz), 130.0 (d, *J* = 23.0 Hz), 106.8 (d, *J* = 4.6 Hz), 73.0, 54.4, 47.8, 24.6.

^{19}F NMR (376 MHz, CDCl$_3$) δ -135.88 (d, *J* = 7.0 Hz, 1F).

HRMS (+p ESI) calculated for $C_{11}H_{12}BrFN_4O_2Na$ (M+Na) 353.0025, found 353.0012

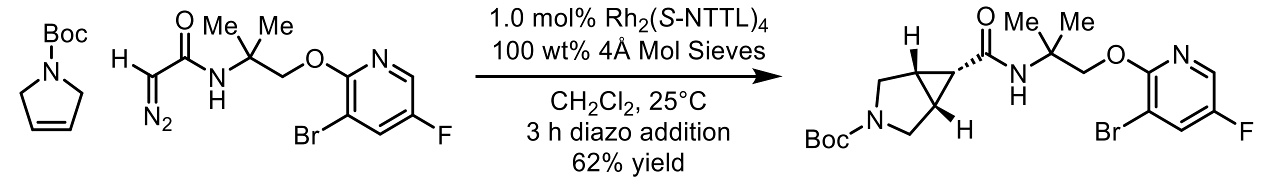

***tert*-Butyl *endo*-6-((1-((3-bromo-5-fluoropyridin-2-yl)oxy)-2-methylpropan-2-yl)carbamoyl)-3-azabicyclo[3.1.0]hexane-3-carboxylate (*endo*-18)**

Synthesized according to **General Procedure B** from **SI-10** (1.0 eq, 0.25 mmol, 82.8 mg). The crude material was purified via silica gel flash column chromatography eluting 60% EtOAc in Hex to yield ***endo*-18** (73.5 mg, 0.16 mmol) as a white solid in 62% yield.

^{1}H NMR (400 MHz, CDCl$_3$) δ 7.93 (d, J = 2.7 Hz, 1H), 7.64 (dd, J = 7.0, 2.8 Hz, 1H), 5.84 (s, 1H), 4.36 (d, J = 10.3 Hz, 1H), 4.27 (d, J = 10.4 Hz, 1H), 3.82 (d, J = 11.0 Hz, 2H), 3.43 (d, J = 11.2 Hz, 1H), 3.34 (d, J = 5.7 Hz, 1H), 1.75 – 1.67 (m, 3H), 1.43 (d, J = 10.9 Hz, 6H), 1.39 (s, 9H).

^{13}C NMR (101 MHz, CDCl$_3$) δ 166.7, 156.3, 154.5 (d, J = 252.0 Hz), 154.3, 131.9 (d, J = 25.2 Hz), 130.0 (d, J = 23.0 Hz), 106.8 (d, J = 4.6 Hz), 79.5, 73.1, 53.8, 45.4, 28.60, 24.30, 23.7, 20.6 (d, J = 66.3 Hz).

^{19}F NMR (376 MHz, CDCl$_3$) δ -136.05 (d, J = 7.0 Hz, 1F).

HRMS (+p APCI) calculated for $C_{20}H_{27}BrFN_3O_4$ (M+H) 472.1249, found 472.1256

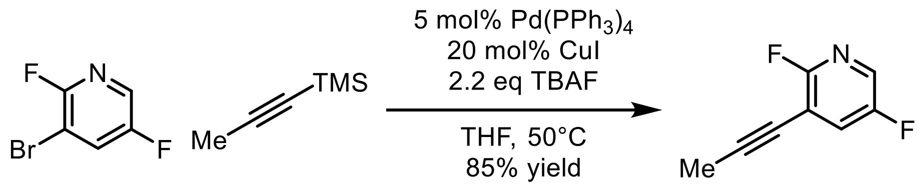

2,5-difluoro-3-(prop-1-yn-1-yl)pyridine (SI-11)
SI-11 was prepared adapting a literature procedure.[17]

To a flame-dried 50 mL RBF equipped with a stir bar was added 3-bromo-2,5-difluoropyridine (1.0 eq, 6.0 mmol, 1.16 g), $Pd(PPh_3)_4$ (5 mol%, 0.3 mmol, 347 mg), CuI (20 mol%, 1.2 mmol, 229 mg). This was degassed and backfilled with argon three times, then left under an argon atmosphere via balloon. This was diluted with THF (0.3 M, 20.0 mL, SPS grade), to which TBAF (2.2 eq, 13.2 mmol, 13.2 mL, 1.0 M in THF) and TEA (0.5 eq, 3.0 mmol, 304 mg, 0.42 mL) were added. The reaction mixture was sparged with argon for 10 min, then stirred at 50°C for 5 min. Then, trimethyl(prop-1-yn-1-yl)silane (2.0 eq, 12.0 mmol, 1.35 g, 1.78 mL) was added dropwise dual syringe pump over 1 h [settings: 2 mL syringe, diameter 9.83 mm; Air-Tite/SilverPoint 22 G x 4" long hypodermic needle]. The resulting mixture was stirred at 50°C for 18 h. Upon completion, the reaction mixture was concentrated *in vacuo*, then dissolved in 30 mL of CH_2Cl_2 and washed with 30 mL of sat. aq. NaCl. The combined organic phases were washed with 30 mL of brine, dried over anhydrous Na_2SO_4, and concentrated *in vacuo*. The crude residue was purified via silica gel flash column chromatography eluting 5% EtOAc in Hex to yield **SI-11** (777 mg, 5.07 mmol) as a yellow oil in 85% yield.

^{1}H NMR (400 MHz, $CDCl_3$) δ 7.93 (dd, J = 3.1, 1.8 Hz, 1H), 7.50 (td, J = 7.2, 3.0 Hz, 1H), 2.11 (s, 3H).

^{13}C NMR (101 MHz, $CDCl_3$) δ 158.9 (d, J = 239.0 Hz), 156.8 (dd, J = 252.0, 4.4 Hz), 133.1 (dd, J = 27.4, 15.3 Hz), 130.3 (dd, J = 22.2, 3.6 Hz), 109.2 (dd, J = 36.2, 6.7 Hz), 95.0 (d, J = 2.1 Hz), 71.0 (dd, J = 5.6, 2.0 Hz), 4.7.

^{19}F NMR (376 MHz, $CDCl_3$) δ -70.46 (dd, J = 26.5, 6.9 Hz, 1F), -132.66 (dd, J = 27.0, 7.6 Hz, 1F).

HRMS (+p APCI) calculated for $C_8H_5F_2N$ (M+H) 154.0470, found 154.0464

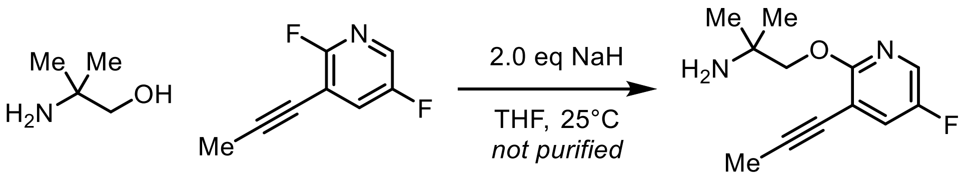

1-((5-fluoro-3-(prop-1-yn-1-yl)pyridin-2-yl)oxy)-2-methylpropan-2-amine (SI-12)

To a flame-dried 50 mL RBF equipped with a stir bar was added 2-amino-2-methyl-1-propanol (2.0 eq, 10.0 mmol, 891 mg) and NaH (2.0 eq, 10.0 mmol, 400 mg, 60 wt% in mineral oil). The solids were dissolved in THF (0.5 M to alcohol, 20 mL, SPS grade) and stirred at 25°C for 30 min. To this, a solution of crude **SI-11** (1.0 eq, 5.0 mmol, 765 mg) in THF (1.67 M to fluoride, 3.0 mL, SPS grade) was added to the reaction mixture. The resulting solution was stirred at 25°C for 2 h. Upon completion, the reaction mixture was added dropwise to 5 mL of a sat. aq. NH_4Cl solution in a separatory funnel. The aqueous layer was washed twice with 20 mL of EtOAc. The combined organic phases were washed with 20 mL of brine, dried over anhydrous Na_2SO_4, and concentrated *in vacuo*. The crude material was then re-dissolved in 10 mL of MeCN and washed three times with 10 mL of Hex. The combined MeCN phases were concentrated *in vacuo* to yield **SI-12** as a yellow oil which was used in the next step without further purification.

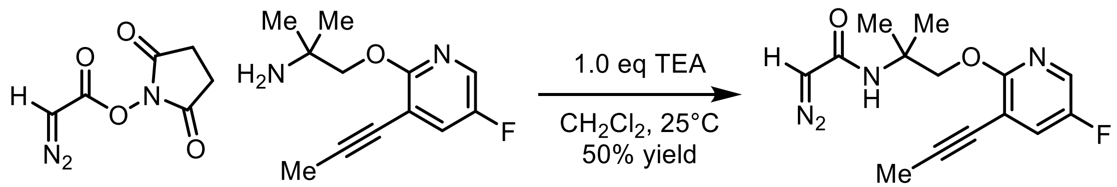

2-Diazo-N-(1-((5-fluoro-3-(prop-1-yn-1-yl)pyridin-2-yl)oxy)-2-methylpropan-2-yl)acetamide (SI-13)

Synthesized according to **General Procedure A** from **SI-12** (1.2 eq, 1.7 mmol, 377 mg). The crude material was purified via silica gel flash column chromatography eluting 25% EtOAc in Hex to yield **SI-13** (207 mg, 0.71 mmol) as a sticky yellow gum in 50% yield.

^{1}H NMR (400 MHz, $CDCl_3$) δ 7.86 (d, *J* = 3.0 Hz, 1H), 7.38 (dd, *J* = 8.0, 3.0 Hz, 1H), 5.60 (s, 1H), 4.61 (s, 1H), 4.31 (s, 2H), 2.10 (s, 3H), 1.50 (s, 6H).

^{13}C NMR (101 MHz, $CDCl_3$) δ 165.1, 159.8, 154.8 (d, *J* = 247.0 Hz), 132.1 (d, *J* = 26.0 Hz), 129.1 (d, *J* = 21.9 Hz), 109.4 (d, *J* = 6.1 Hz), 93.0, 73.4 (d, *J* = 1.9 Hz), 73.2, 54.7, 47.7, 24.3, 4.7.

^{19}F NMR (376 MHz, $CDCl_3$) δ -138.55 (d, *J* = 8.1 Hz, 1F).

HRMS (+p ESI) calculated for $C_{14}H_{15}FN_4O_2Na$ (M+Na) 313.1076, found 313.1078

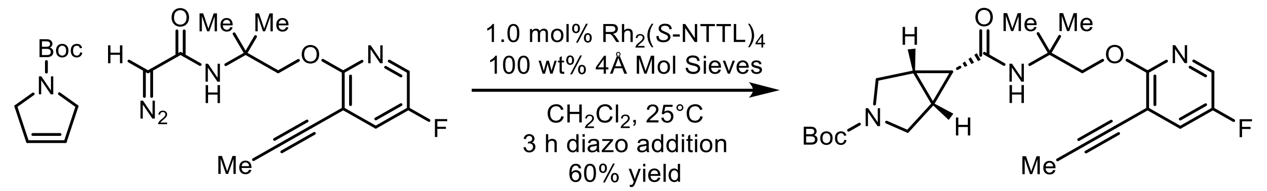

***tert*-Butyl *endo*-6-((1-((5-fluoro-3-(prop-1-yn-1-yl)pyridin-2-yl)oxy)-2-methylpropan-2-yl)carbamoyl)-3-azabicyclo[3.1.0]hexane-3-carboxylate (*endo*-19)**

Synthesized according to **General Procedure B** from **SI-13** (1.0 eq, 0.25 mmol, 72.6 mg). The crude material was purified via silica gel flash column chromatography eluting 60% EtOAc in Hex to yield ***endo*-19** (64.3 mg, 0.15 mmol) as a white solid in 60% yield.

^{1}H NMR (400 MHz, CDCl$_3$) δ 7.85 (d, J = 3.0 Hz, 1H), 7.38 (dd, J = 8.1, 3.0 Hz, 1H), 6.13 (s, 1H), 4.28 (d, J = 8.2 Hz, 2H), 3.81 (d, J = 11.0 Hz, 2H), 3.42 (d, J = 11.2 Hz, 1H), 3.33 (d, J = 9.6 Hz, 1H), 2.11 (s, 3H), 1.73 – 1.65 (m, 3H), 1.43 (d, J = 20.5 Hz, 6H), 1.40 (s, 9H).

^{13}C NMR (101 MHz, CDCl$_3$) δ 166.6, 159.9, 154.8 (d, J = 247.0 Hz), 154.2, 131.9 (d, J = 25.9 Hz), 129.2 (d, J = 21.9 Hz), 109.4 (d, J = 6.1 Hz), 93.0, 79.5, 73.5 (d, J = 1.8 Hz), 73.2, 54.1, 45.4, 28.6, 24.3, 23.6 (d, J = 57.6 Hz), 20.5 (d, J = 68.1 Hz), 4.8.

^{19}F NMR (376 MHz, CDCl$_3$) δ -138.77 (d, J = 8.0 Hz, 1F).

HRMS (+p APCI) calculated for $C_{23}H_{30}FN_3O_4$ (M+H) 432.2300, found 432.2298

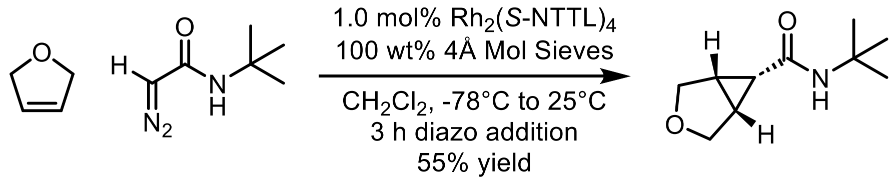

***endo*-N-(*tert*-Butyl)-3-oxabicyclo[3.1.0]hexane-6-carboxamide (*endo*-21)**

Purification of trap: 2,5-dihydrofuran from commercial suppliers was first filtered through a plug of silica before use.

To a flame-dried 4 mL vial charged with a stir bar and activated 4 Å Mol Sieves (100 wt%, 250 mg) was added 2,5-dihydrofuran (10 eq, 2.5 mmol, 175 mg), Rh_2(*S*-NTTL)$_4$ (1 mol%, 2.5 µmol, 3.6 mg), and CH_2Cl_2 (0.25 M to diazo, 1.0 mL, distilled). In a separate flame-dried 4 mL vial was added **20** (1.0 eq, 0.25 mmol, 35.3 mg) and CH_2Cl_2 (0.25 M to diazo, 1.0 mL, distilled). Both vials were sealed with a septa-lined cap and sparged with argon for 5 min. The solution of catalyst and trap was then cooled to -78°C, to which the diazo solution was then added dropwise via dual syringe pump over 3 h [settings: 1 mL syringe, diameter 4.71 mm; Air-Tite/SilverPoint 22 G x 4" long hypodermic needle], allowing the reaction to naturally warm to 25°C without replenishing the cooling bath. After the elapsed time, the reaction solution was filtered through a plug of celite, washing with CH_2Cl_2, and the filtrate concentrated *in vacuo*. The crude material was subjected to a Kugelrohr distillation at 0.16 bar (120 torr) and 110°C for at least 1 h to distill off the unreacted trap. The crude residue after Kugelrohr distillation was then purified via silica gel flash column chromatography eluting 60% EtOAc in Hex to yield ***endo*-21** (25.3 mg, 0.14 mmol) as a white solid in 55% yield.

^{1}H NMR (400 MHz, $CDCl_3$) δ 5.67 (s, 1H), 4.06 (d, *J* = 8.8 Hz, 2H), 3.81 (ddd, *J* = 8.8, 1.9, 1.2 Hz, 2H), 1.87 – 1.82 (m, 2H), 1.66 (dd, *J* = 8.6, 7.7 Hz, 1H), 1.31 (s, 9H).

^{13}C NMR (101 MHz, $CDCl_3$) δ 167.6, 67.7, 51.2, 28.9, 24.7, 22.4.

HRMS (+p APCI) calculated for $C_{10}H_{18}NO_2$ (M+H) 184.1339, found 184.1335

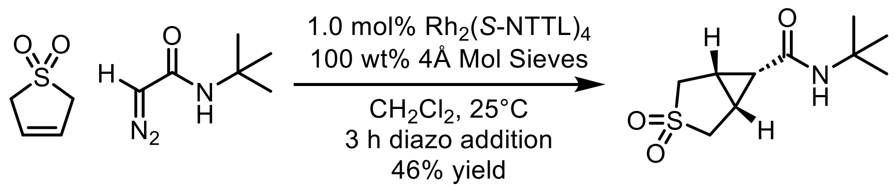

***endo*-N-(*tert*-Butyl)-3-thiabicyclo[3.1.0]hexane-6-carboxamide 3,3-dioxide (*endo*-22)**

Synthesized according to a modified **General Procedure B** from **20** (1.0 eq, 0.25 mmol, 35.3 mg) and sulfolene (10.0 eq, 2.5 mmol, 295 mg) instead of **5**. The crude material was purified via silica gel flash column chromatography eluting 60% EtOAc in Hex to yield ***endo*-22** (26.6 mg, 0.12 mmol) as a white solid in 46% yield.

^{1}H NMR (400 MHz, $CDCl_3$) δ 5.57 (s, 1H), 3.59 (dp, J = 13.3, 2.5 Hz, 2H), 3.18 (dddd, J = 13.5, 6.6, 2.6, 1.3 Hz, 2H), 2.17 – 2.05 (m, 2H), 1.65 (t, J = 8.0 Hz, 1H), 1.35 (s, 9H).

^{13}C NMR (101 MHz, $CDCl_3$) δ 167.8, 52.1, 47.9, 29.0, 23.5, 17.1.

HRMS (+p APCI) calculated for $C_{10}H_{18}NO_3S$ (M+H) 232.1009, found 232.1007

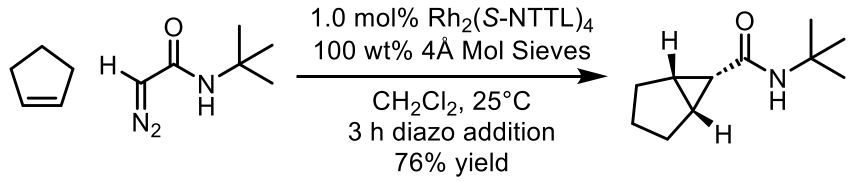

***endo*-N-(*tert*-Butyl)bicyclo[3.1.0]hexane-6-carboxamide (*endo*-23)**

Purification of cyclopentene: Cyclopentene from commercial suppliers was first filtered through a plug of silica before use.

Synthesized according to a modified **General Procedure B** from **20** (1.0 eq, 0.25 mmol, 35.3 mg) and cyclopentene (10.0 eq, 2.5 mmol, 170 mg, 0.23 mL) instead of **5**. The crude material was purified via silica gel flash column chromatography eluting 40% EtOAc in Hex to yield ***endo*-23** (34.5 mg, 0.19 mmol) as a white solid in 76% yield.

^{1}H NMR (400 MHz, $CDCl_3$) δ 5.32 (s, 1H), 1.93 (ddd, J = 13.2, 8.4, 1.2 Hz, 2H), 1.82 (dddd, J = 14.1, 12.8, 7.2, 3.3 Hz, 2H), 1.62 (dt, J = 13.5, 8.9 Hz, 1H), 1.54 – 1.44 (m, 3H), 1.33 (s, 9H), 1.12 (dddd, J = 19.3, 13.6, 8.4, 2.1 Hz, 1H).

^{13}C NMR (101 MHz, $CDCl_3$) δ 169.5, 51.0, 28.8, 26.3, 25.3, 22.7, 22.3.

HRMS (+p APCI) calculated for $C_{11}H_{20}NO$ (M+H) 182.1547, found 182.1541

6. Spectra

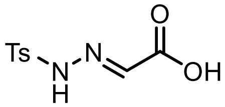

(*E*)-2-(2-tosylhydrazineylidene)acetic acid (SI-1)

^{1}H NMR (400 MHz, $CDCl_3$)

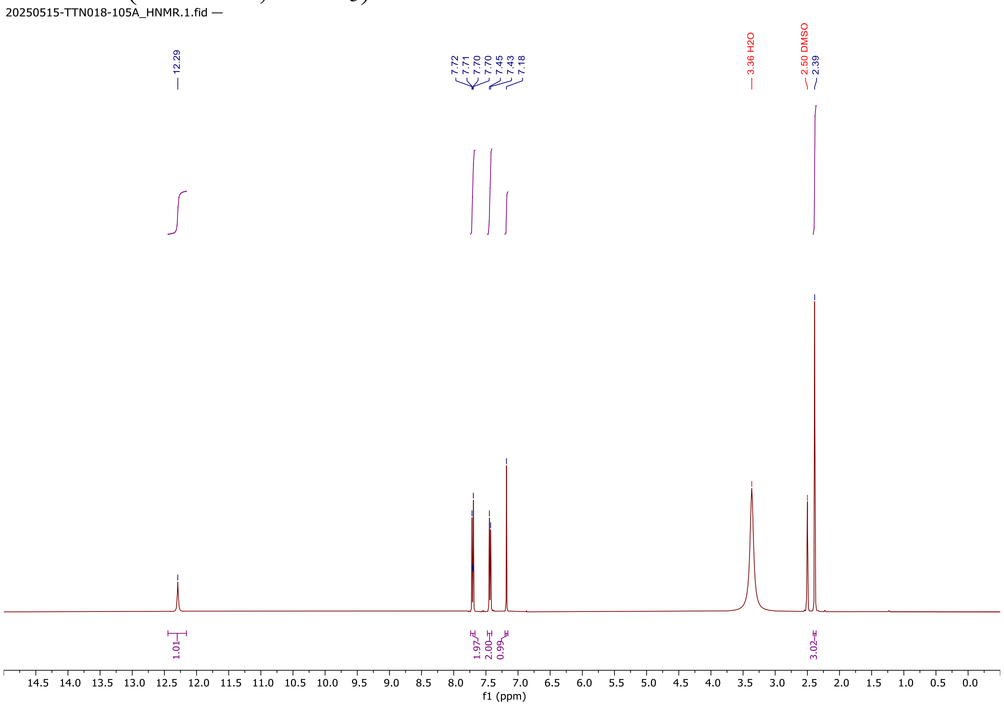

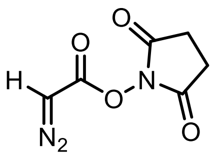

2,5-dioxopyrrolidin-1-yl 2-diazoacetate (24)

^{1}H NMR (400 MHz, $CDCl_3$)

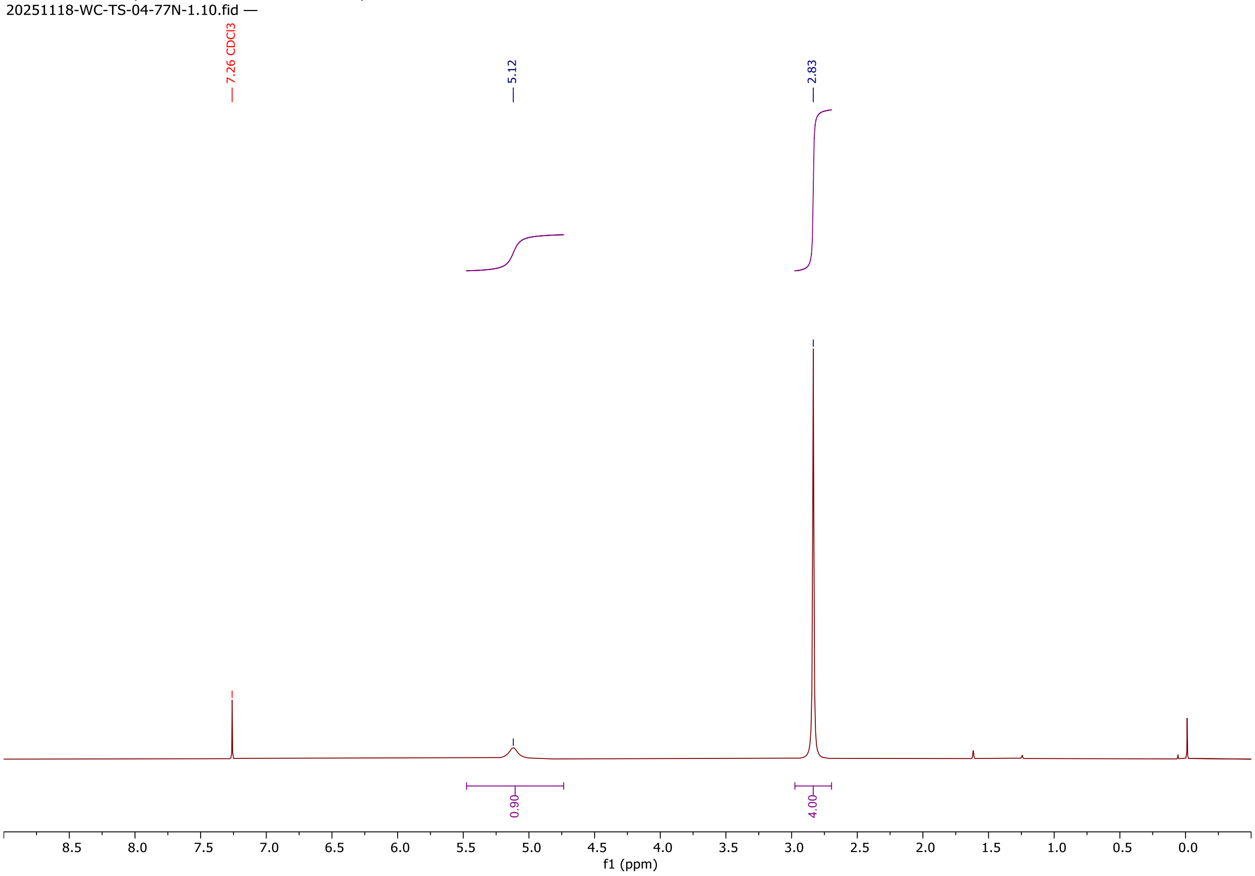

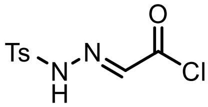

2-(2-Tosylhydrazineylidene)acetyl chloride (SI-2)

^{1}H NMR (400 MHz, $CDCl_3$)

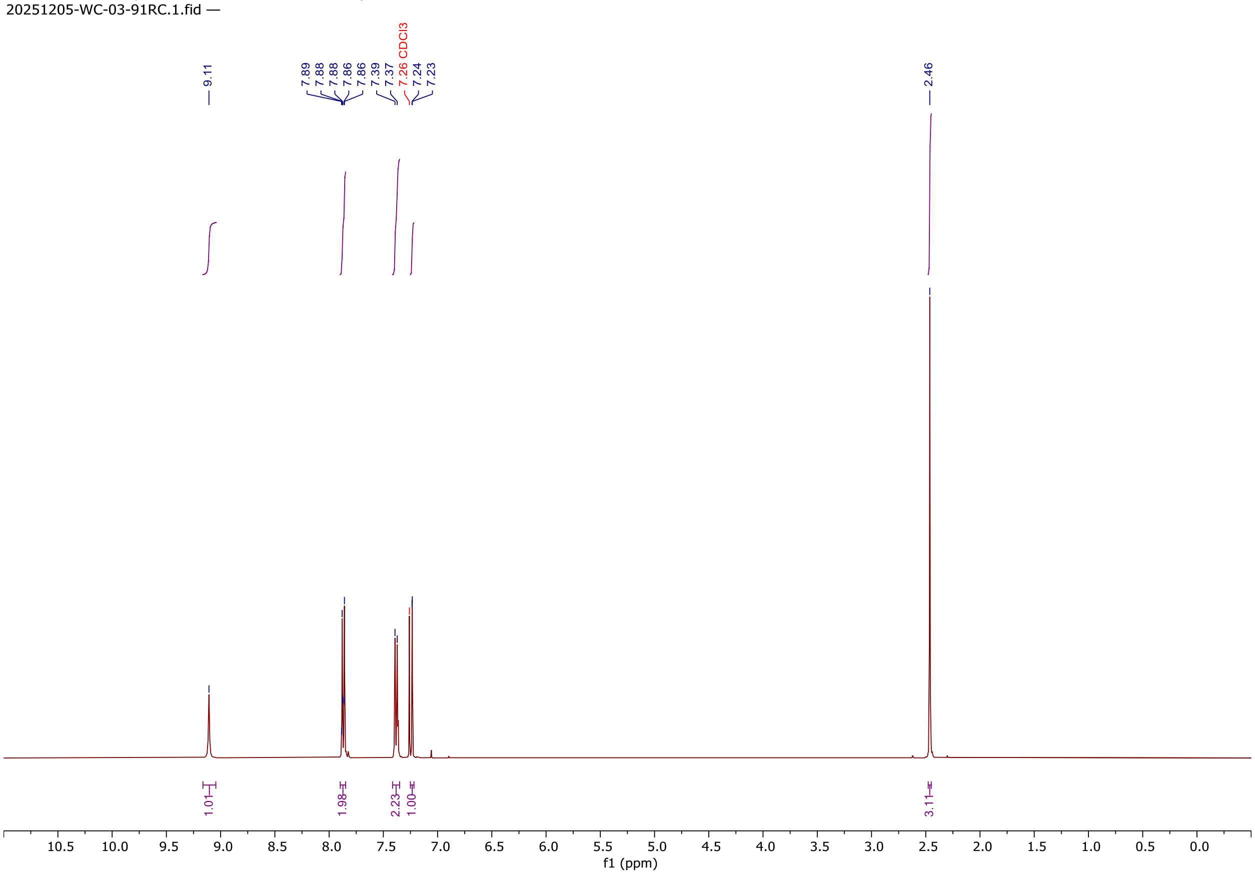

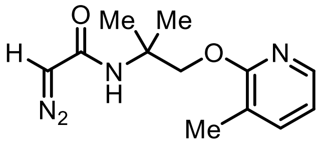

2-diazo-N-(2-methyl-1-((3-methylpyridin-2-yl)oxy)propan-2-yl)acetamide (8)

^{1}H NMR (400 MHz, $CDCl_3$)

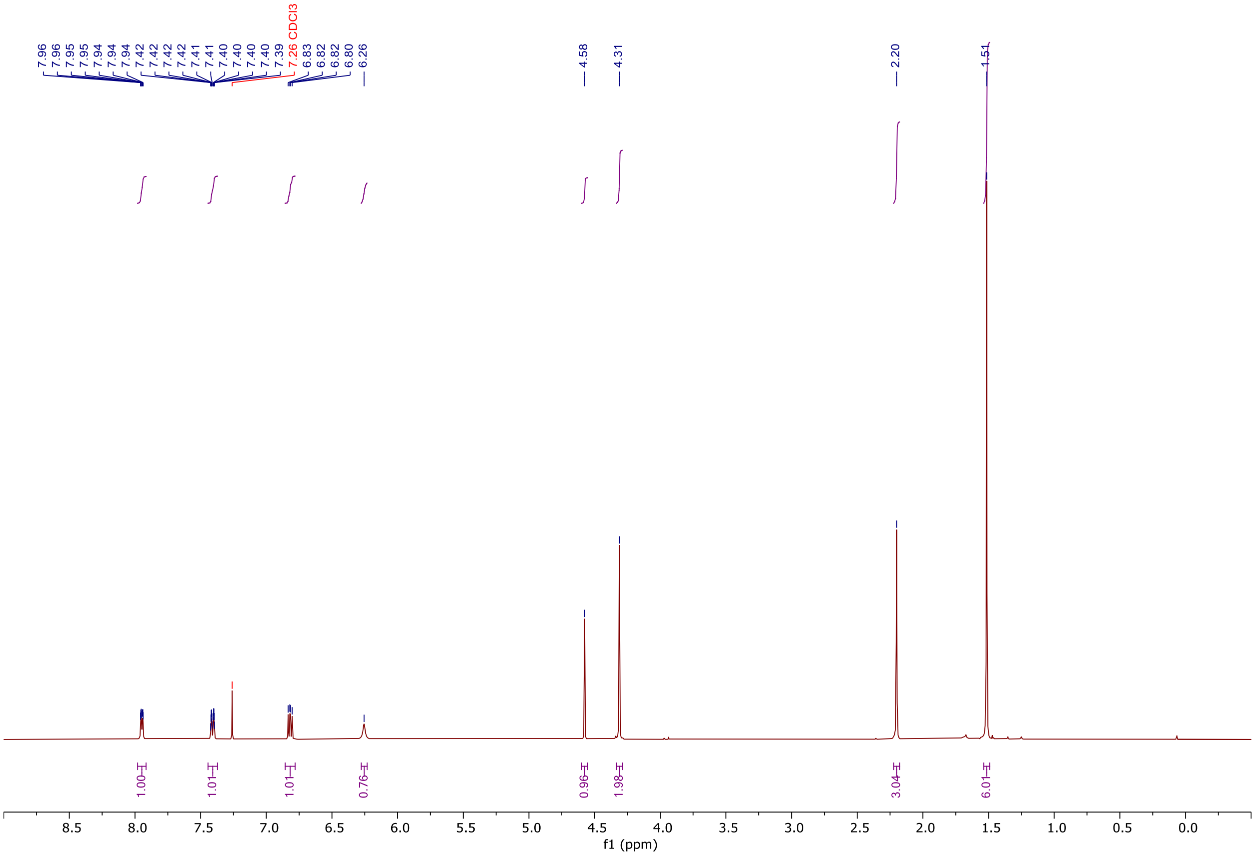

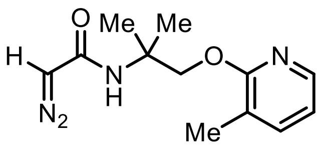

^{13}C NMR (101 MHz, $CDCl_3$)

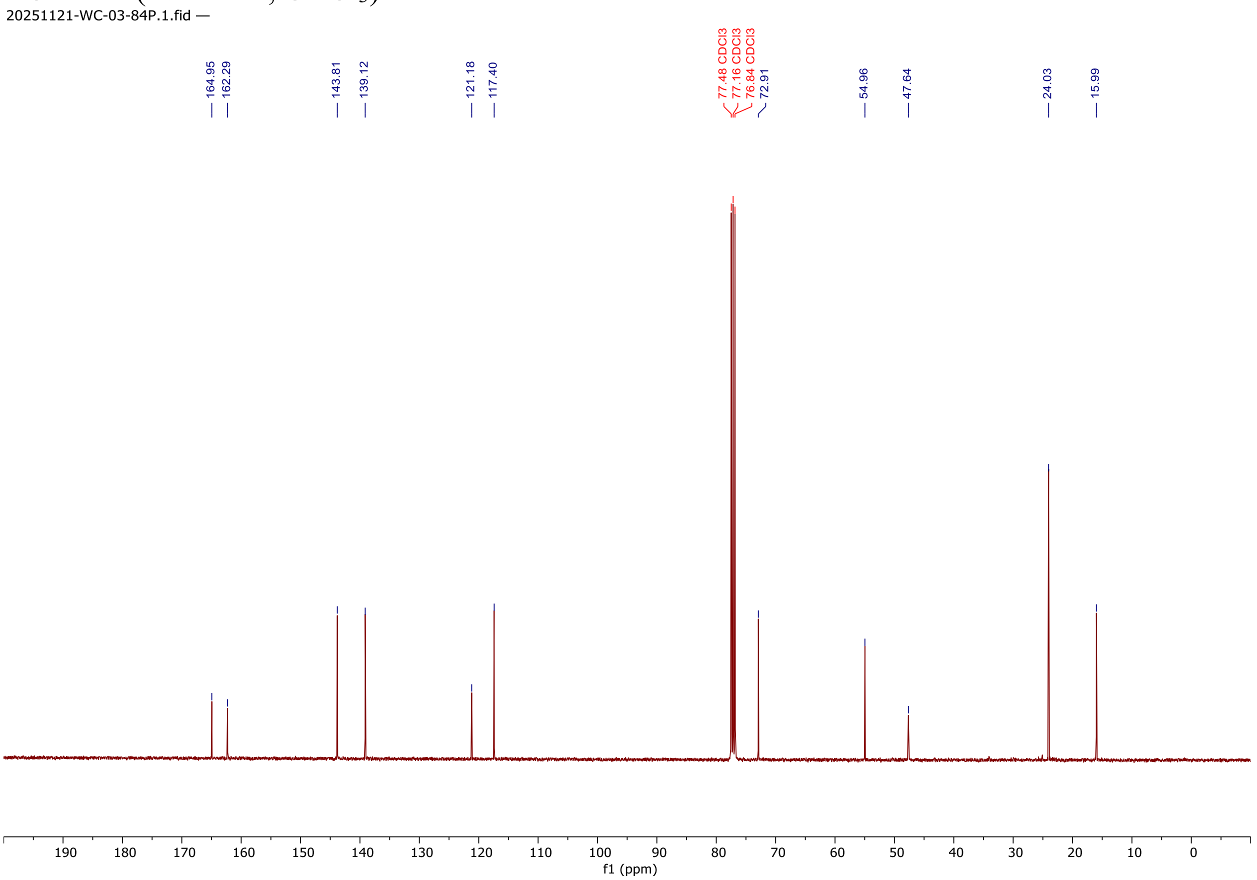

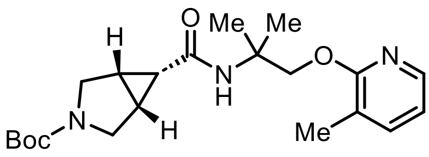

***endo-tert*-Butyl-6-((2-methyl-1-((3-methylpyridin-2-yl)oxy)propan-2-yl)carbamoyl)-3-azabicyclo[3.1.0]hexane-3-carboxylate (*endo*-9)**

^{1}H NMR (400 MHz, $CDCl_3$)

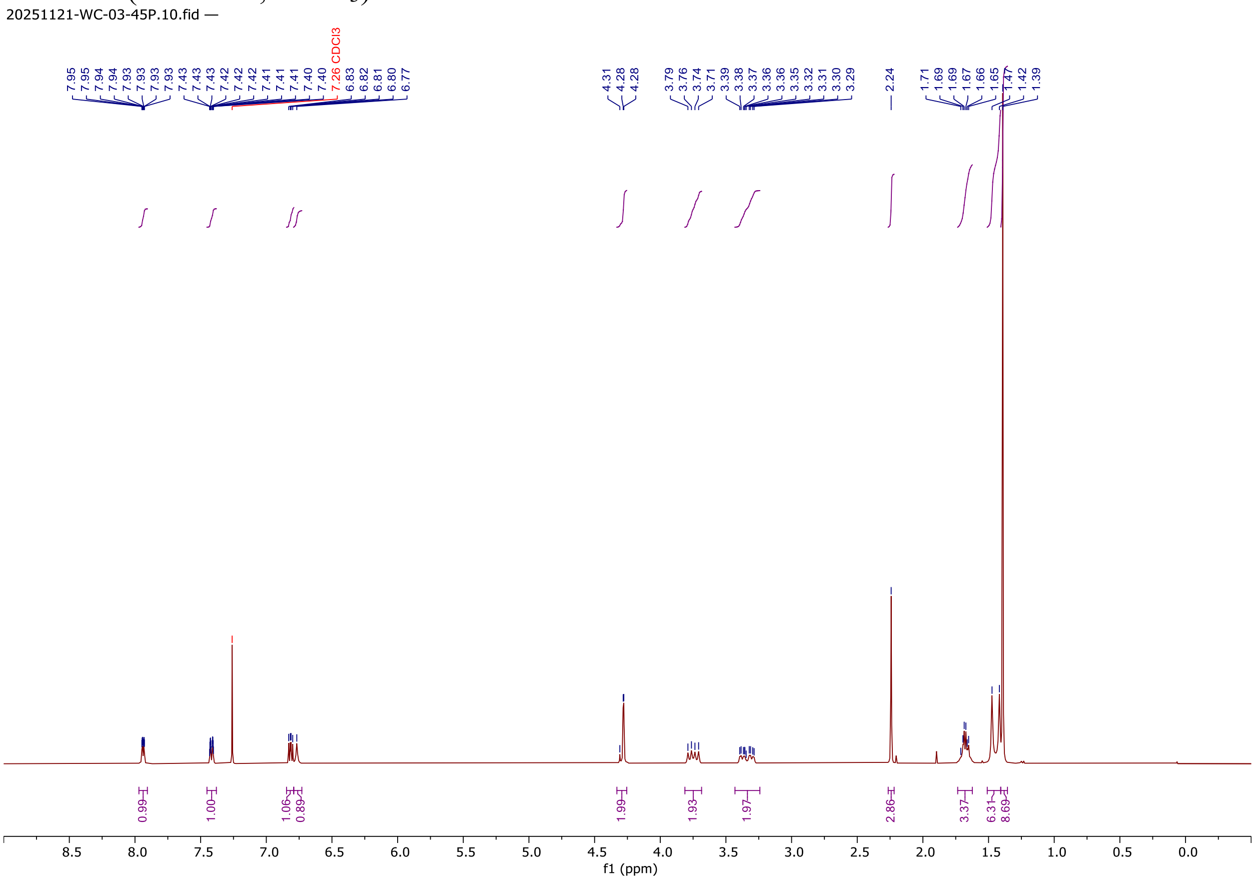

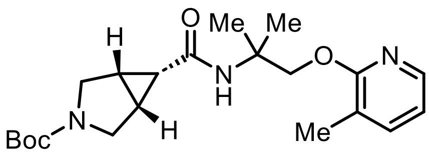

^{13}C NMR (101 MHz, $CDCl_3$)

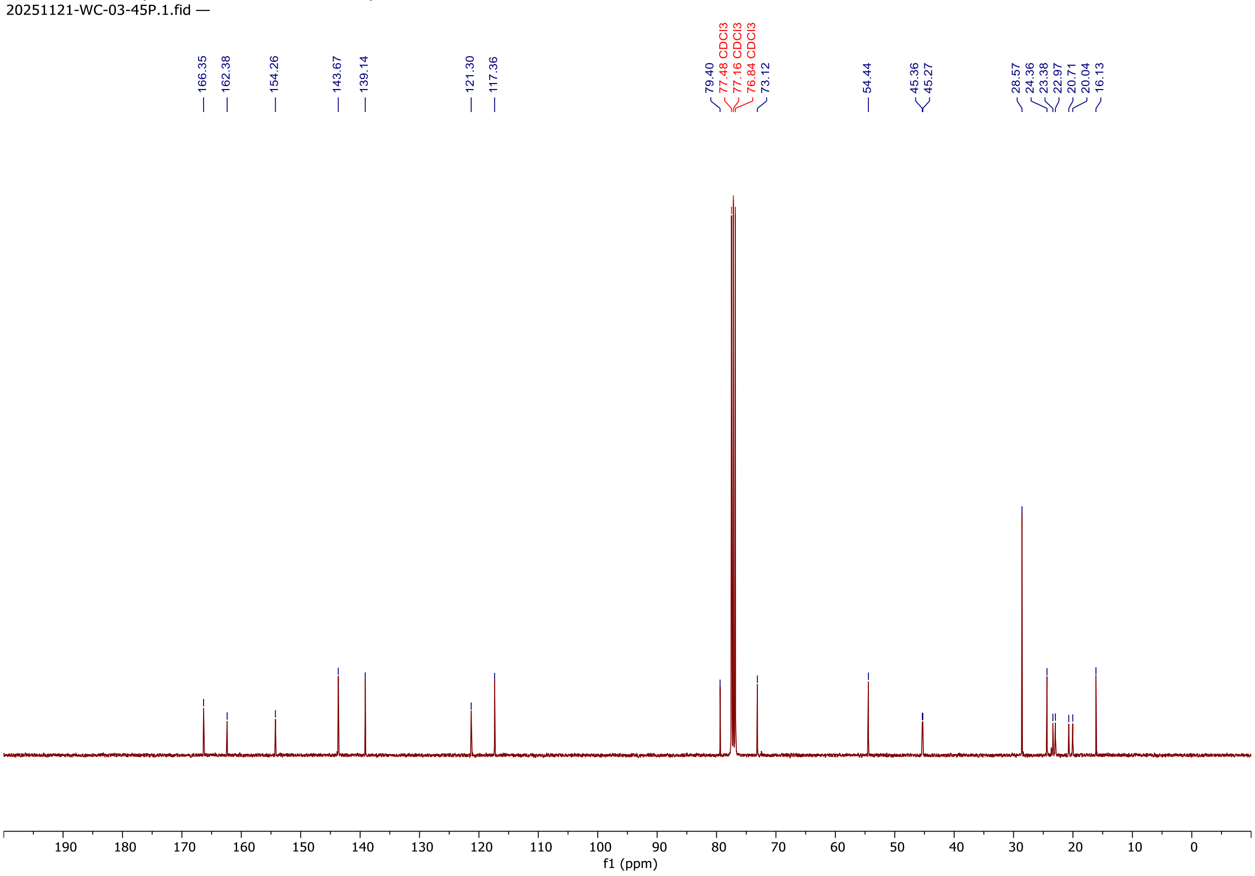

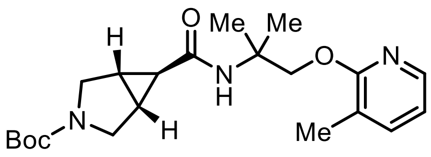

***exo-tert*-Butyl-6-((2-methyl-1-((3-methylpyridin-2-yl)oxy)propan-2-yl)carbamoyl)-3-azabicyclo[3.1.0]hexane-3-carboxylate (*exo*-9)**

^{1}H NMR (400 MHz, $CDCl_3$)

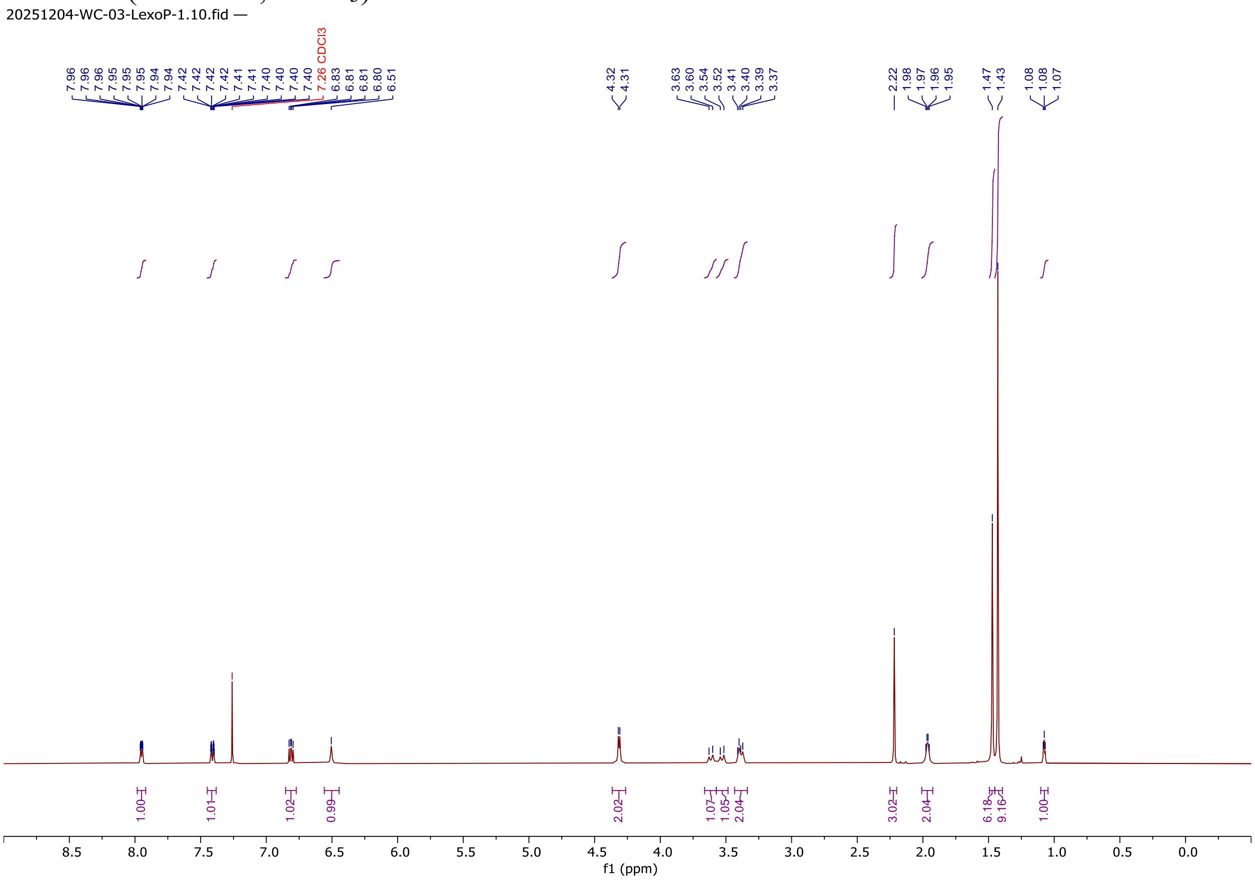

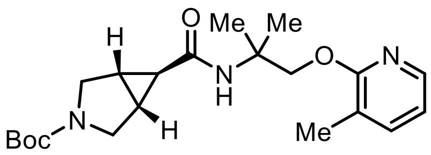

^{13}C NMR (101 MHz, $CDCl_3$)

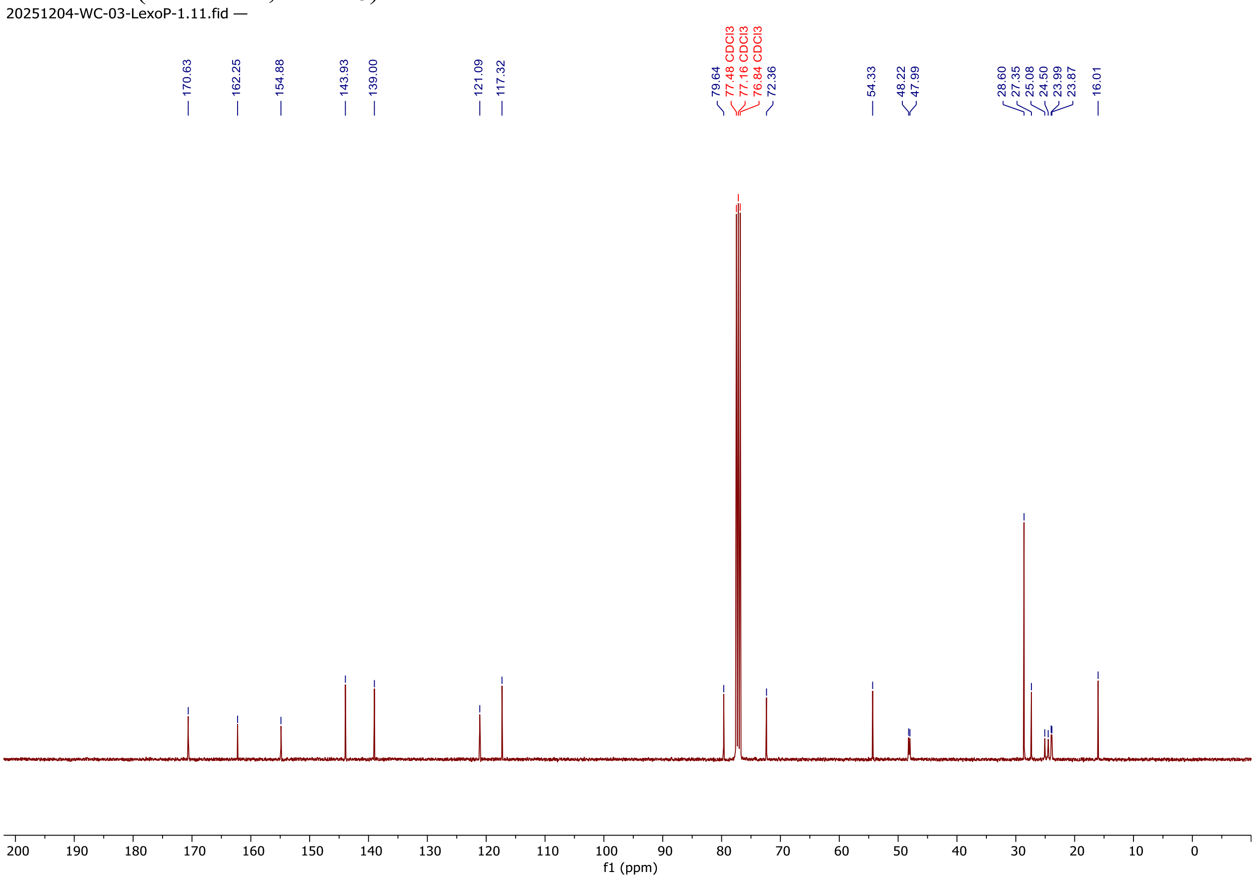

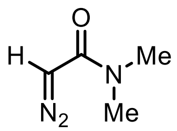

2-Diazo-N,N-dimethylacetamide (10)

^{1}H NMR (400 MHz, $CDCl_3$)

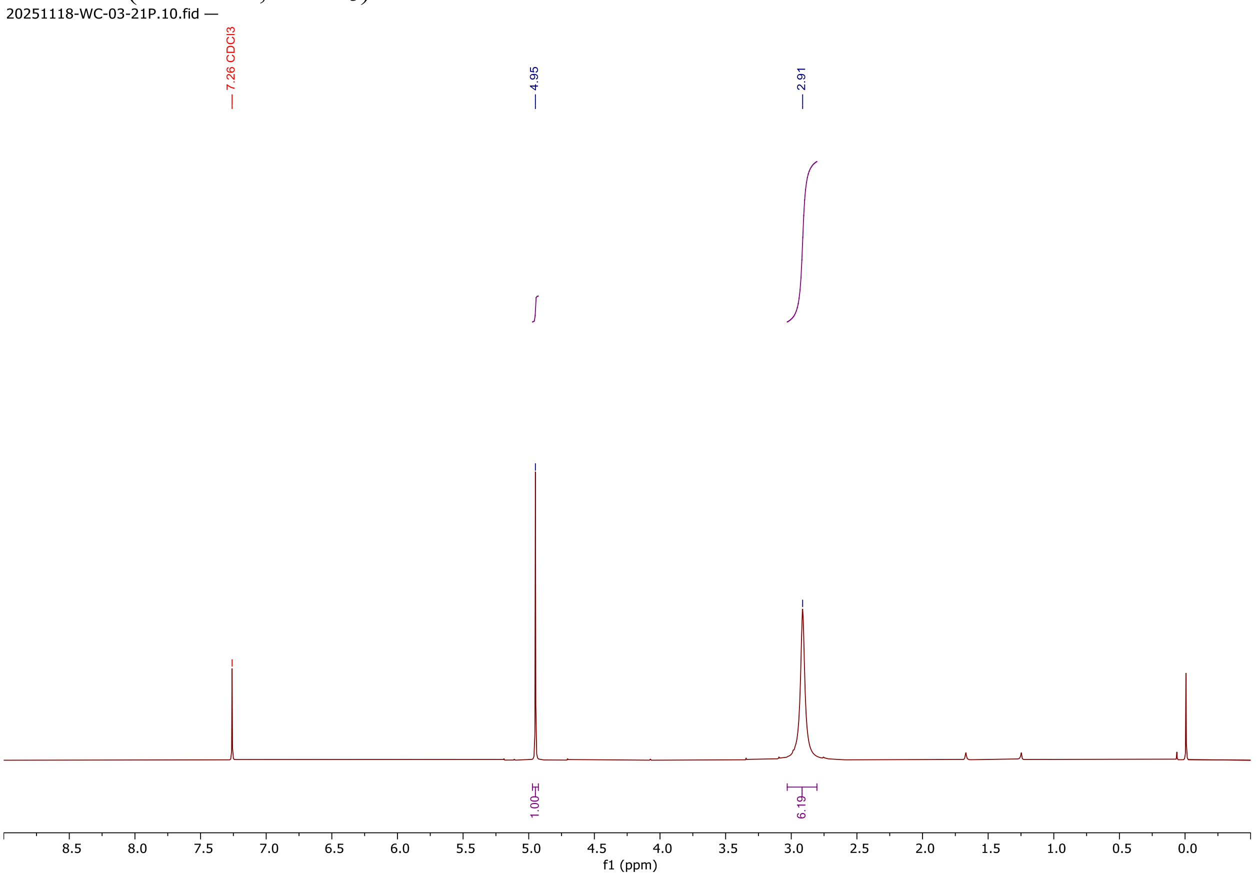

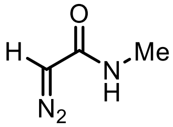

2-Diazo-N-methylacetamide (SI-3)

^{1}H NMR (400 MHz, $CDCl_3$)

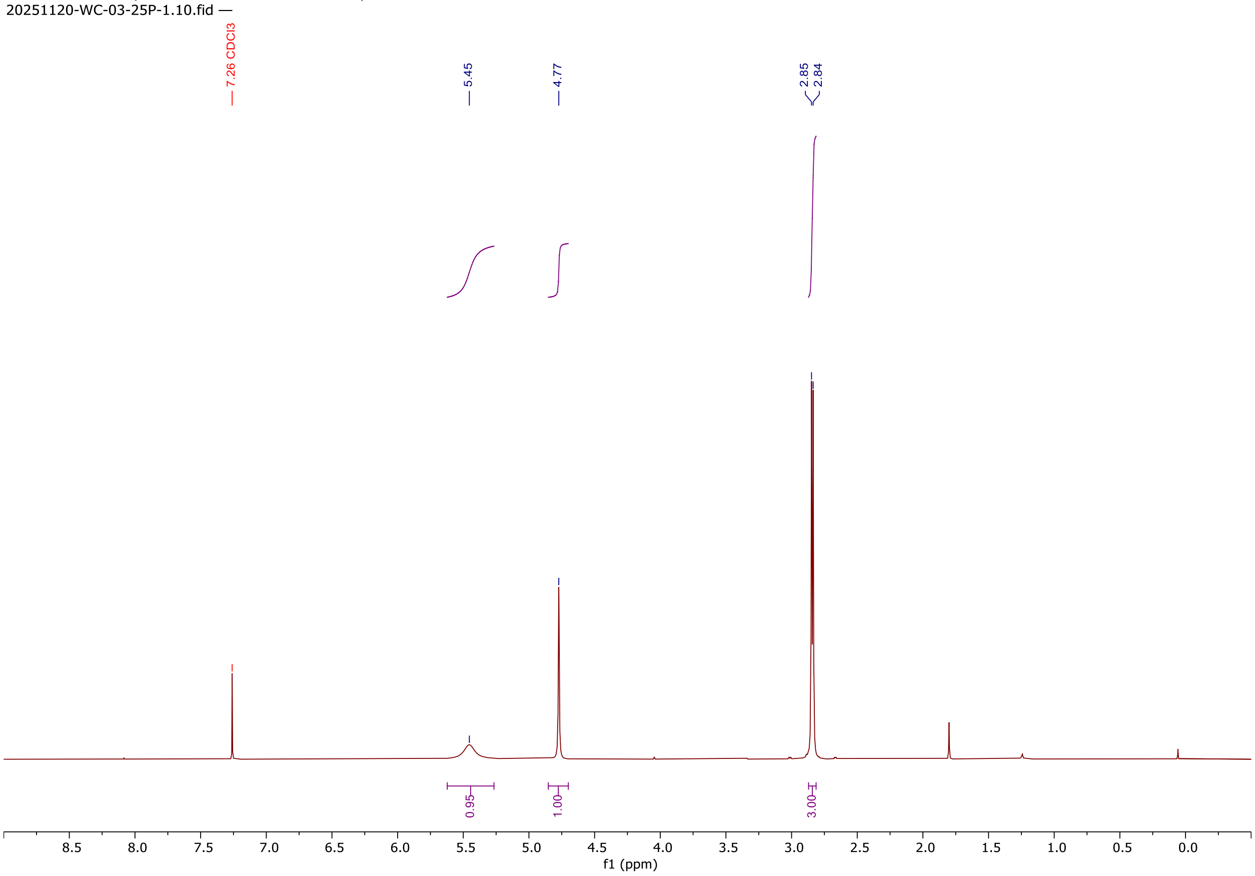

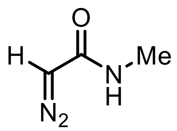

^{13}C NMR (101 MHz, $CDCl_3$)

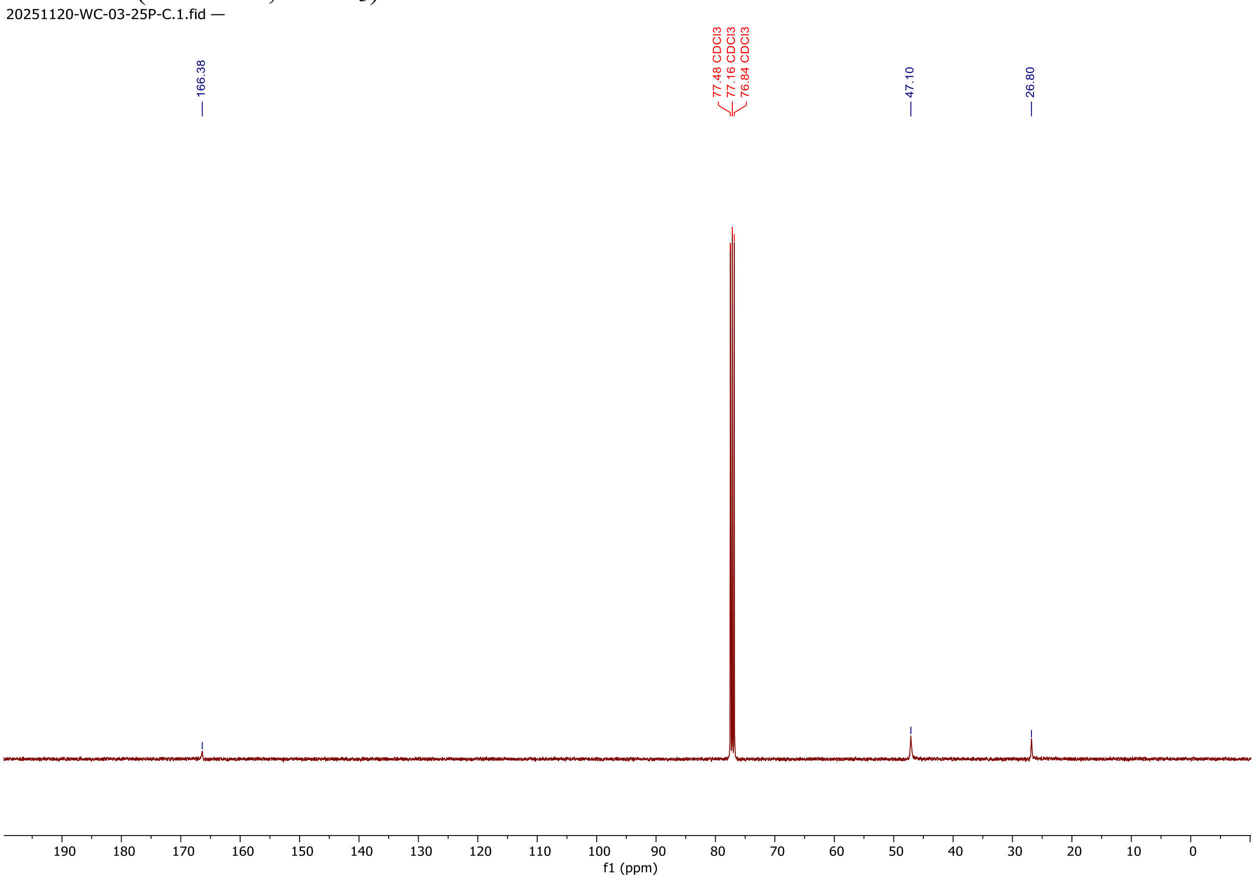

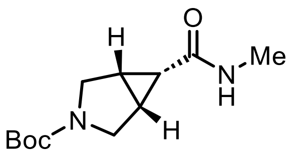

***tert*-Butyl *endo*-6-(methylcarbamoyl)-3-azabicyclo[3.1.0]hexane-3-carboxylate (*endo*-11)**

^{1}H NMR (400 MHz, $CDCl_3$)

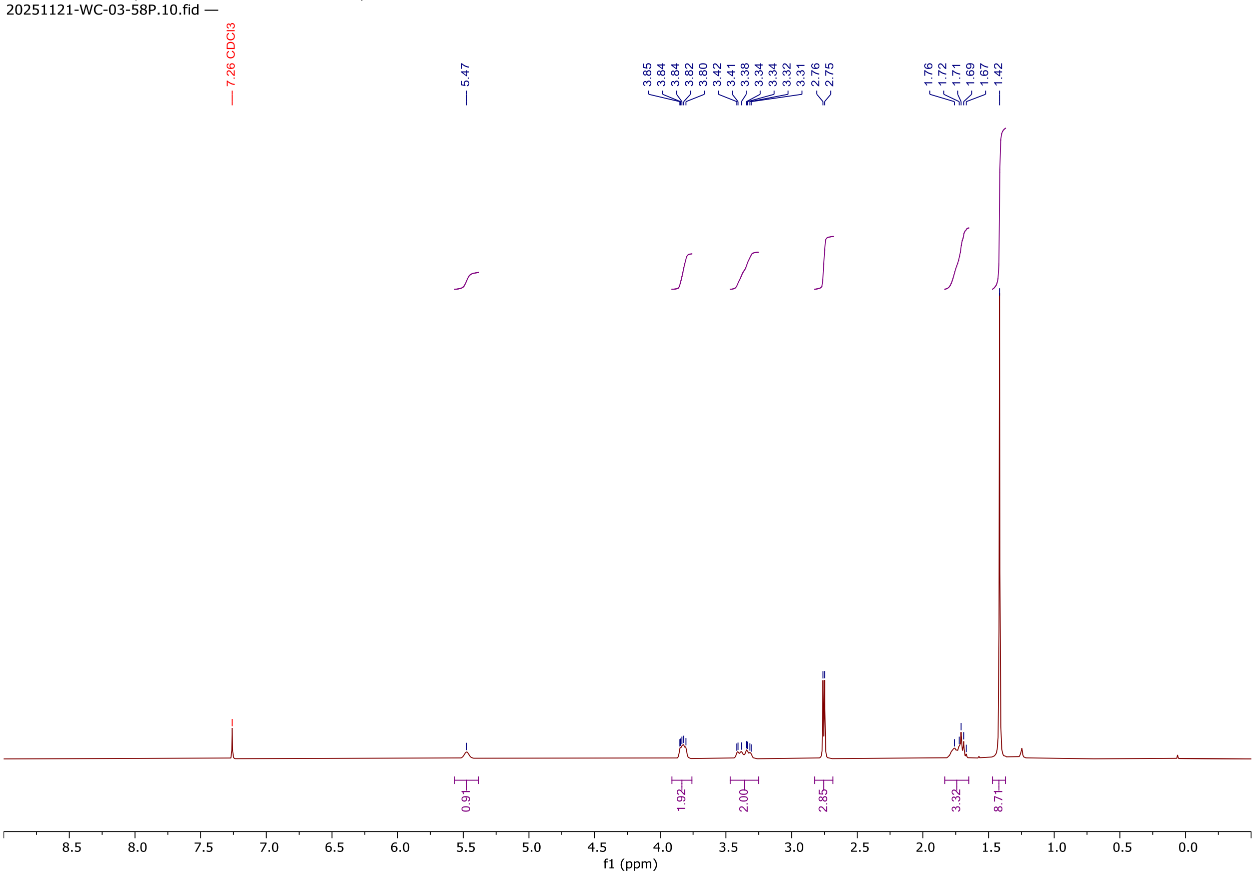

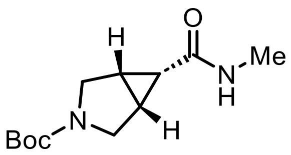

^{13}C NMR (101 MHz, $CDCl_3$)

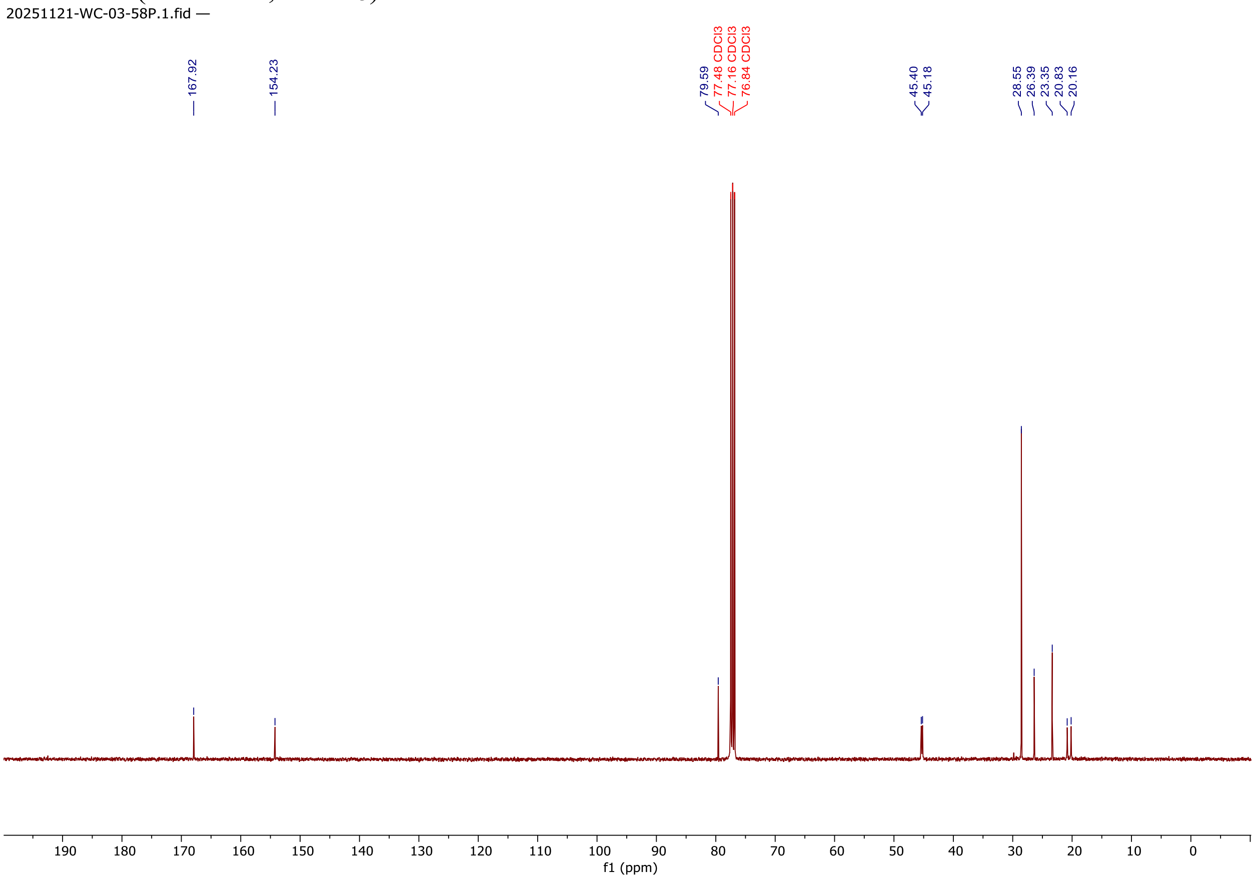

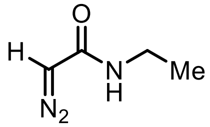

2-Diazo-N-ethylacetamide (SI-4)

^{1}H NMR (400 MHz, $CDCl_3$)

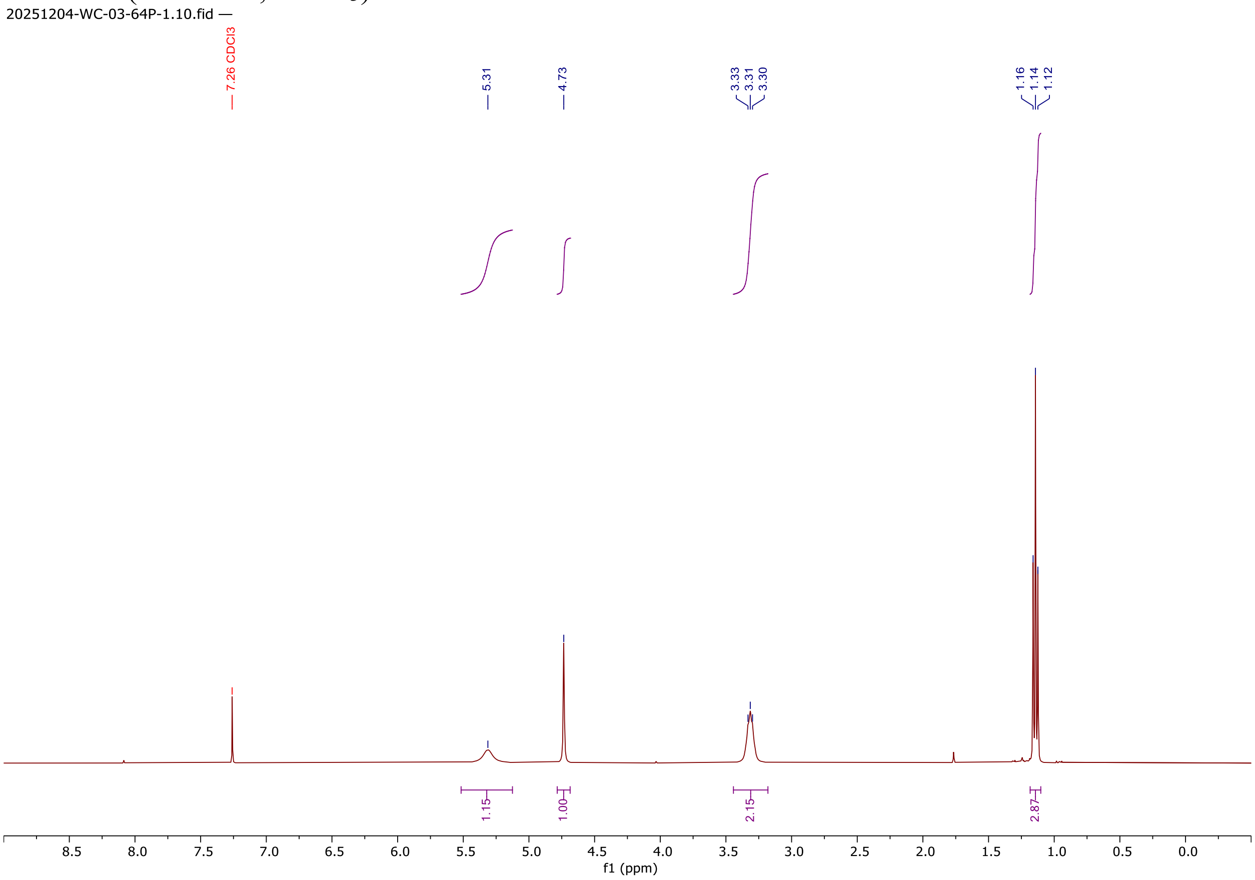

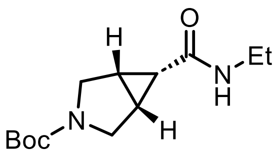

***tert*-Butyl *endo*-6-(ethylcarbamoyl)-3-azabicyclo[3.1.0]hexane-3-carboxylate (*endo*-12)**

^{1}H NMR (400 MHz, $CDCl_3$)

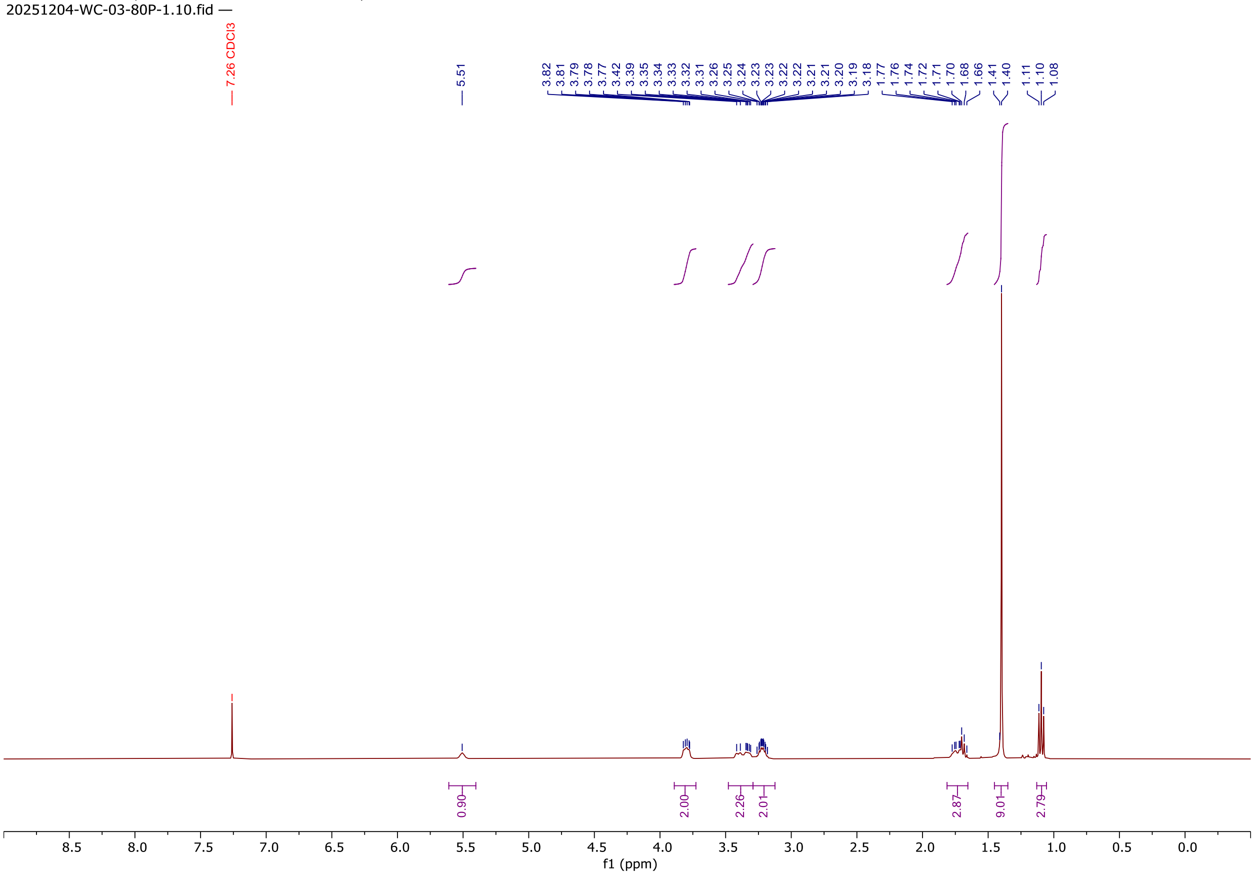

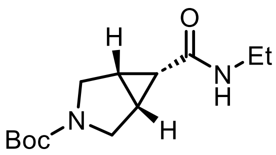

^{13}C NMR (101 MHz, $CDCl_3$)

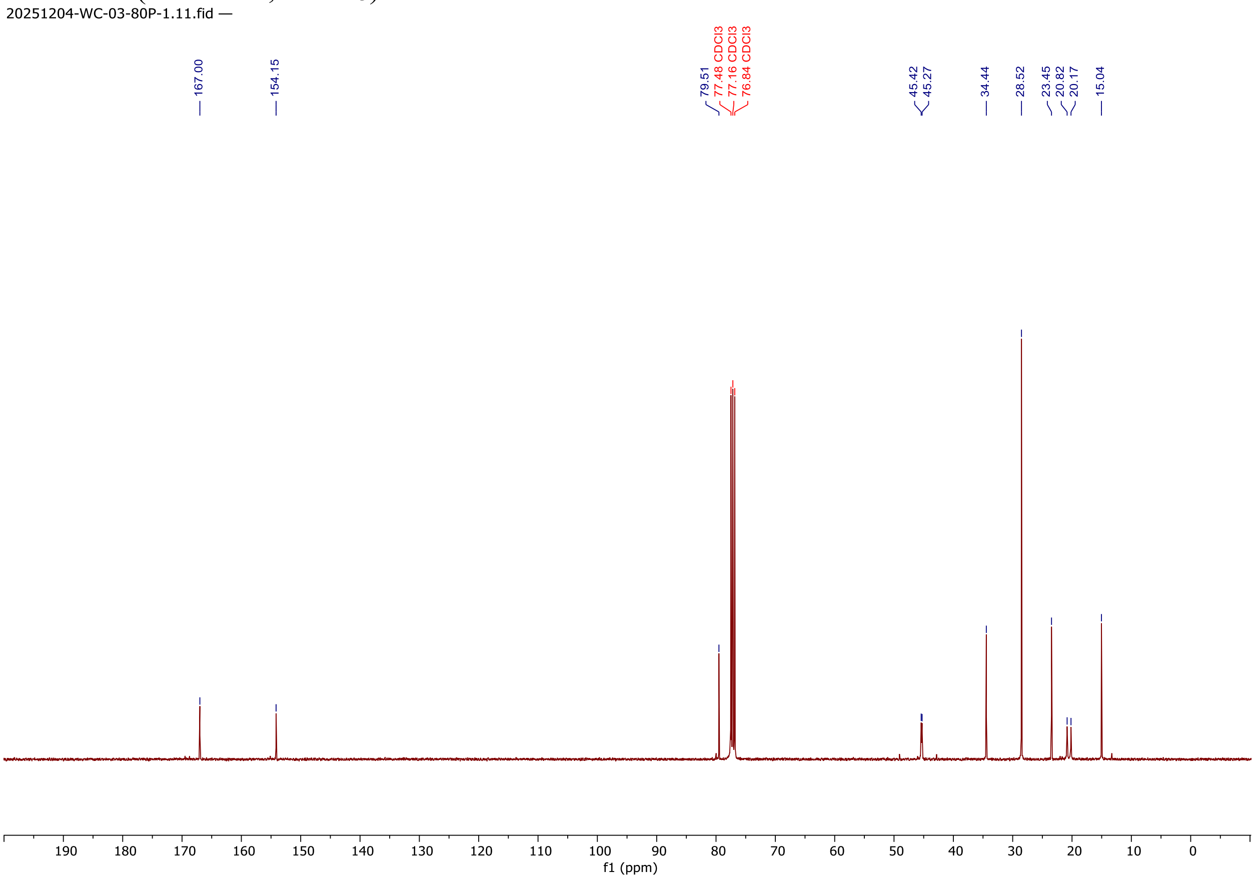

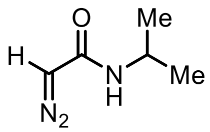

2-Diazo-N-isopropylacetamide (SI-5)

^{1}H NMR (400 MHz, $CDCl_3$)

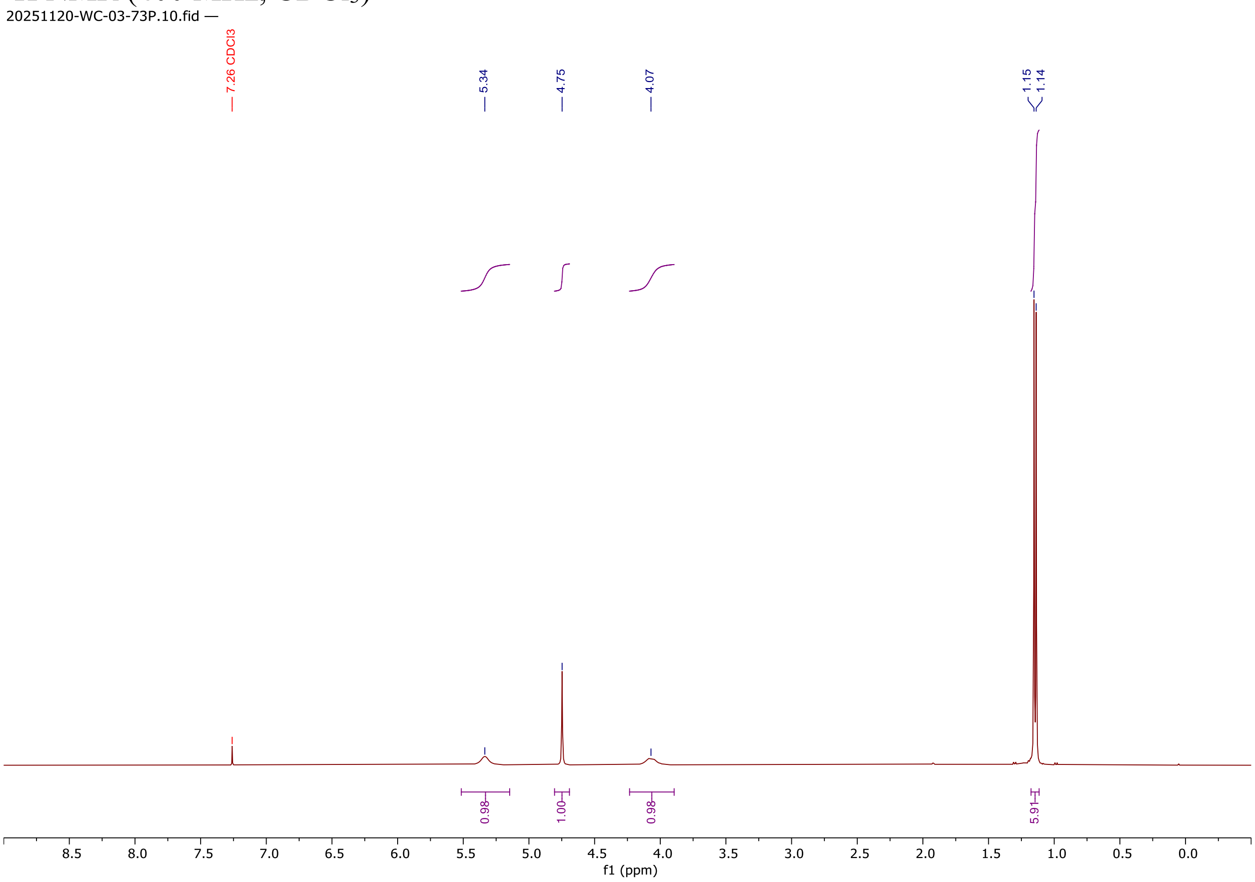

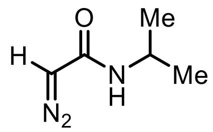

^{13}C NMR (101 MHz, $CDCl_3$)

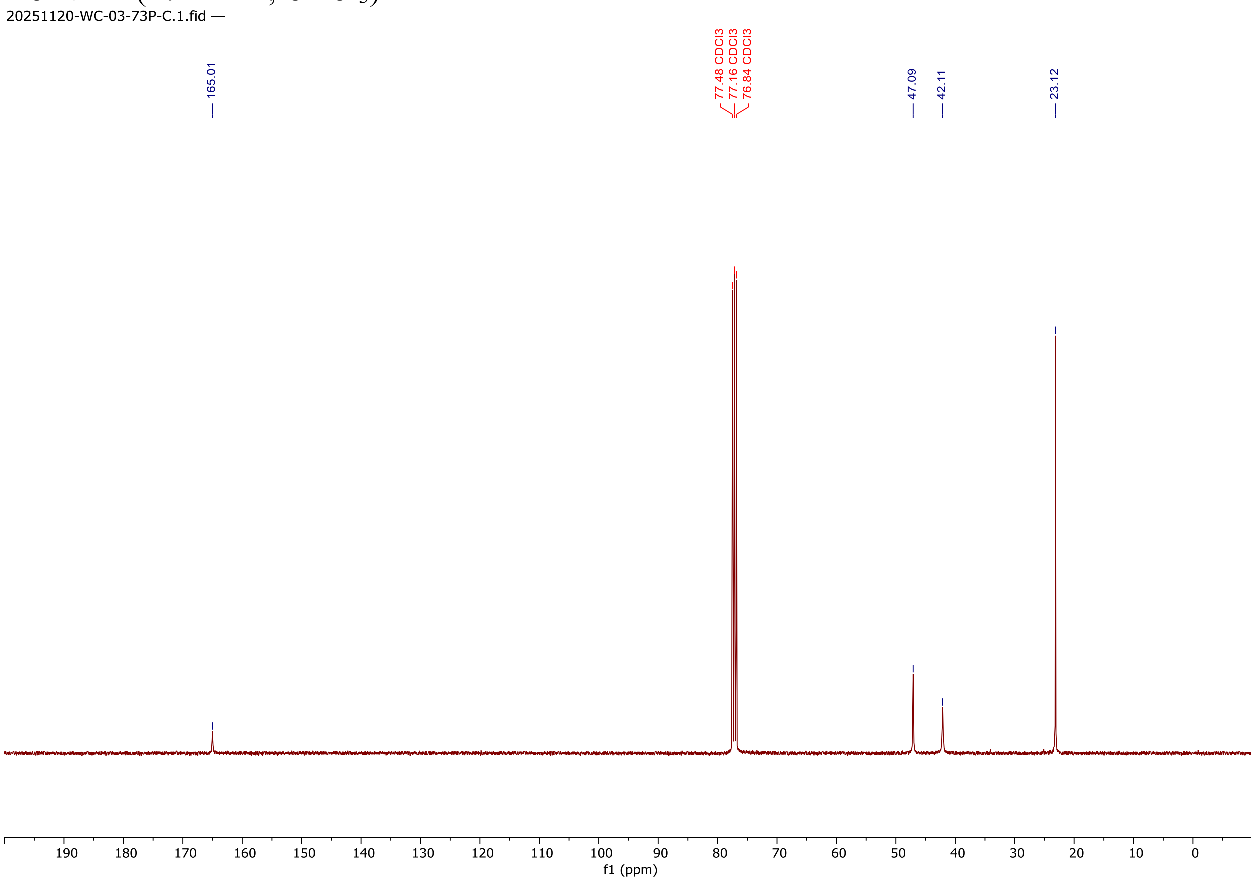

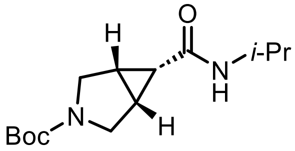

tert-Butyl endo-6-(isopropylcarbamoyl)-3-azabicyclo[3.1.0]hexane-3-carboxylate (endo-13)

^{1}H NMR (400 MHz, $CDCl_3$)

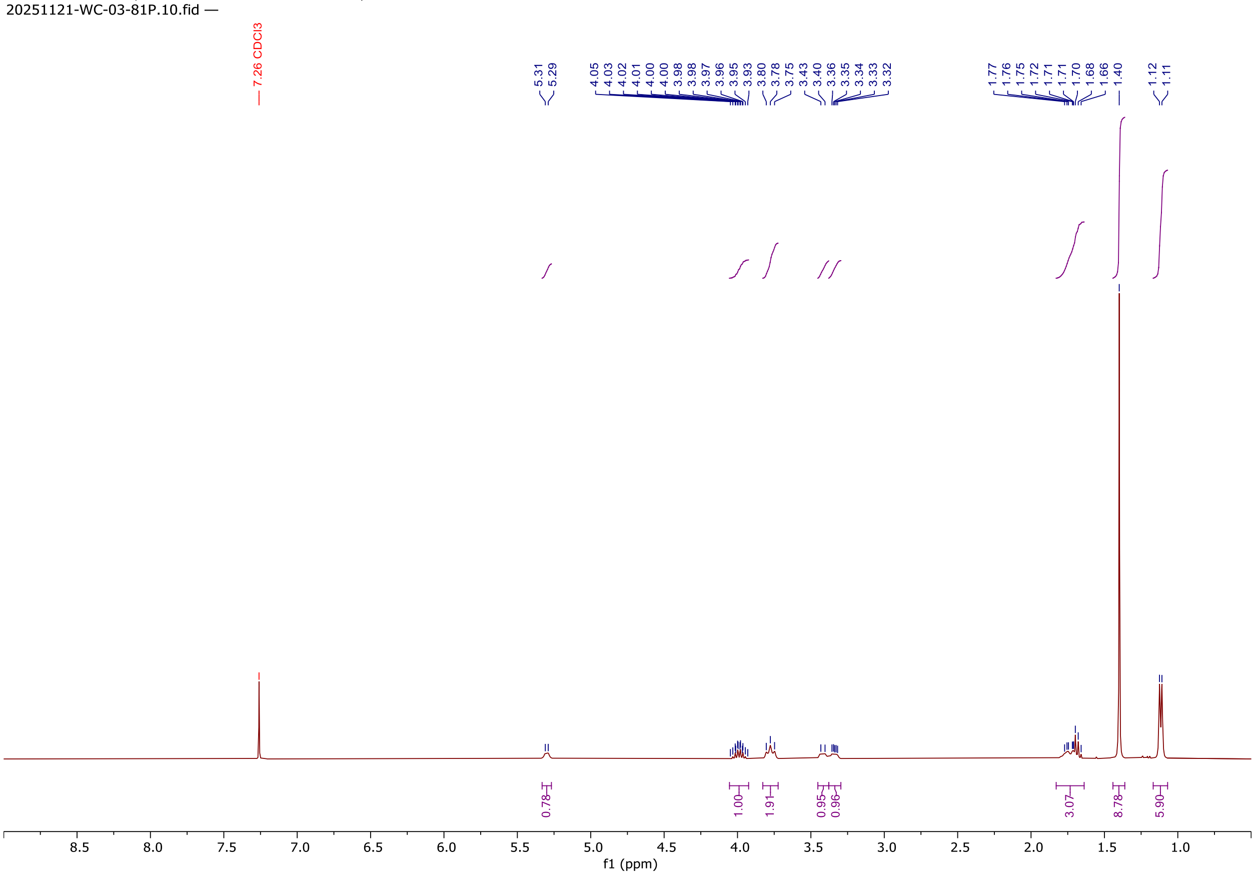

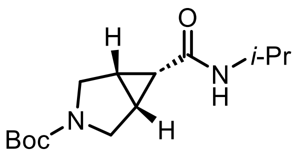

^{13}C NMR (101 MHz, $CDCl_3$)

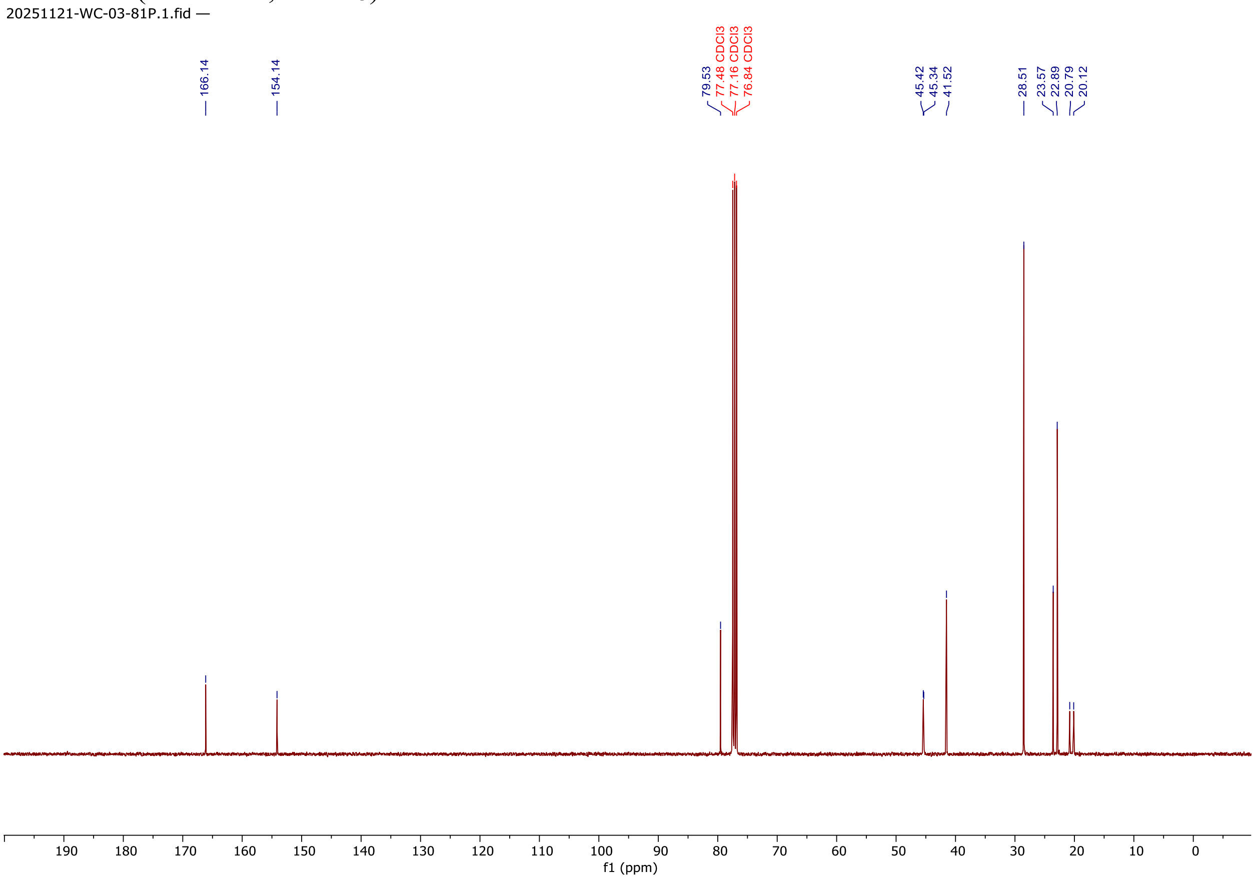

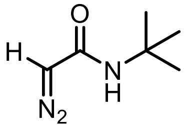

N-(*tert*-butyl)-2-diazoacetamide (20)

^{1}H NMR (400 MHz, $CDCl_3$)

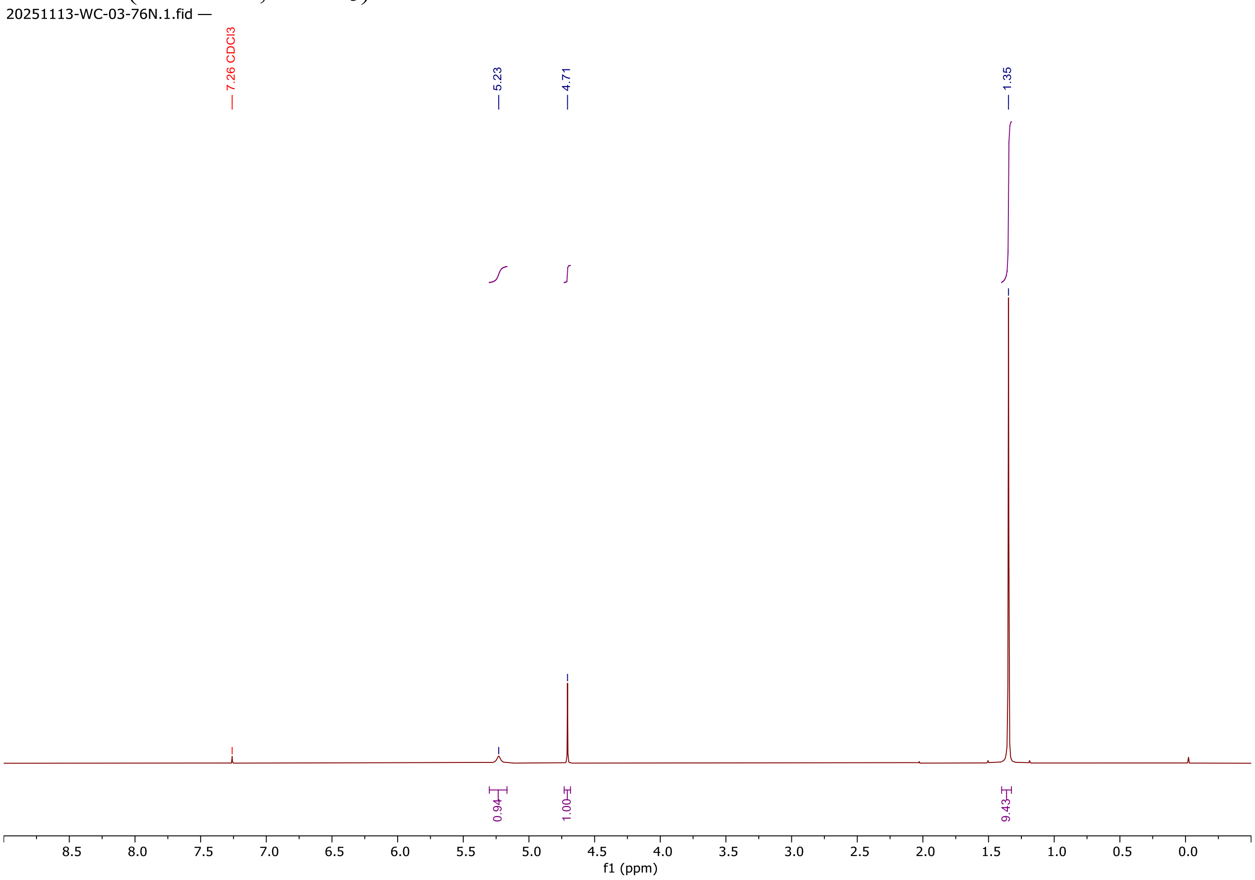

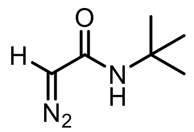

^{13}C NMR (101 MHz, $CDCl_3$)

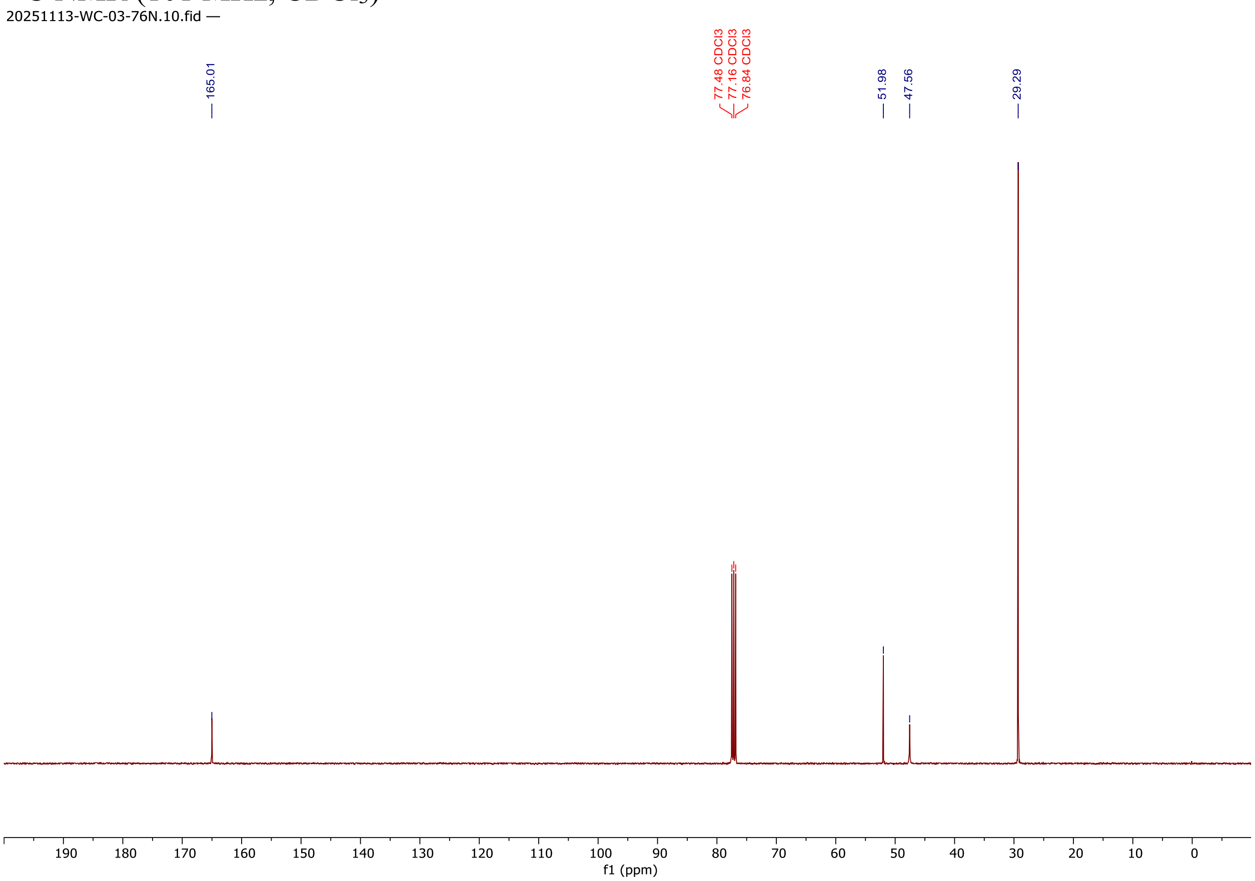

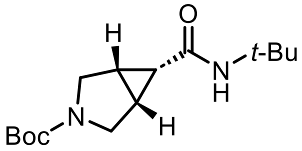

***tert*-Butyl *endo*-6-(*tert*-butylcarbamoyl)-3-azabicyclo[3.1.0]hexane-3-carboxylate (*endo*-14)**

^{1}H NMR (400 MHz, $CDCl_3$)

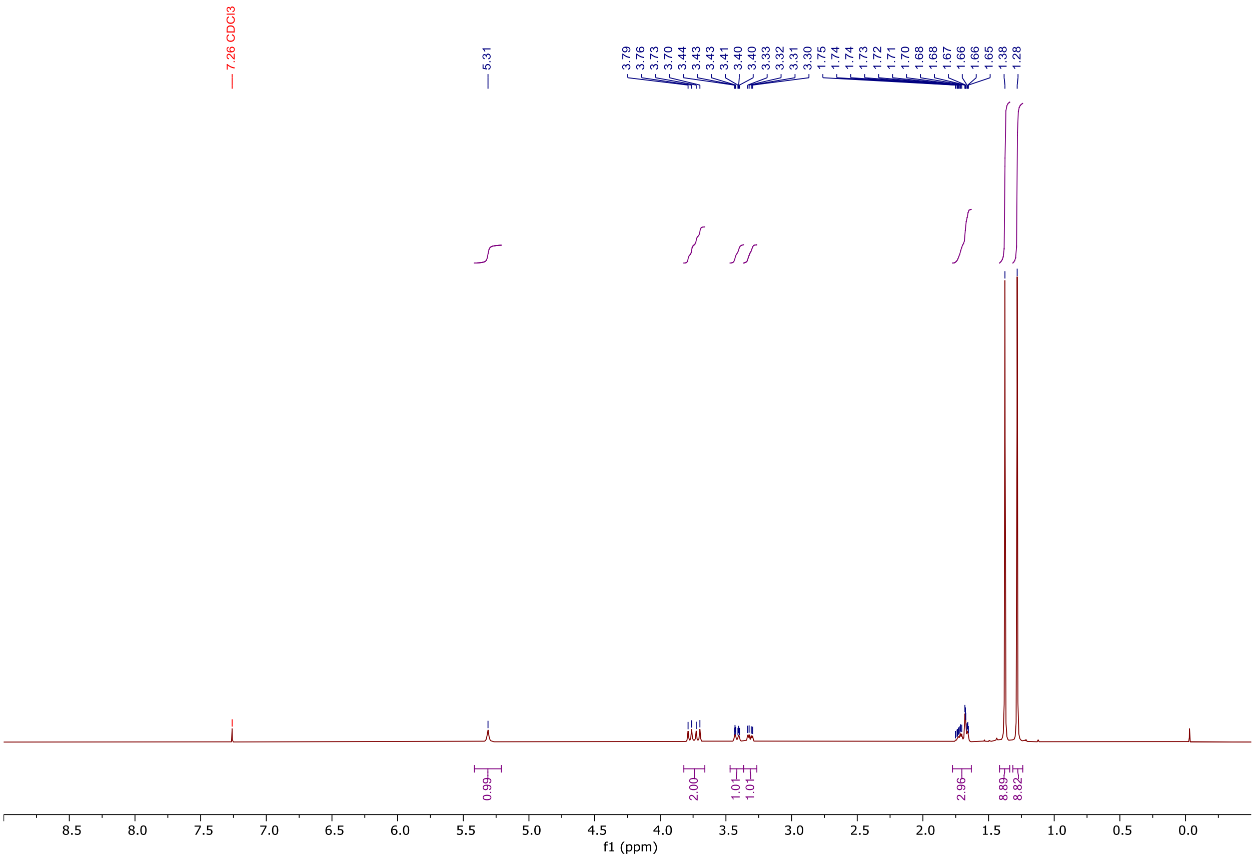

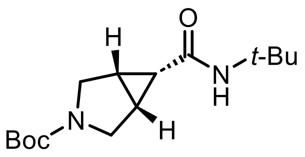

^{13}C NMR (101 MHz, $CDCl_3$)

20251113-WC-03-43N-1.2.fid —

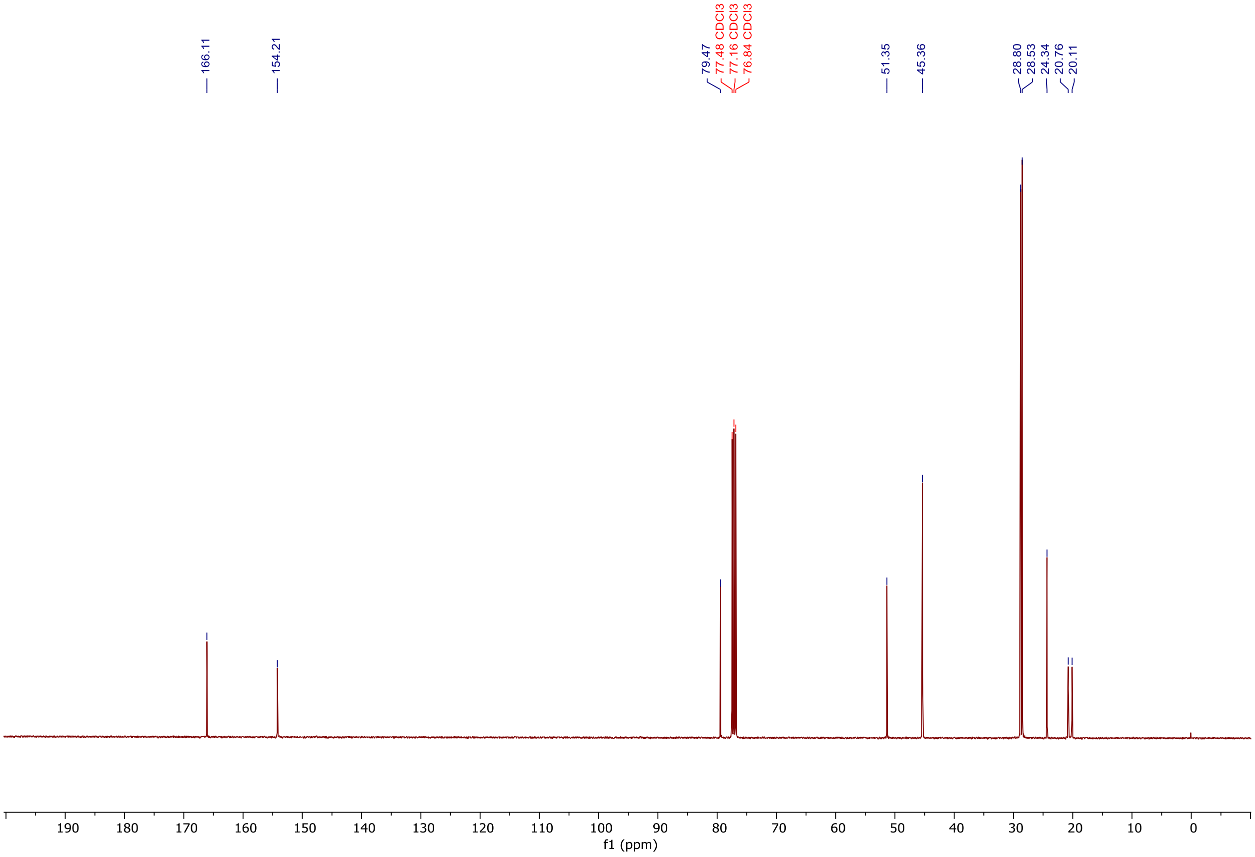

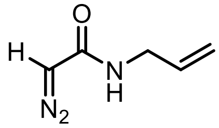

N-allyl-2-diazoacetamide (SI-6)

^{1}H NMR (400 MHz, $CDCl_3$)

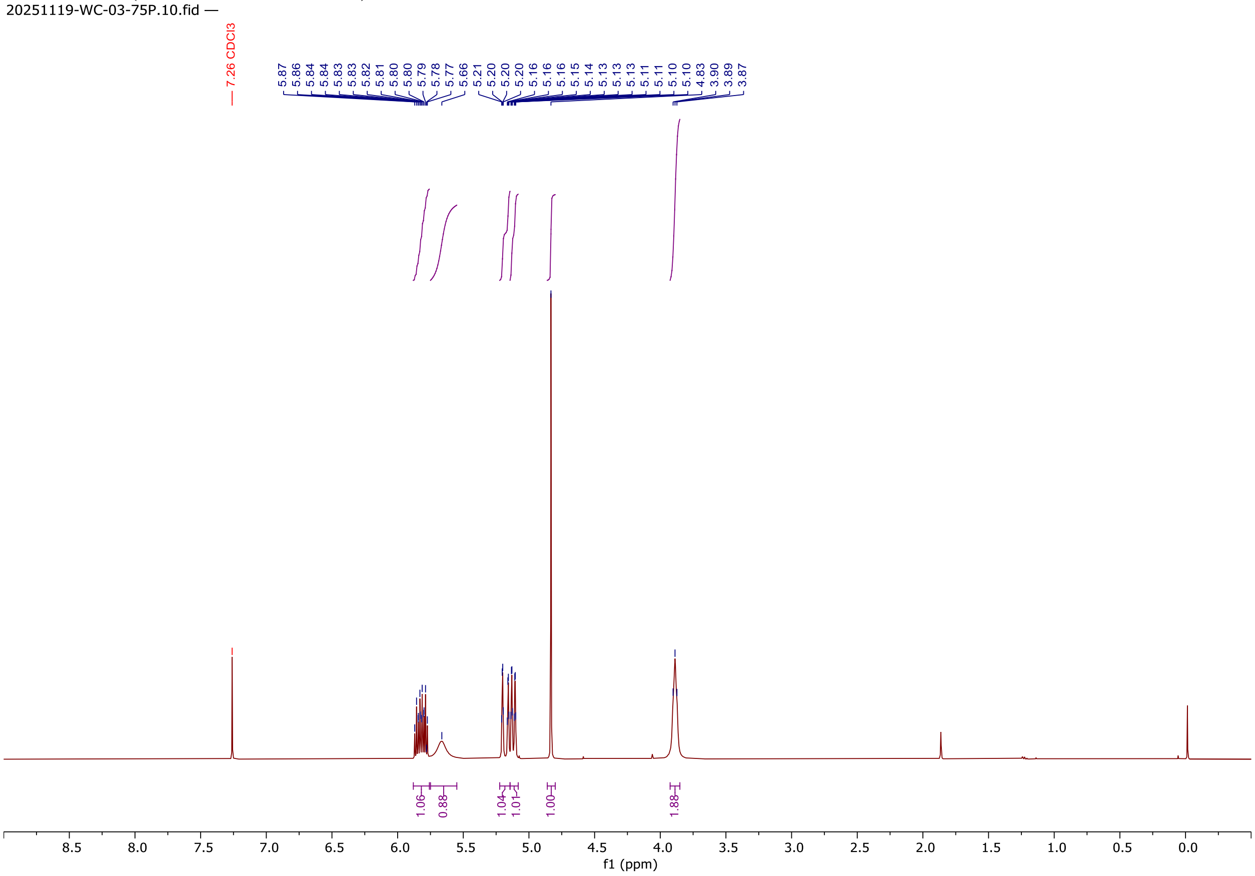

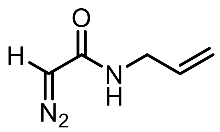

^{13}C NMR (101 MHz, $CDCl_3$)

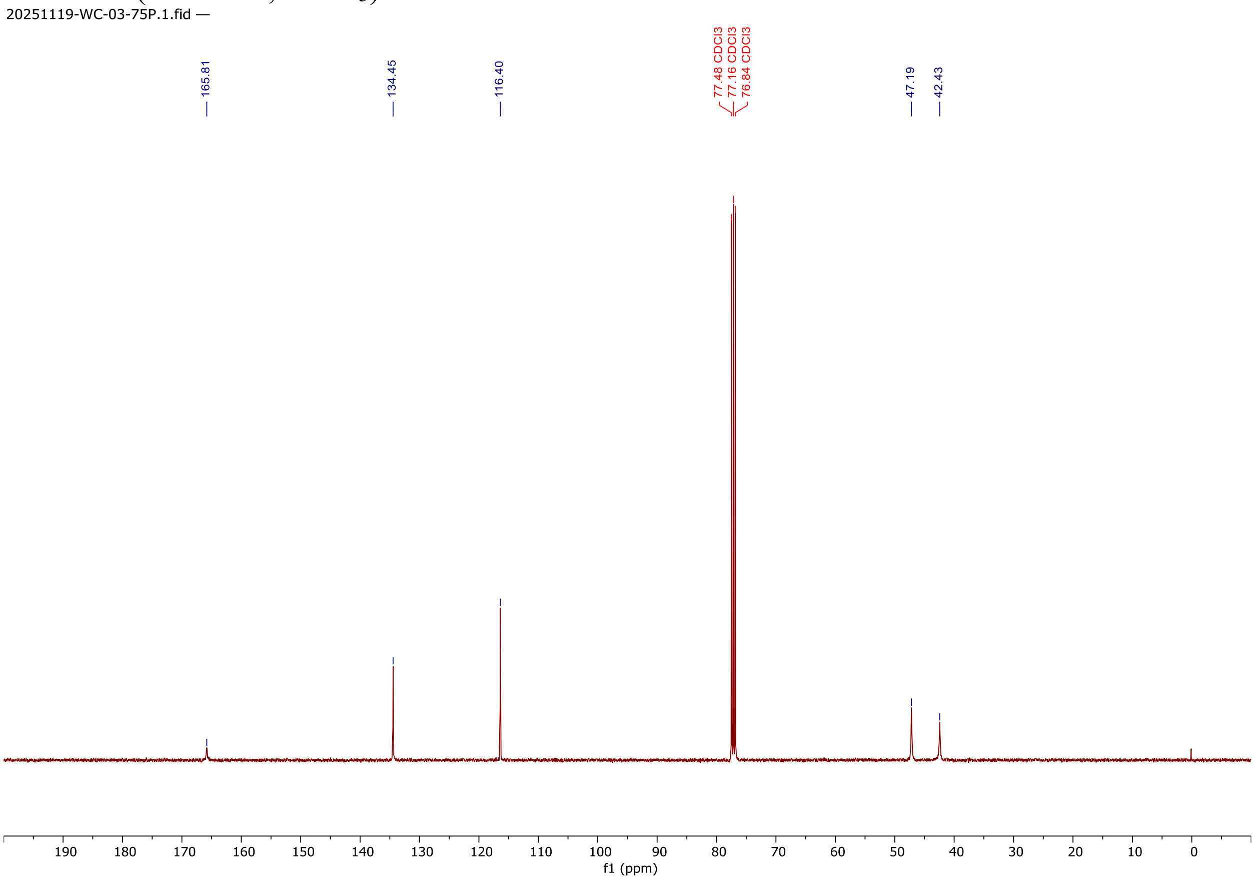

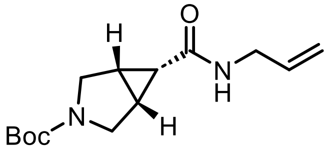

***tert*-Butyl *endo*-6-(allylcarbamoyl)-3-azabicyclo[3.1.0]hexane-3-carboxylate (*endo*-15)**

^{1}H NMR (400 MHz, $CDCl_3$)

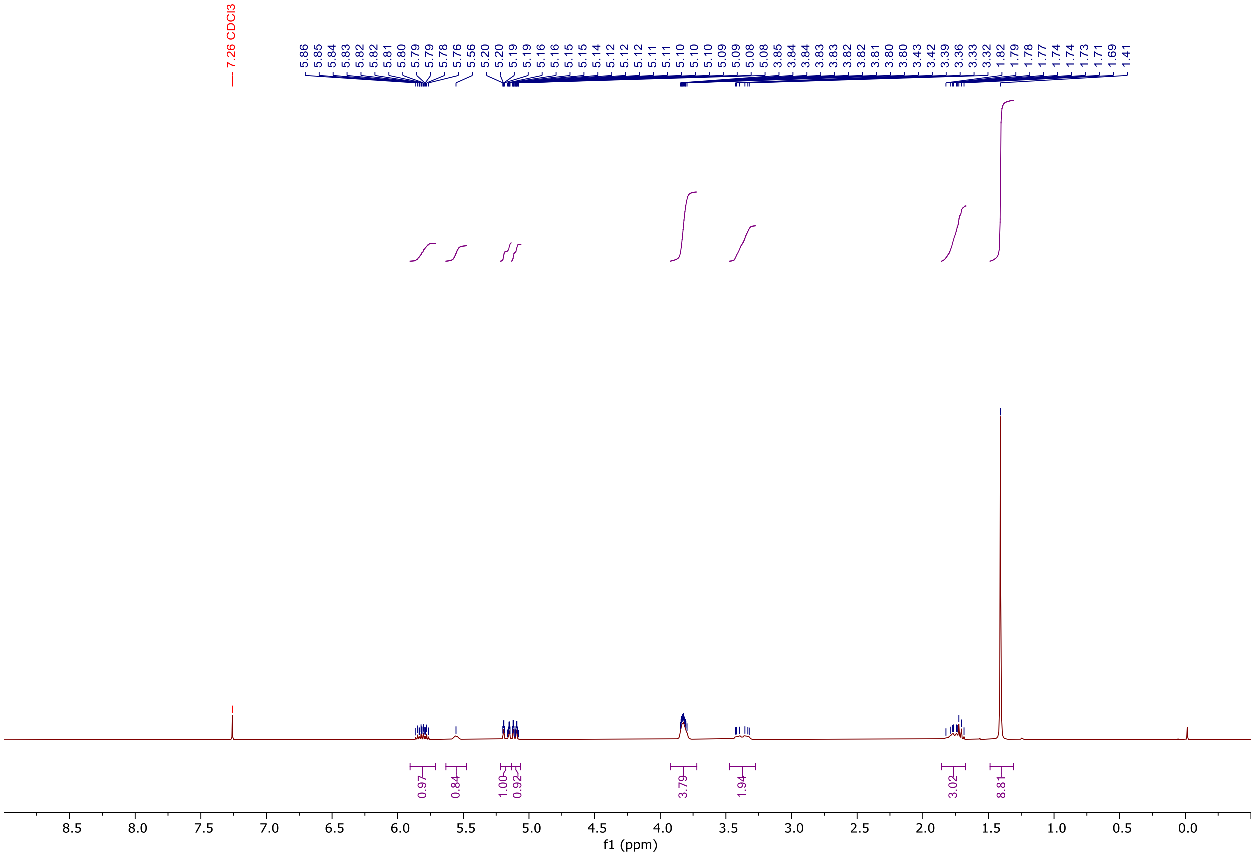

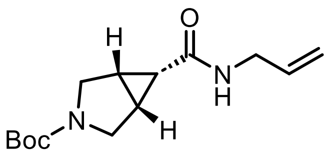

^{13}C NMR (101 MHz, $CDCl_3$)

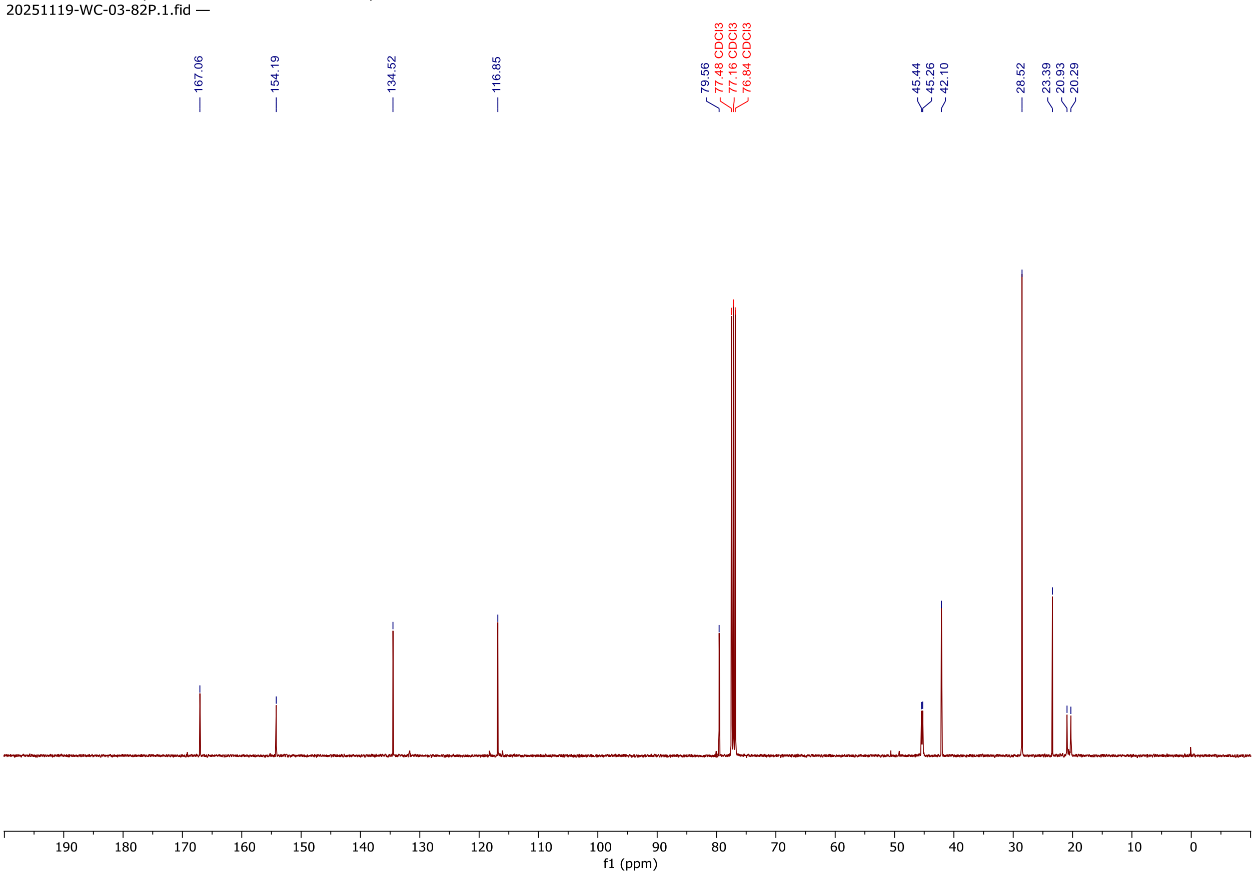

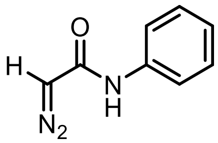

2-diazo-N-phenylacetamide (SI-7)

^{1}H NMR (400 MHz, $CDCl_3$)

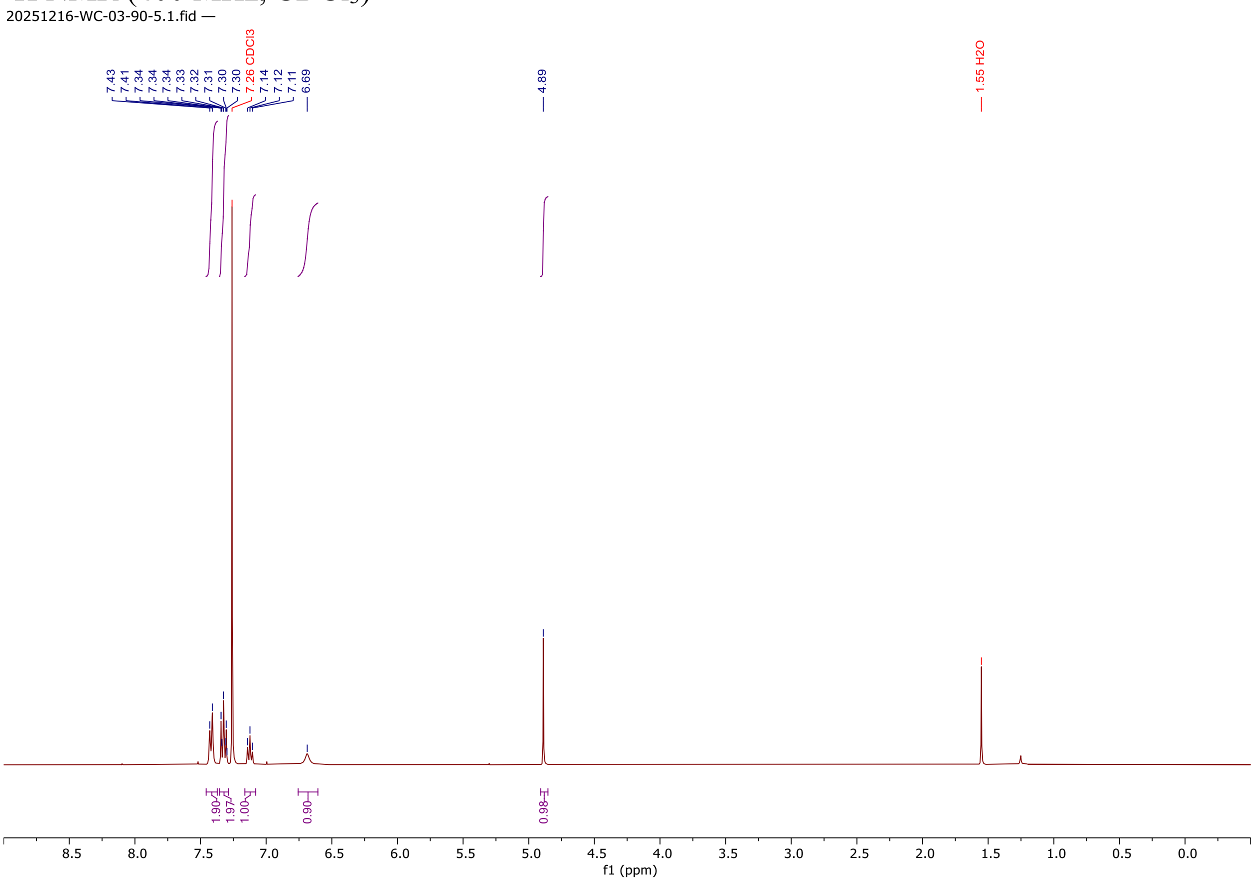

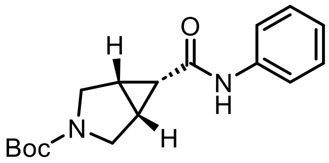

tert-Butyl endo-6-(phenylcarbamoyl)-3-azabicyclo[3.1.0]hexane-3-carboxylate (endo-16)

^{1}H NMR (400 MHz, $CDCl_3$)

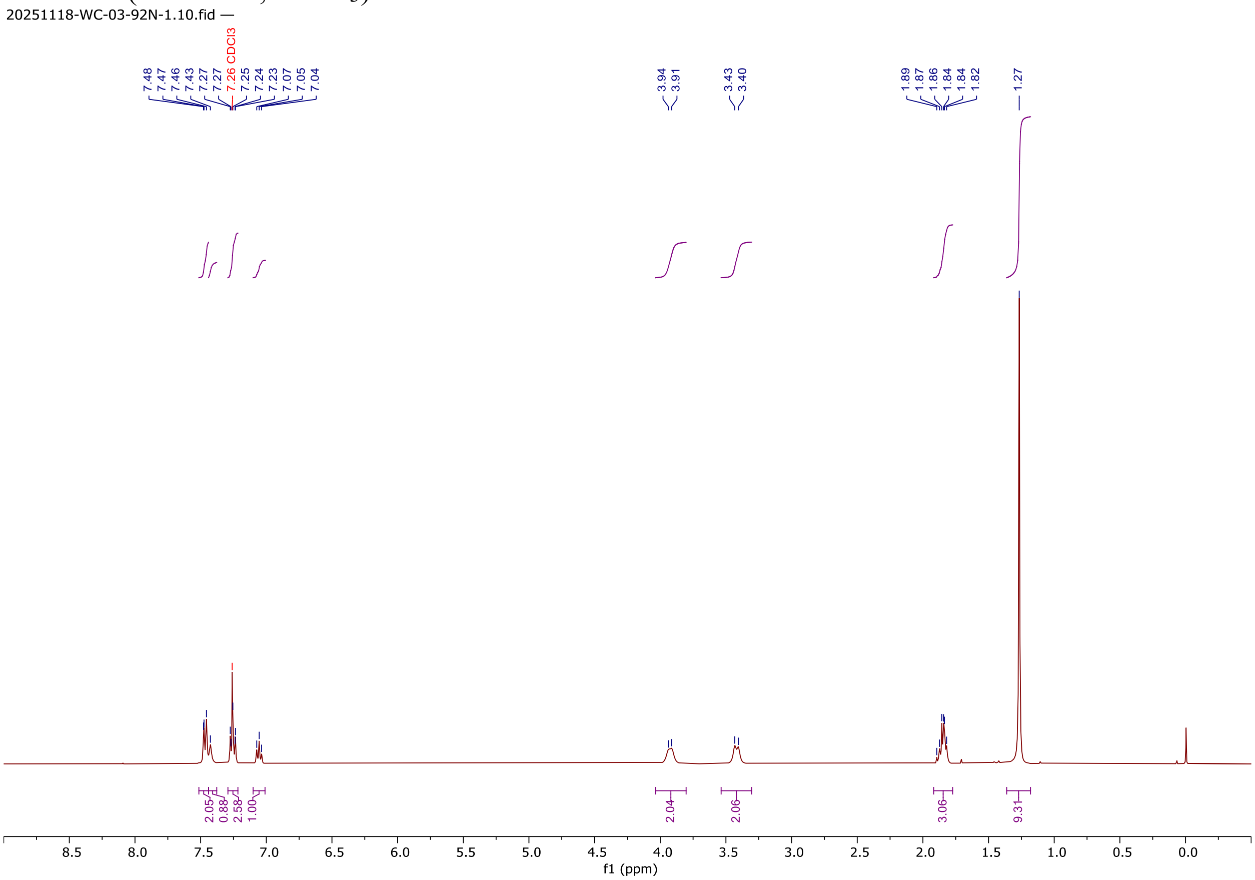

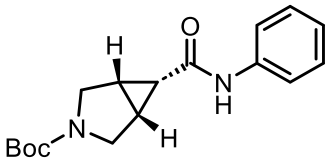

^{13}C NMR (101 MHz, $CDCl_3$)

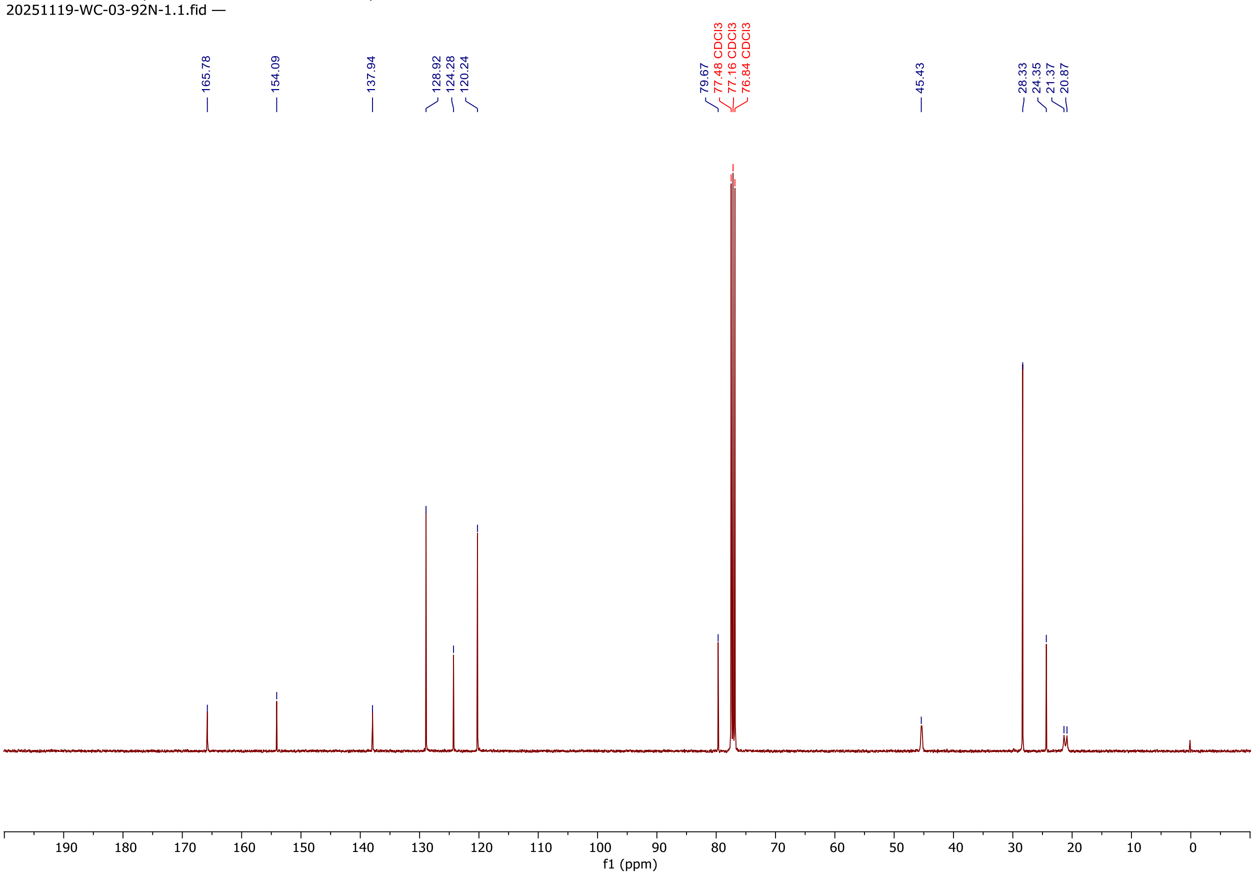

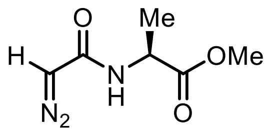

Methyl (2-diazoacetyl)-L-alaninate (SI-8)

^{1}H NMR (400 MHz, $CDCl_3$)

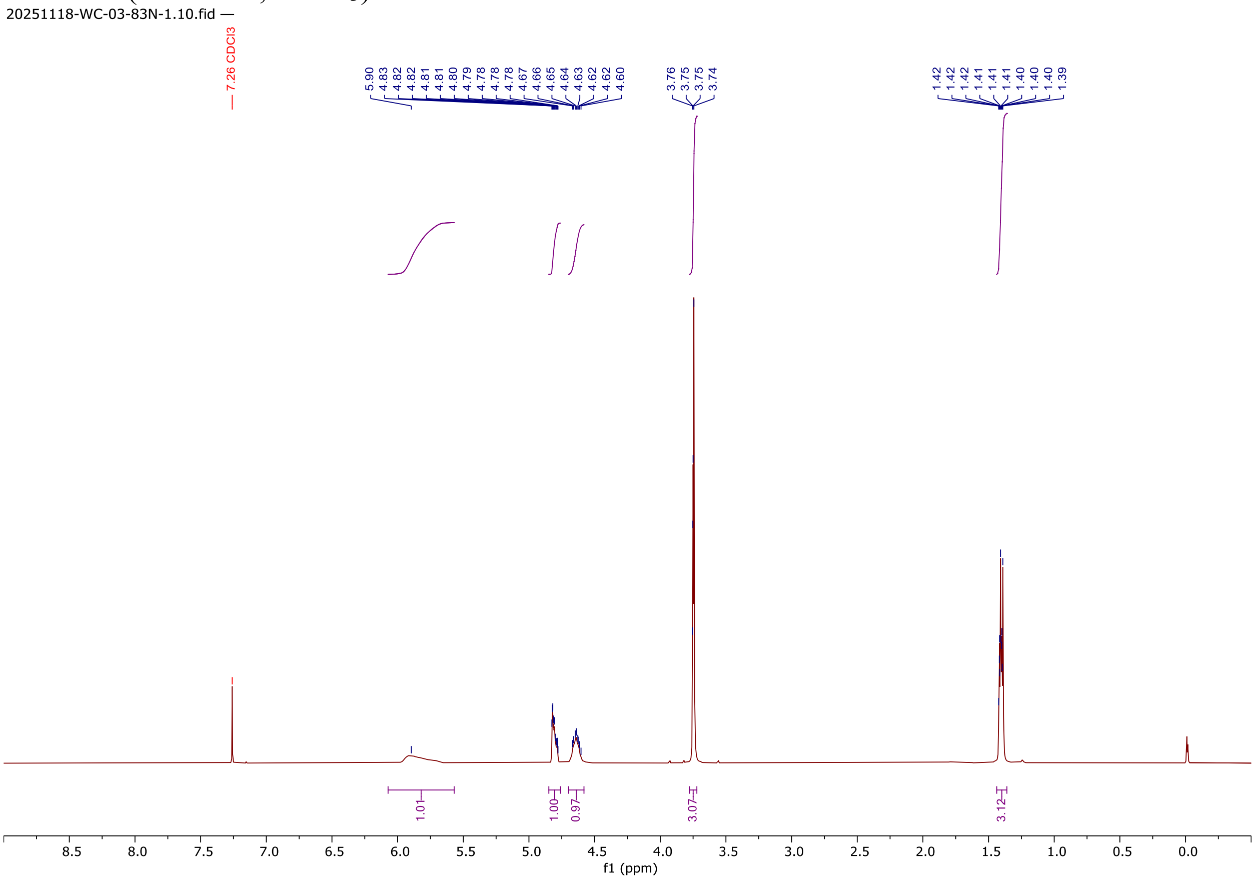

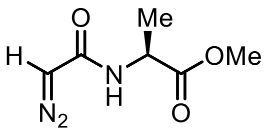

^{13}C NMR (101 MHz, $CDCl_3$)

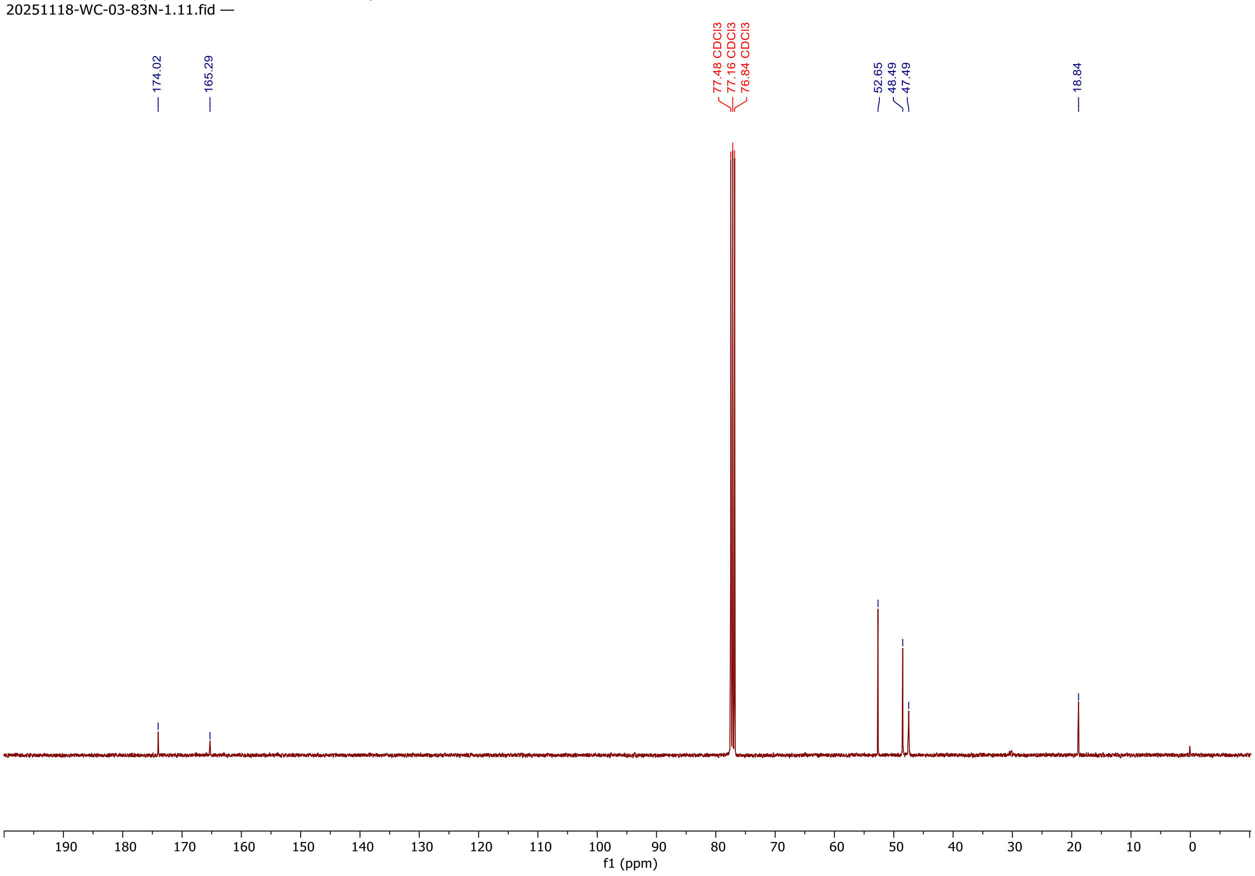

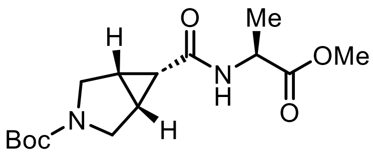

tert-Butyl endo-6-(((S)-1-methoxy-1-oxopropan-2-yl)carbamoyl)-3-azabicyclo[3.1.0]hexane-3-carboxylate (endo-17)

^{1}H NMR (400 MHz, $CDCl_3$)

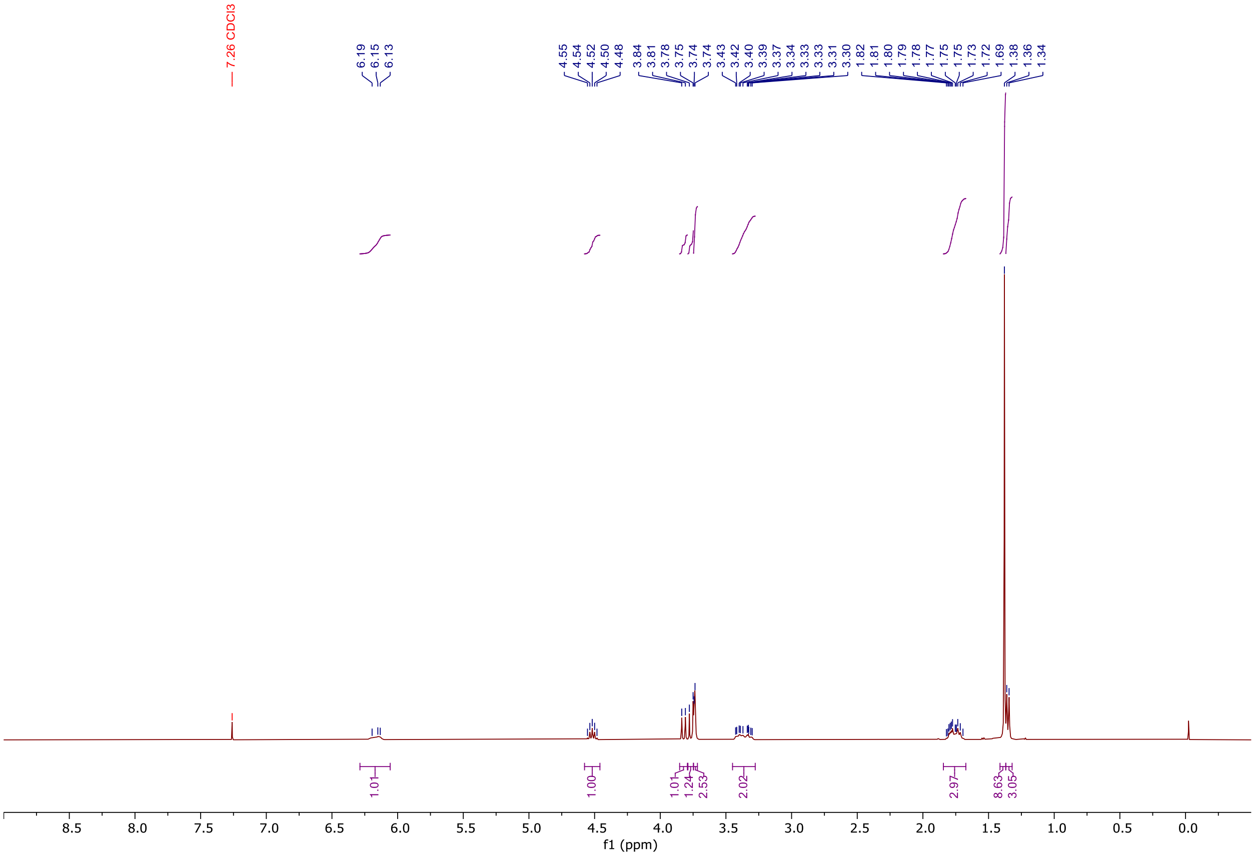

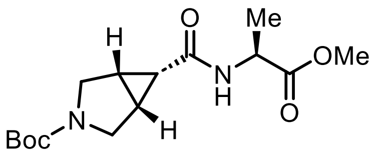

^{13}C NMR (101 MHz, $CDCl_3$)

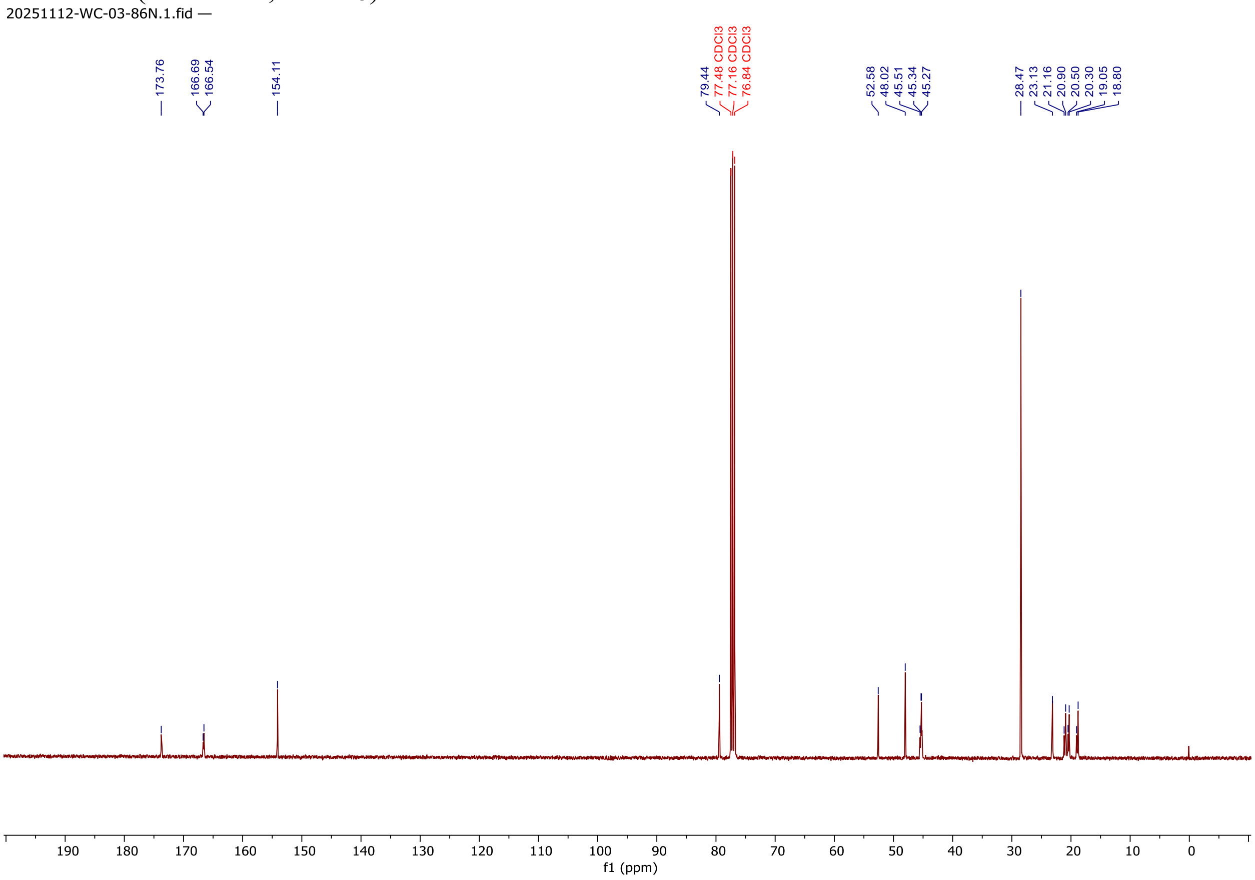

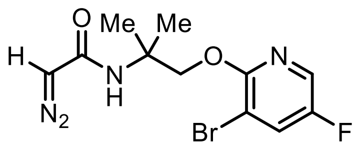

N-(1-((3-bromo-5-fluoropyridin-2-yl)oxy)-2-methylpropan-2-yl)-2-diazoacetamide (SI-10)

^{1}H NMR (400 MHz, $CDCl_3$)

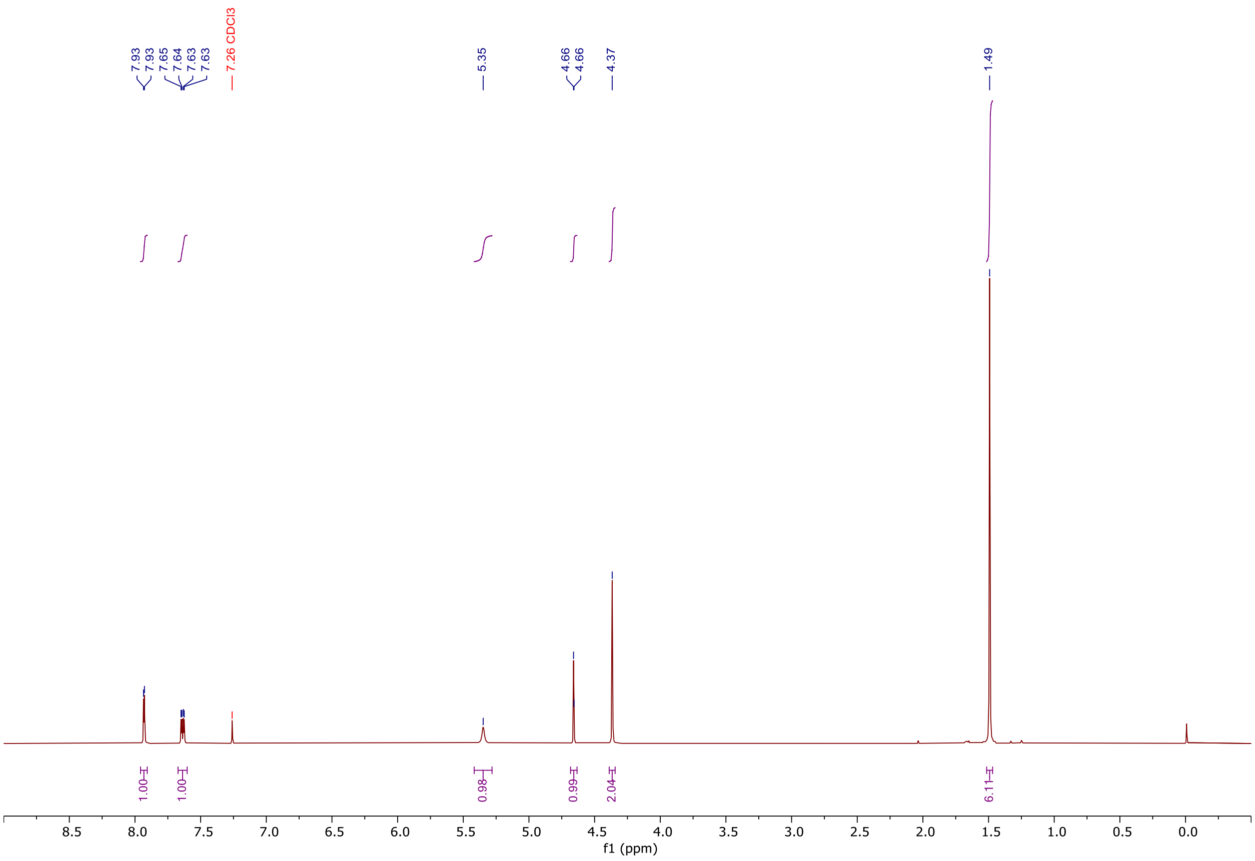

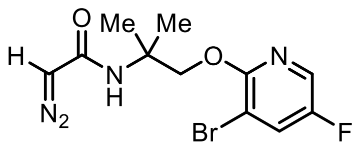

^{19}F NMR (376 MHz, $CDCl_3$)

20251008-WC-03-51.11.fid —

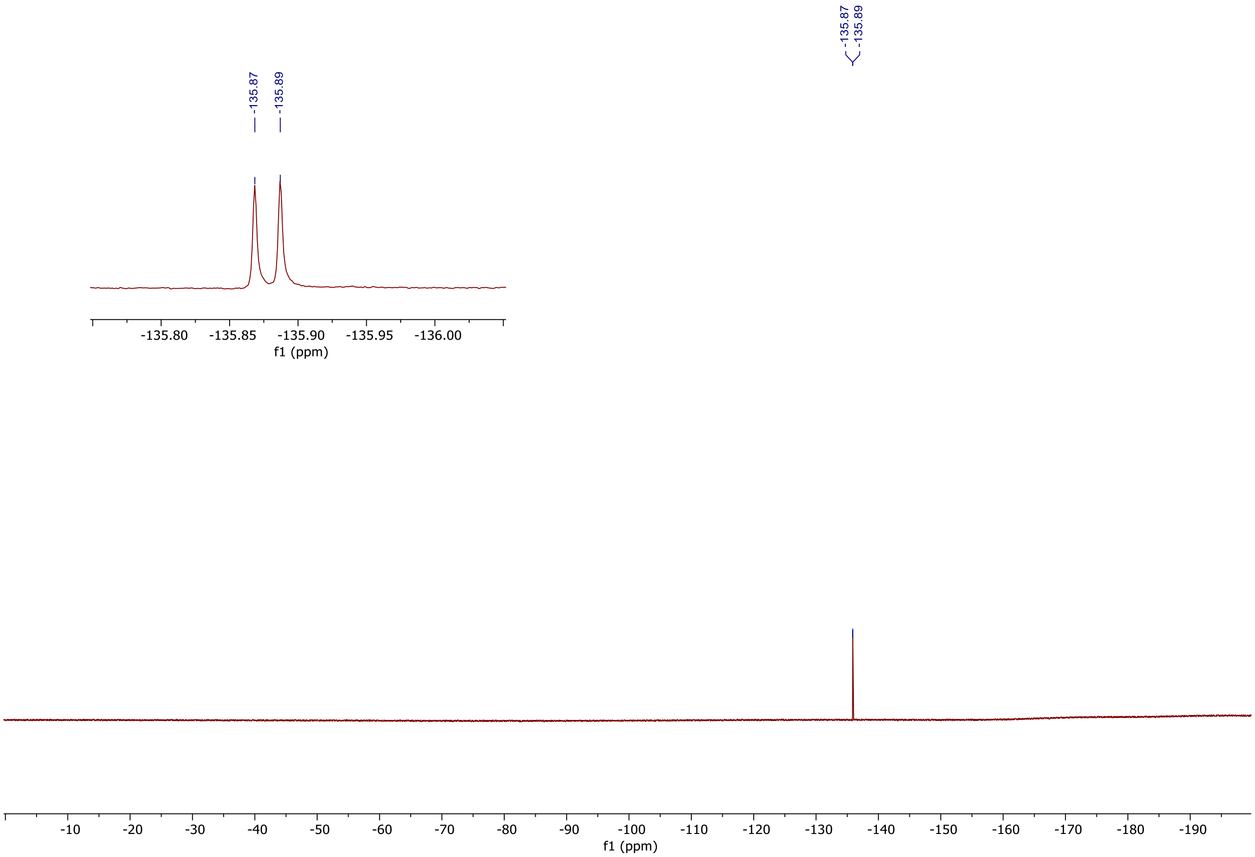

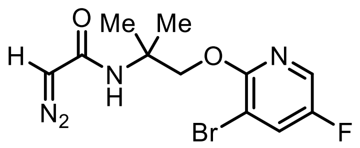

^{13}C NMR (101 MHz, $CDCl_3$)

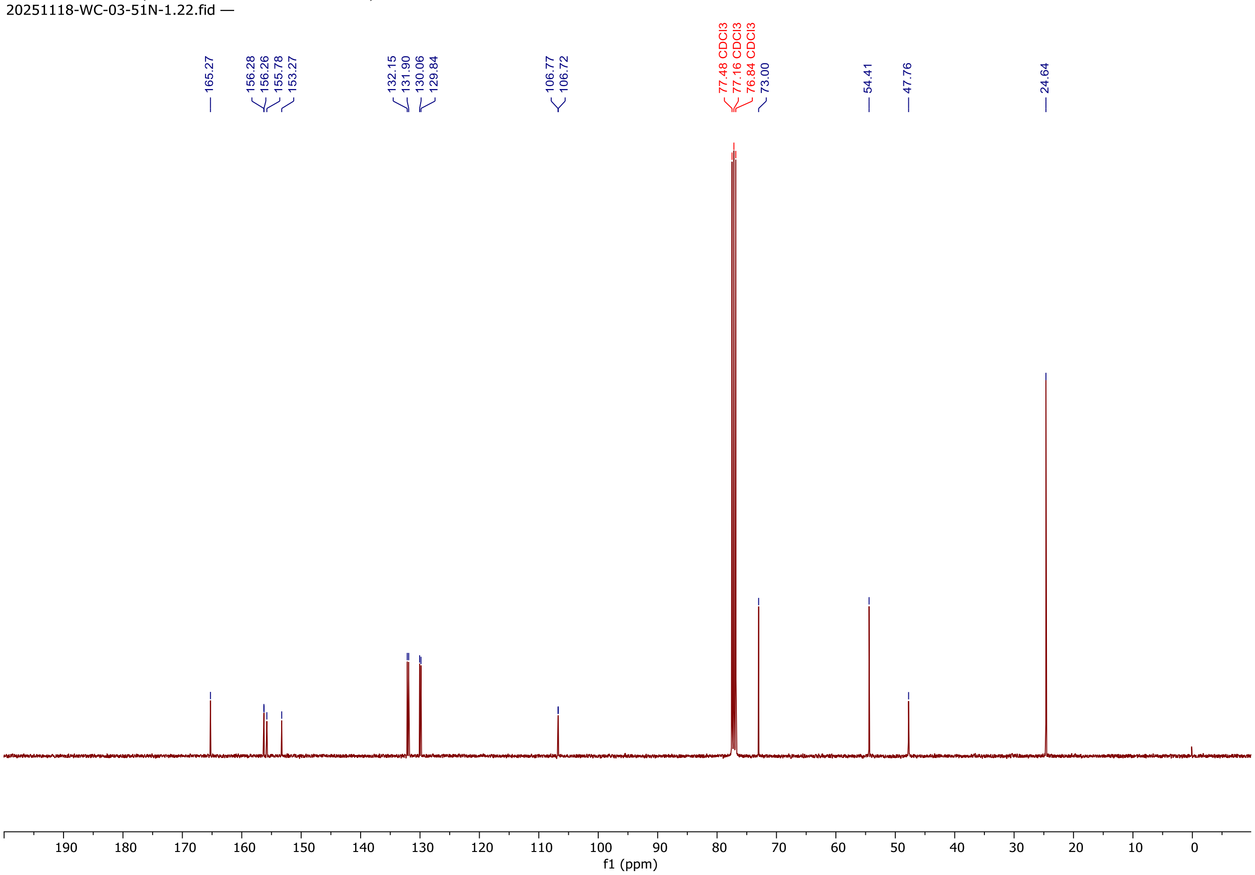

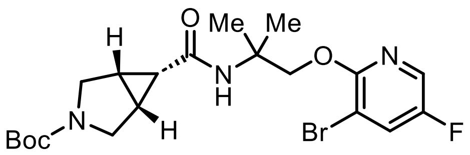

***tert*-Butyl *endo*-6-((1-((3-bromo-5-fluoropyridin-2-yl)oxy)-2-methylpropan-2-yl)carbamoyl)-3-azabicyclo[3.1.0]hexane-3-carboxylate (*endo*-18)**

^{1}H NMR (400 MHz, $CDCl_3$)

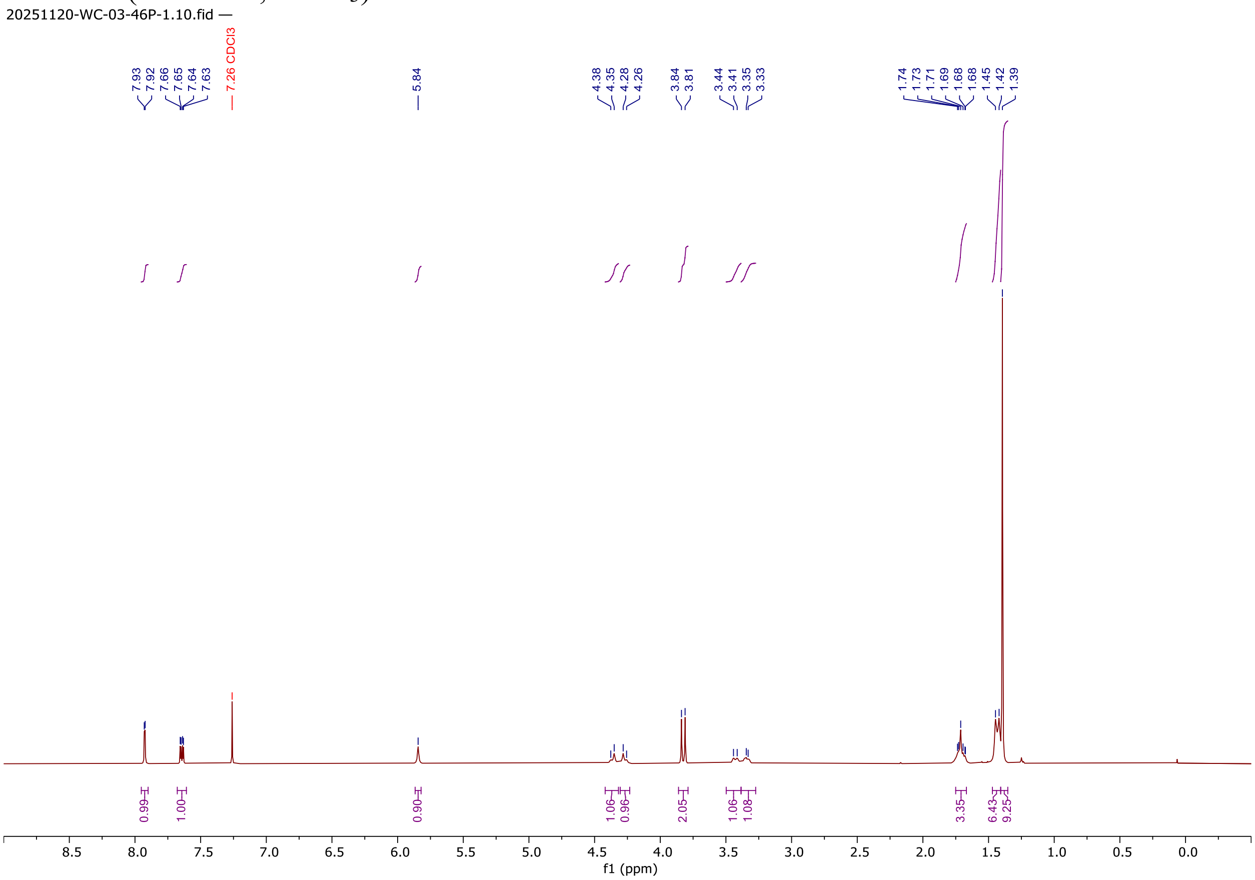

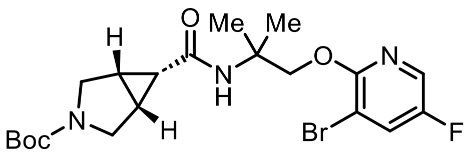

^{19}F NMR (376 MHz, $CDCl_3$)

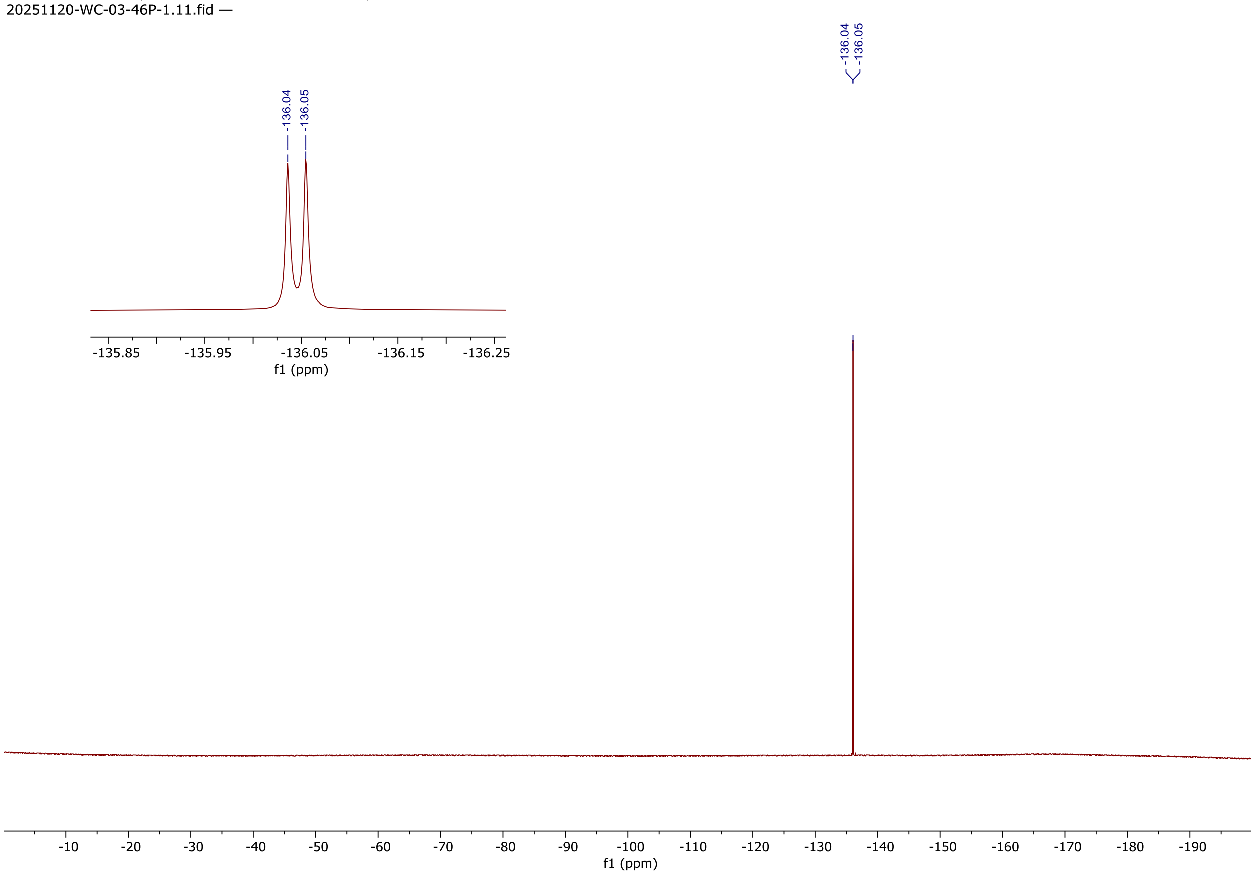

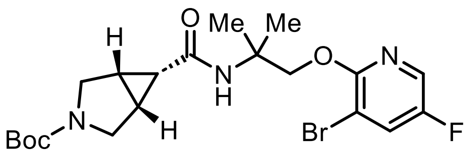

^{13}C NMR (101 MHz, $CDCl_3$)

20251120-WC-03-46P-1.12.fid —

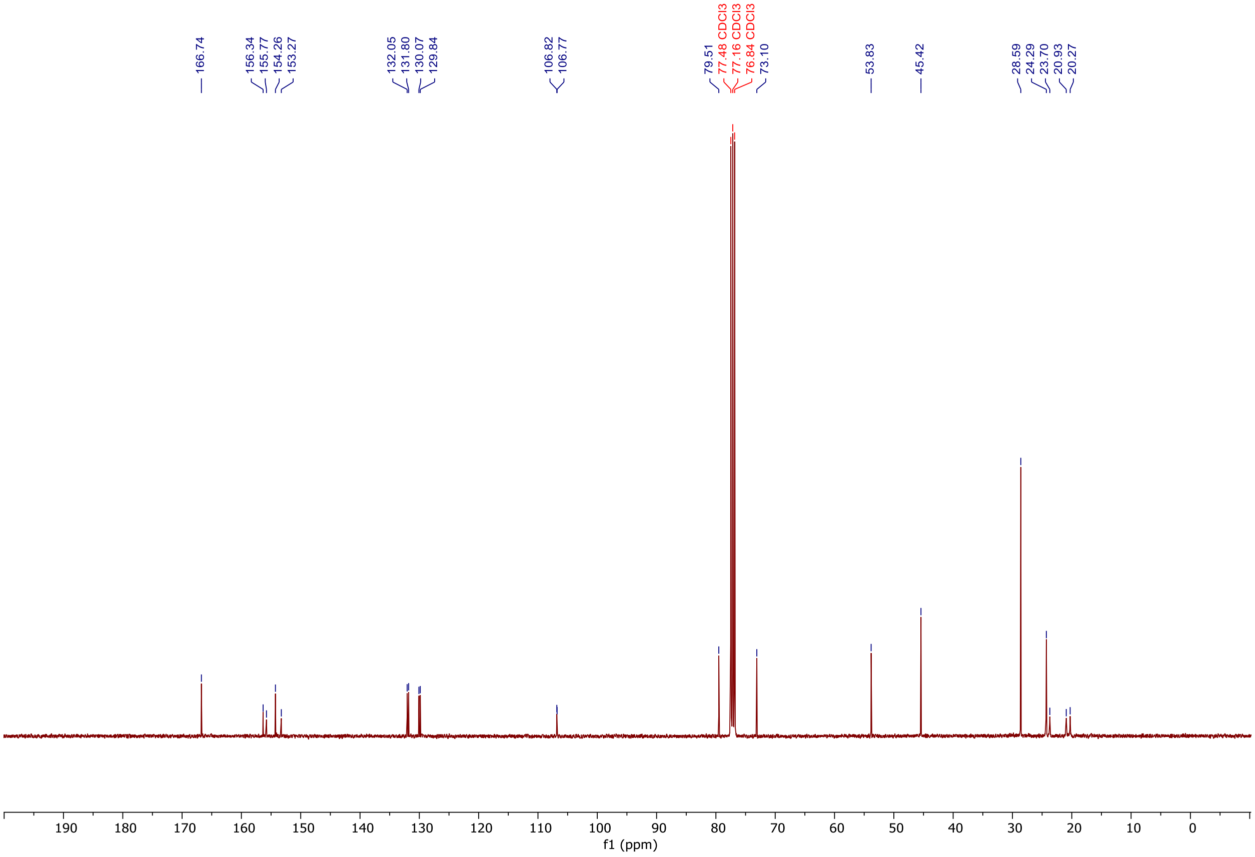

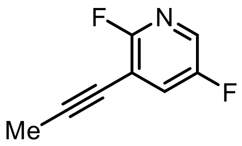

2,5-Difluoro-3-(prop-1-yn-1-yl)pyridine (SI-11)

^{1}H NMR (400 MHz, $CDCl_3$)

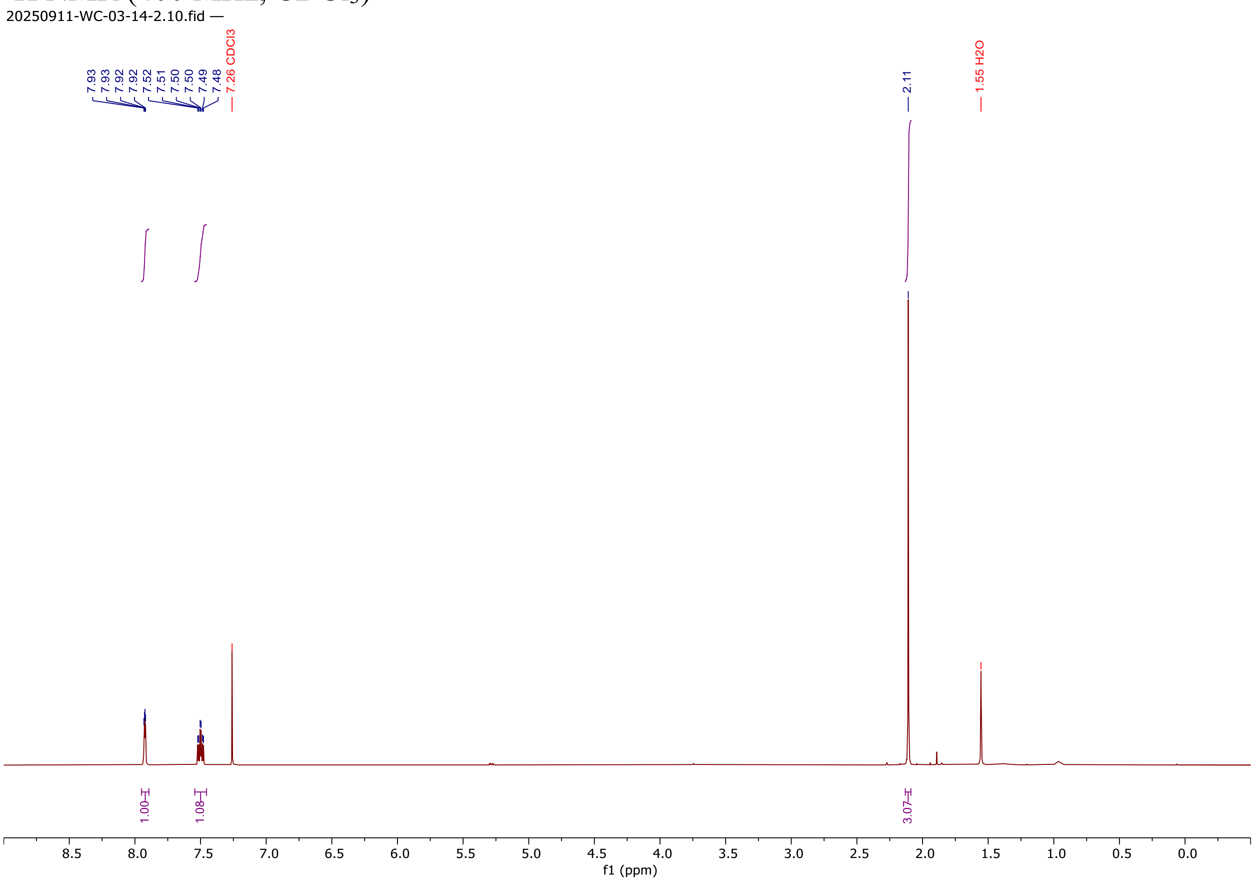

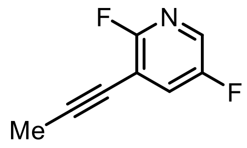

^{19}F NMR (376 MHz, $CDCl_3$)

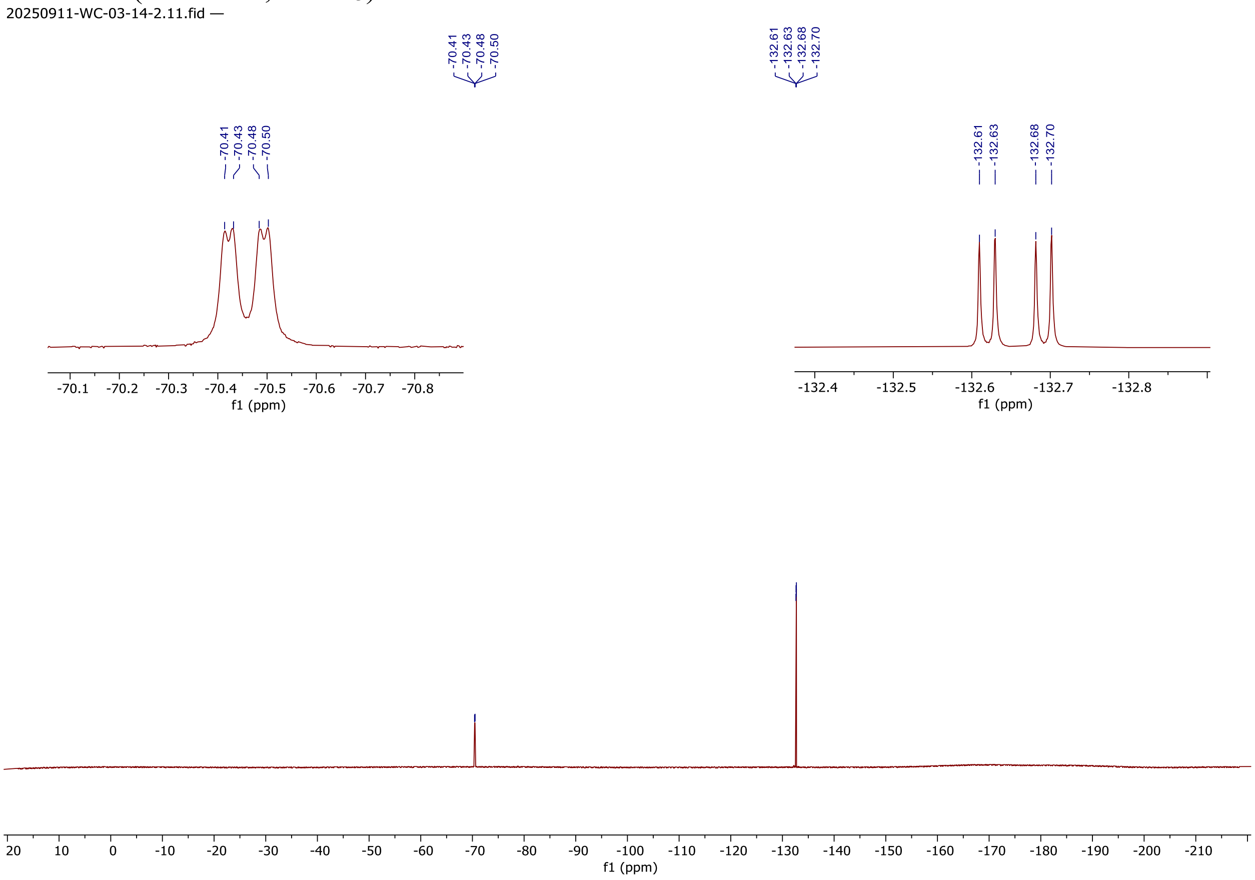

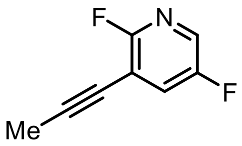

^{13}C NMR (101 MHz, $CDCl_3$)

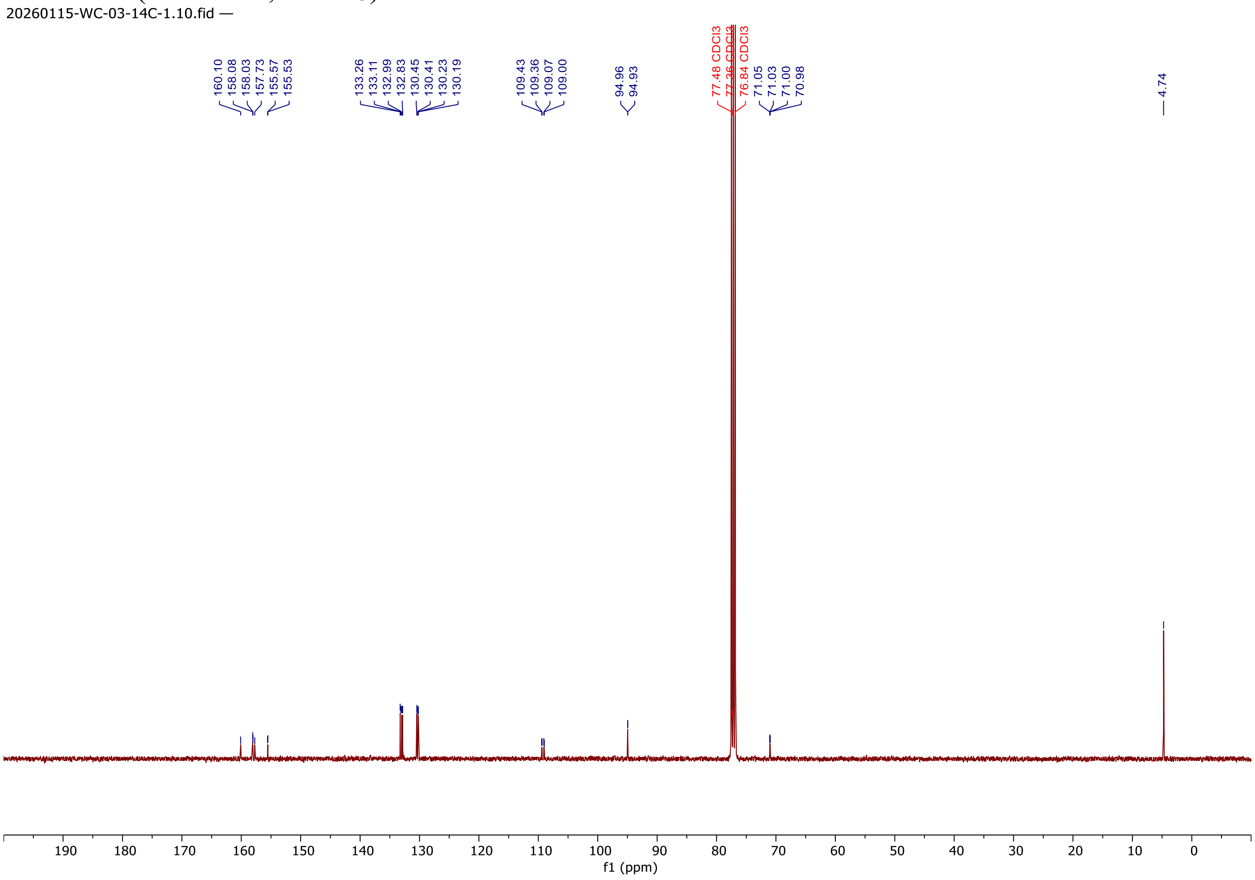

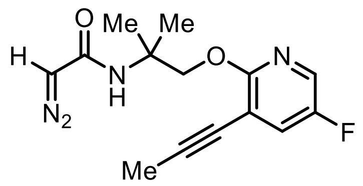

2-Diazo-N-(1-((5-fluoro-3-(prop-1-yn-1-yl)pyridin-2-yl)oxy)-2-methylpropan-2-yl)acetamide (SI-13)

^{1}H NMR (400 MHz, $CDCl_3$)

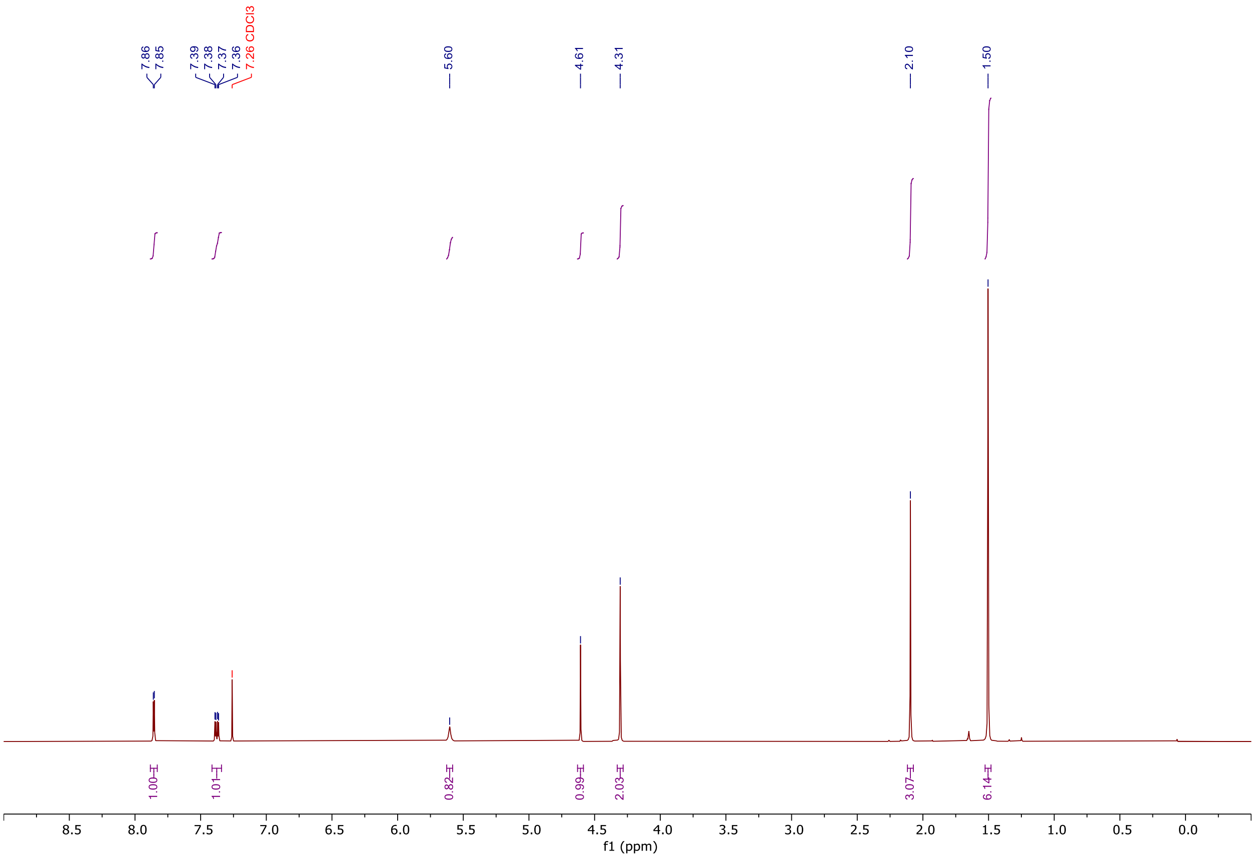

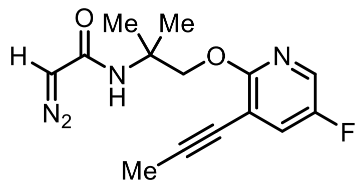

^{19}F NMR (376 MHz, $CDCl_3$)

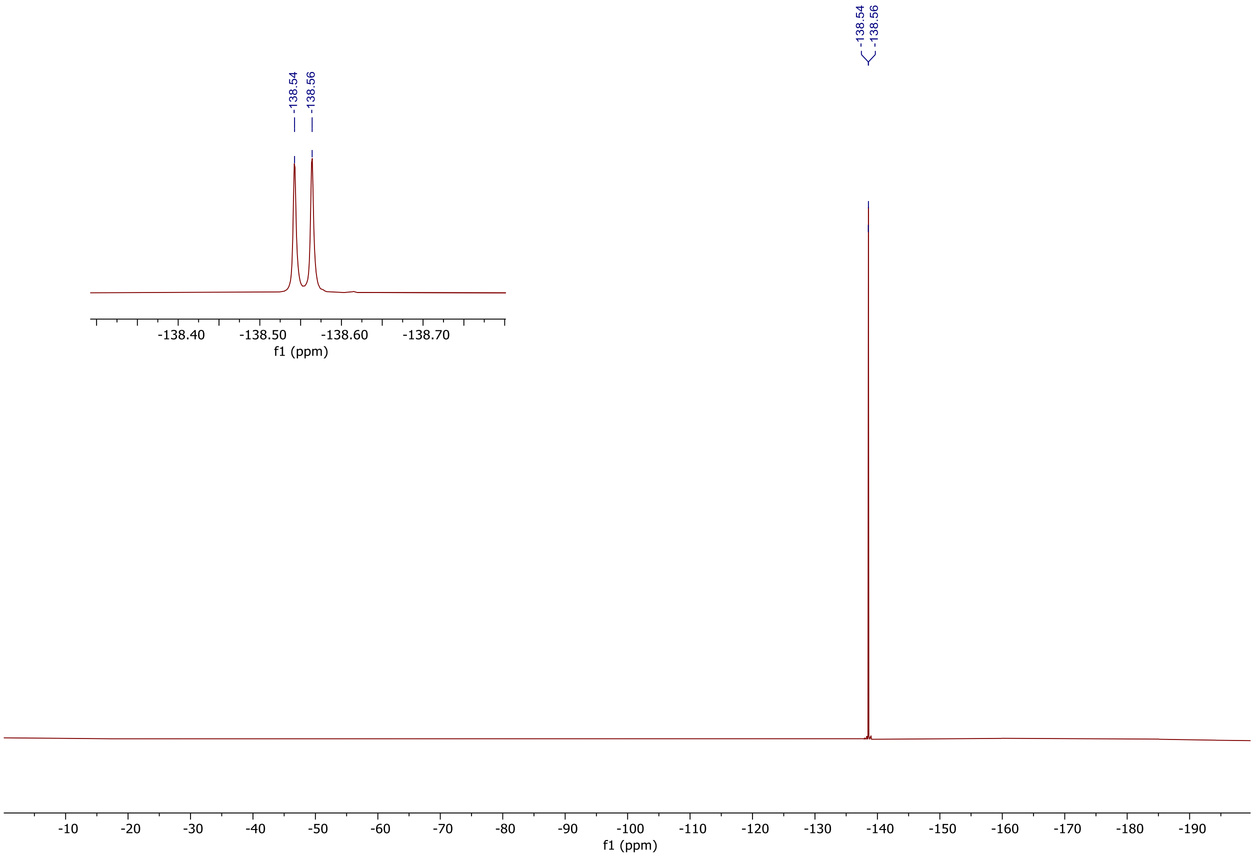

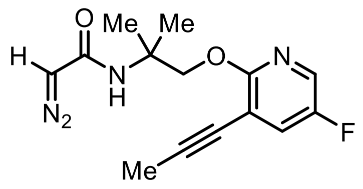

^{13}C NMR (101 MHz, $CDCl_3$)

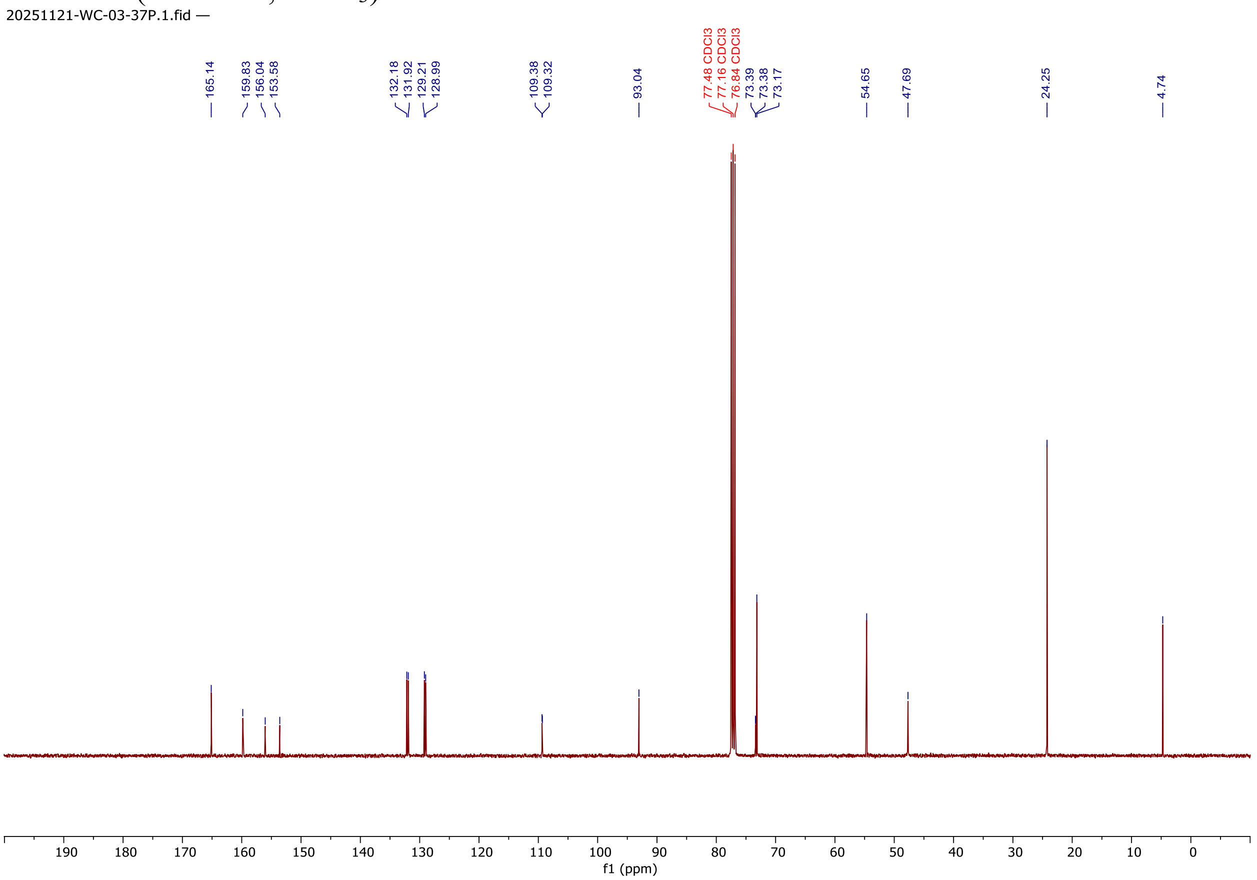

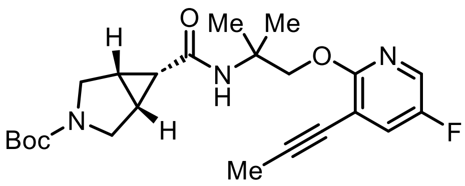

***tert*-Butyl *endo*-6-((1-((5-fluoro-3-(prop-1-yn-1-yl)pyridin-2-yl)oxy)-2-methylpropan-2-yl)carbamoyl)-3-azabicyclo[3.1.0]hexane-3-carboxylate (*endo*-19)**

^{1}H NMR (400 MHz, $CDCl_3$)

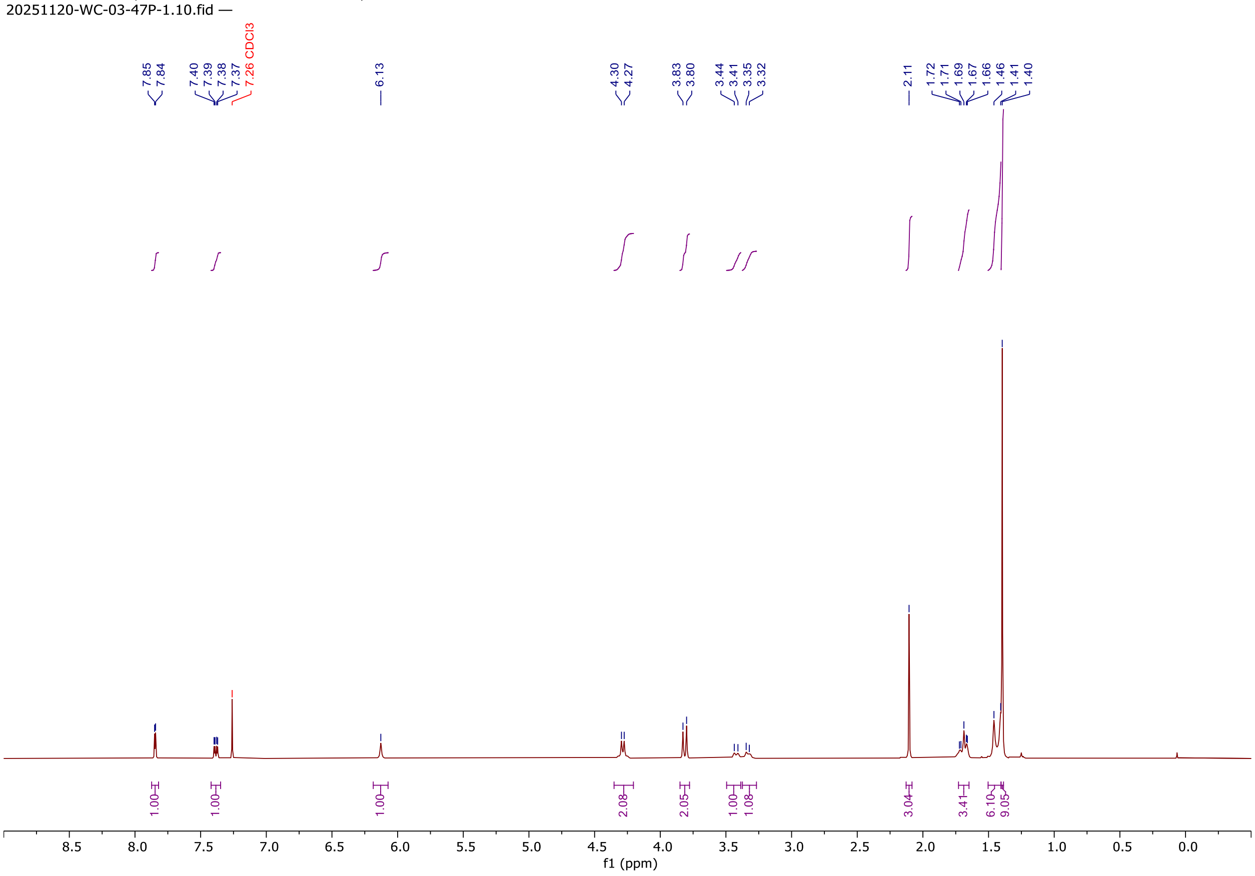

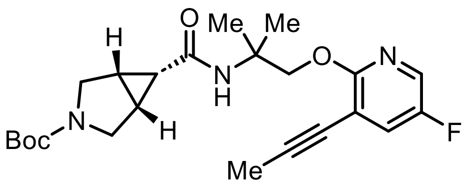

^{19}F NMR (376 MHz, $CDCl_3$)

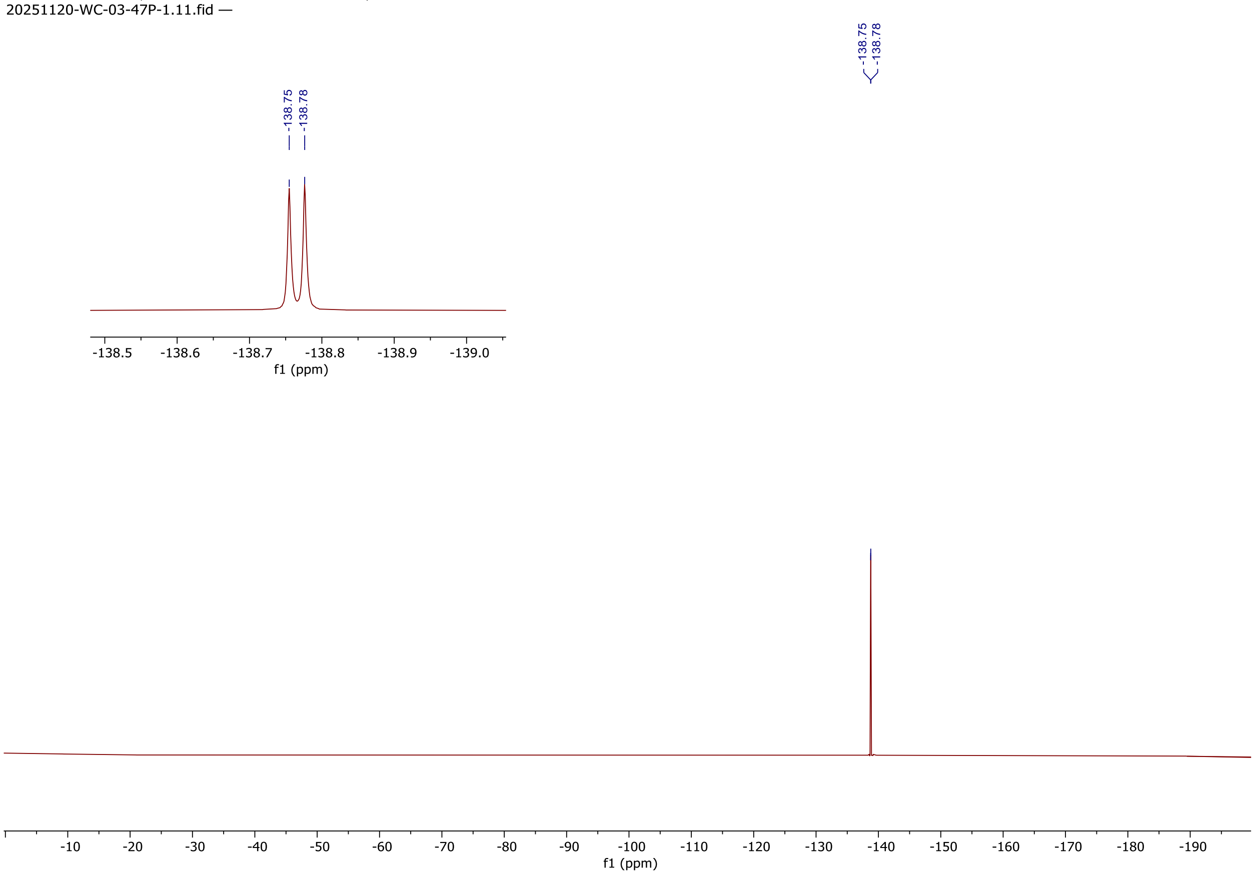

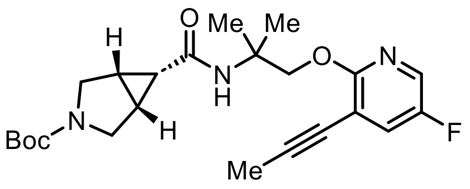

^{13}C NMR (101 MHz, $CDCl_3$)

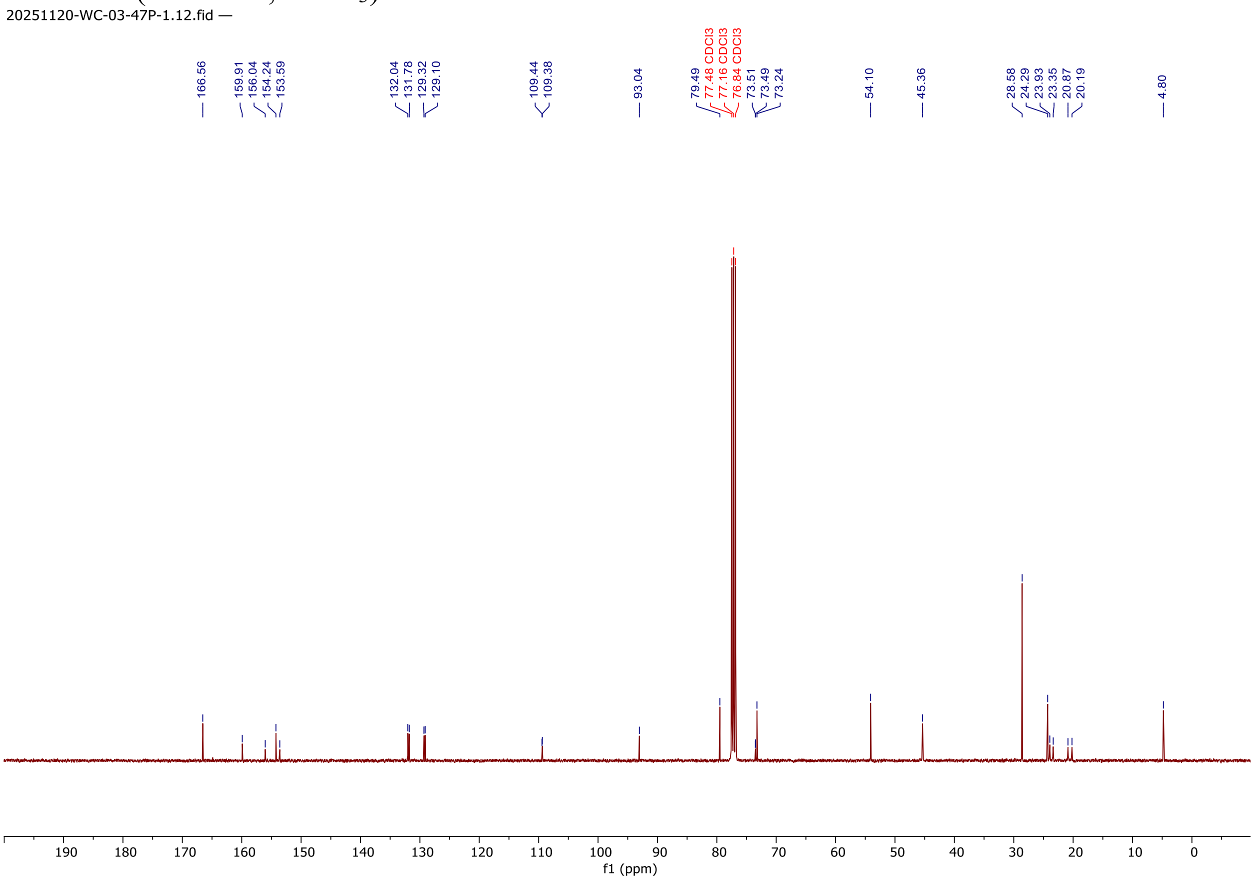

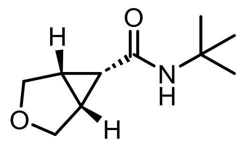

endo-N-(_tert_-Butyl)-3-oxabicyclo[3.1.0]hexane-6-carboxamide (_endo_-21)

^{1}H NMR (400 MHz, $CDCl_3$)

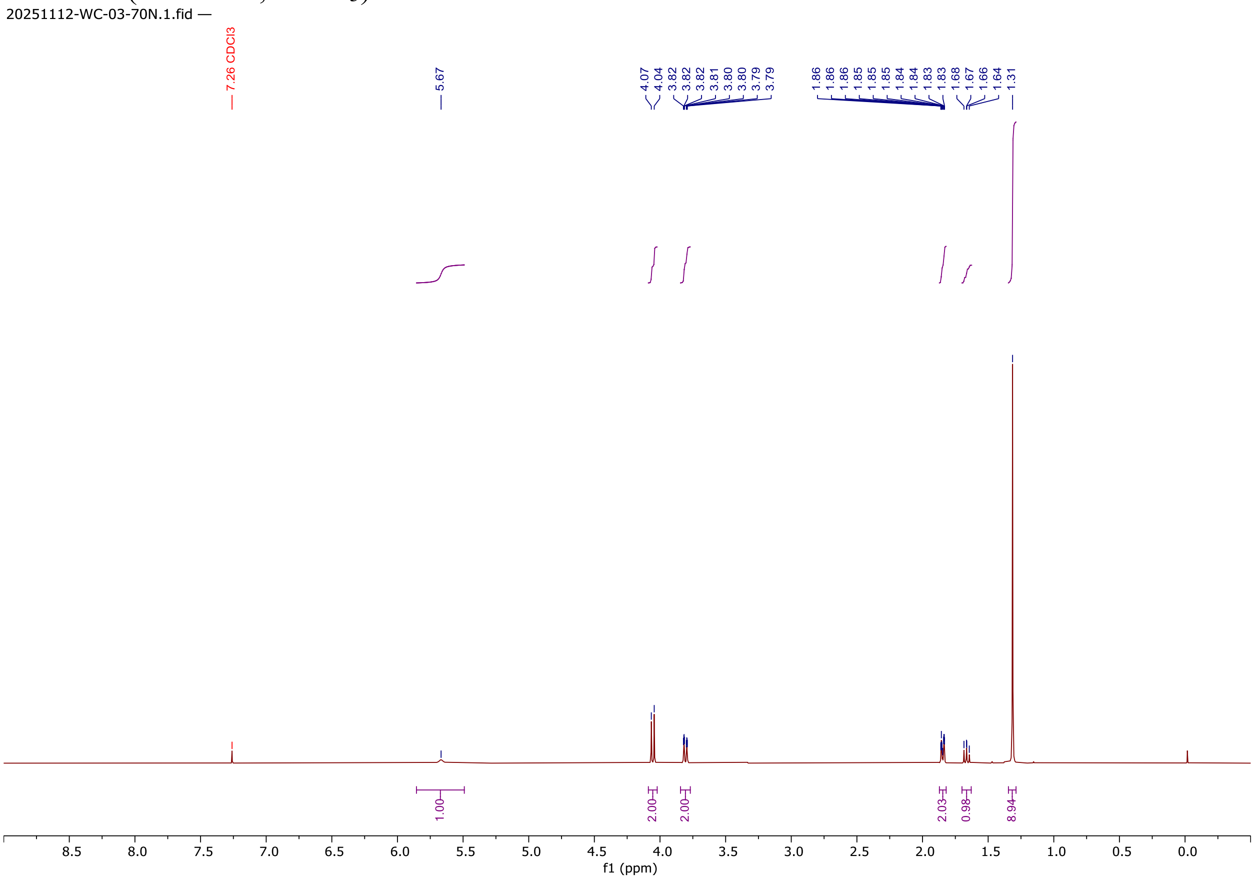

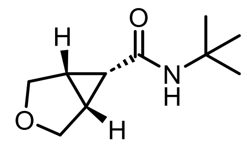

^{13}C NMR (101 MHz, $CDCl_3$)

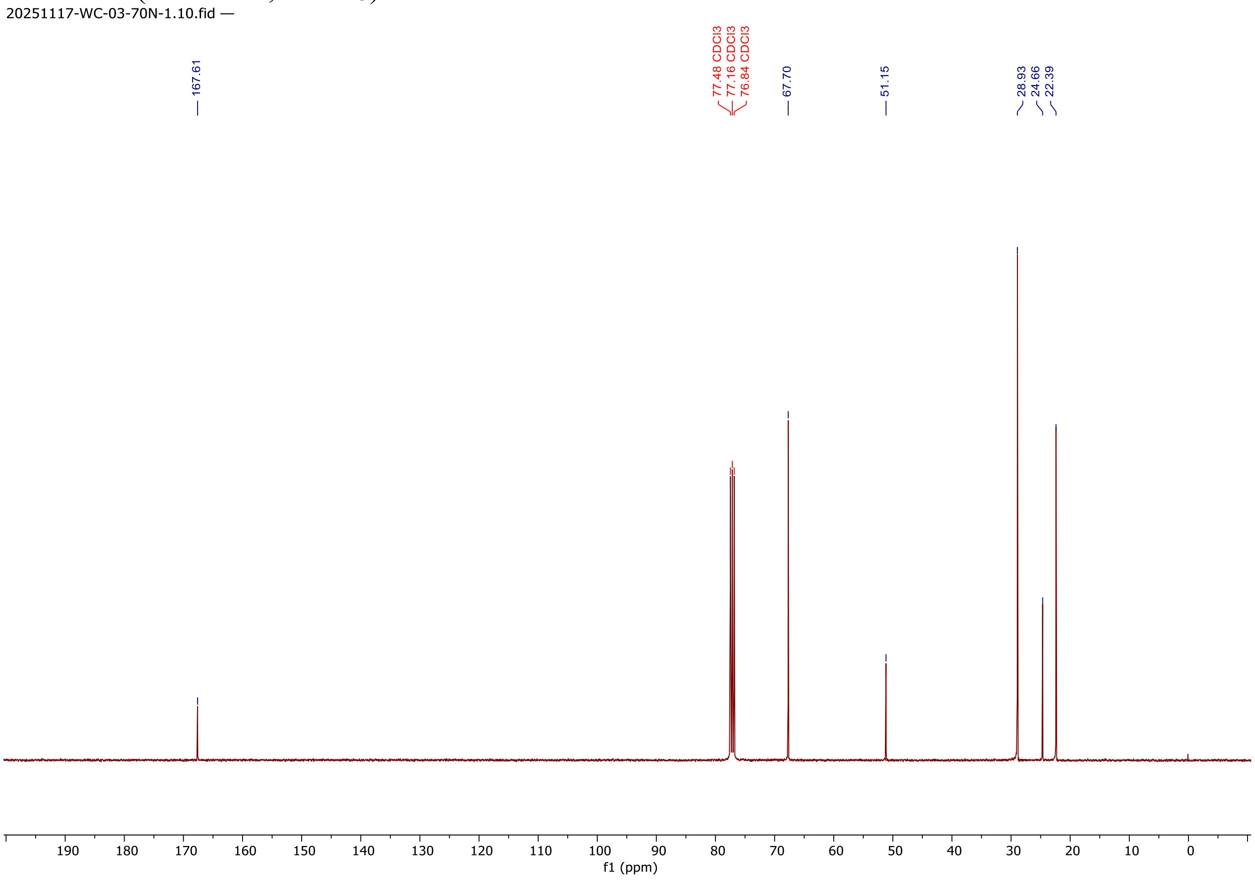

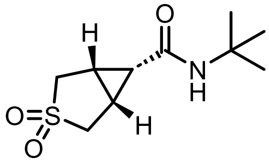

***endo*-N-(*tert*-Butyl)-3-thiabicyclo[3.1.0]hexane-6-carboxamide 3,3-dioxide (*endo*-22)**

^{1}H NMR (400 MHz, $CDCl_3$)

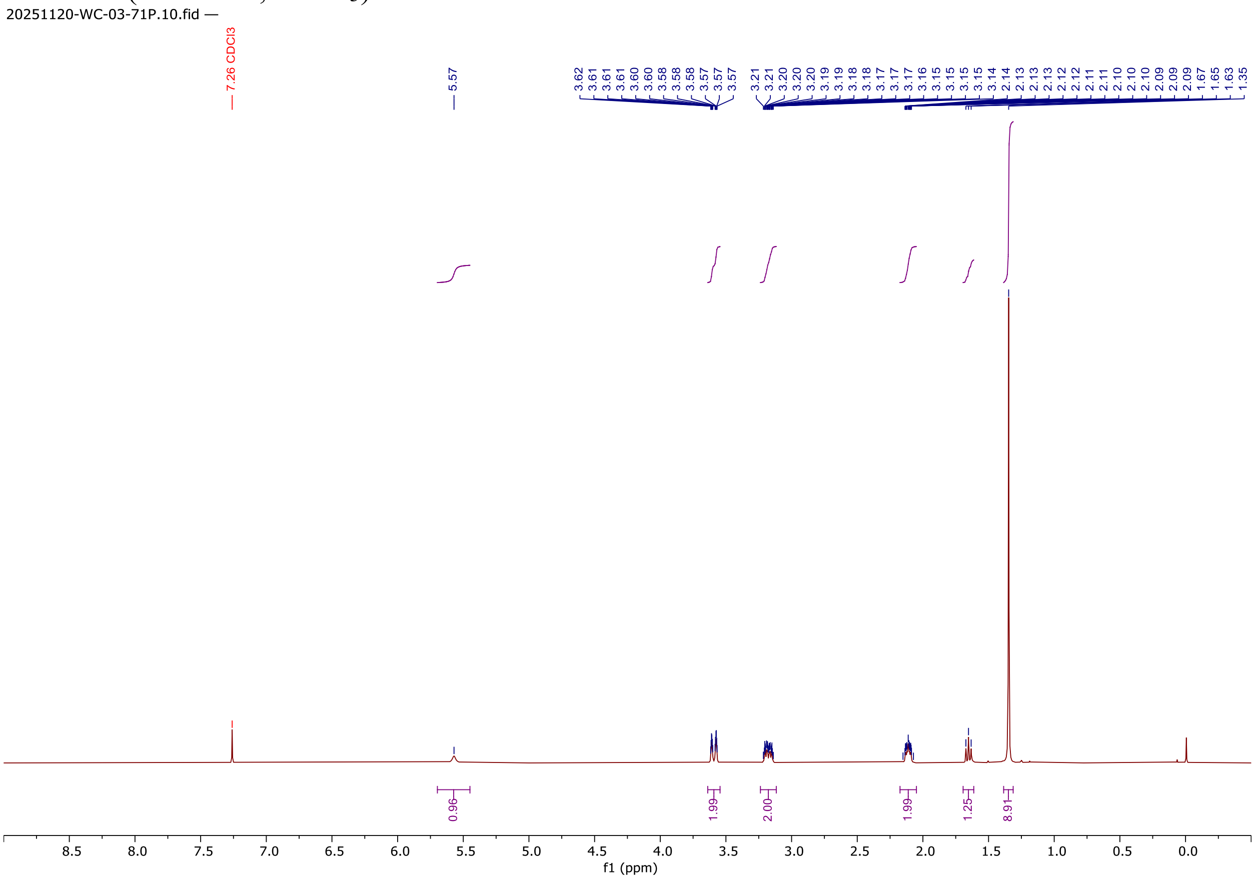

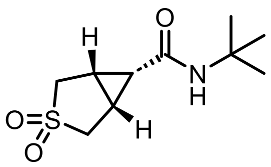

^{13}C NMR (101 MHz, $CDCl_3$)

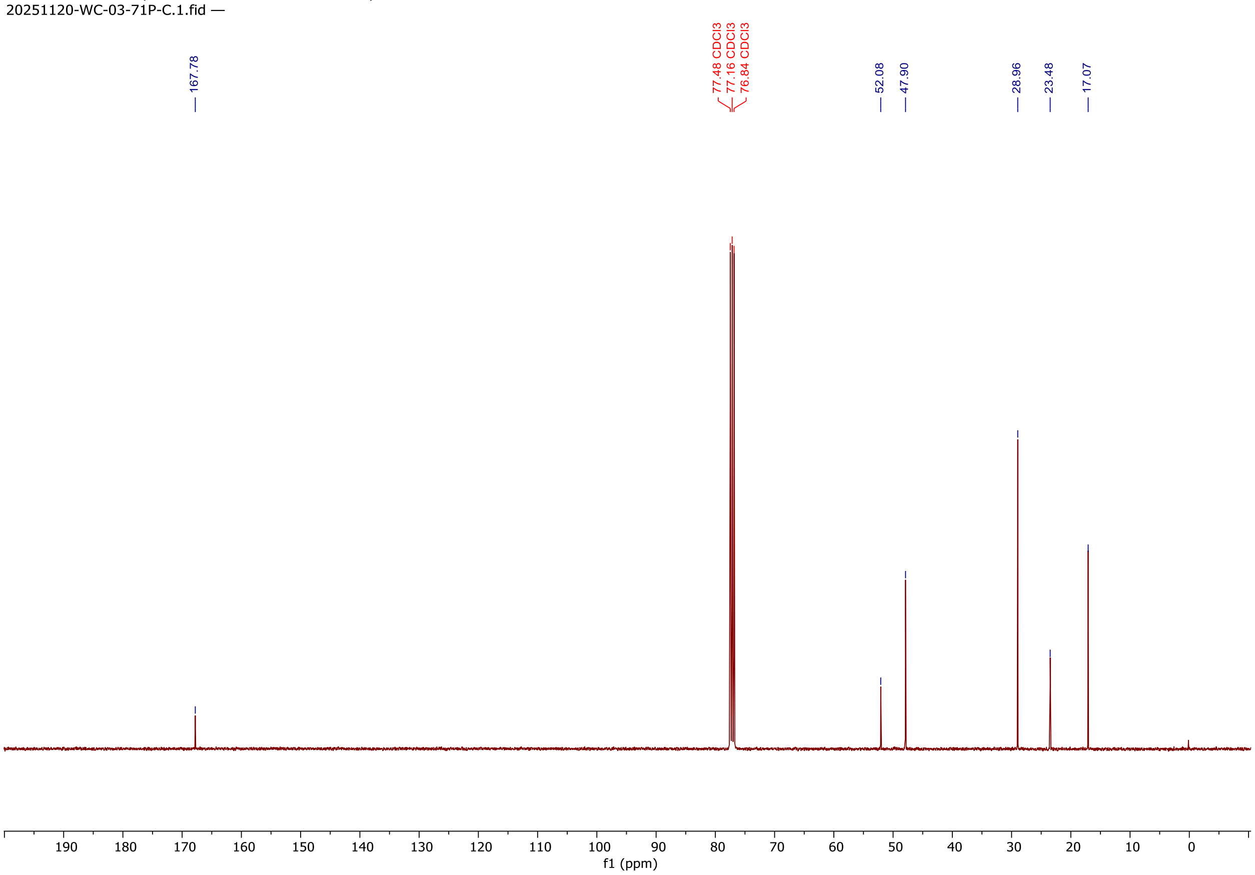

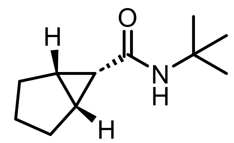

***endo*-N-(*tert*-Butyl)bicyclo[3.1.0]hexane-6-carboxamide (*endo*-23)**

^{1}H NMR (400 MHz, $CDCl_3$)

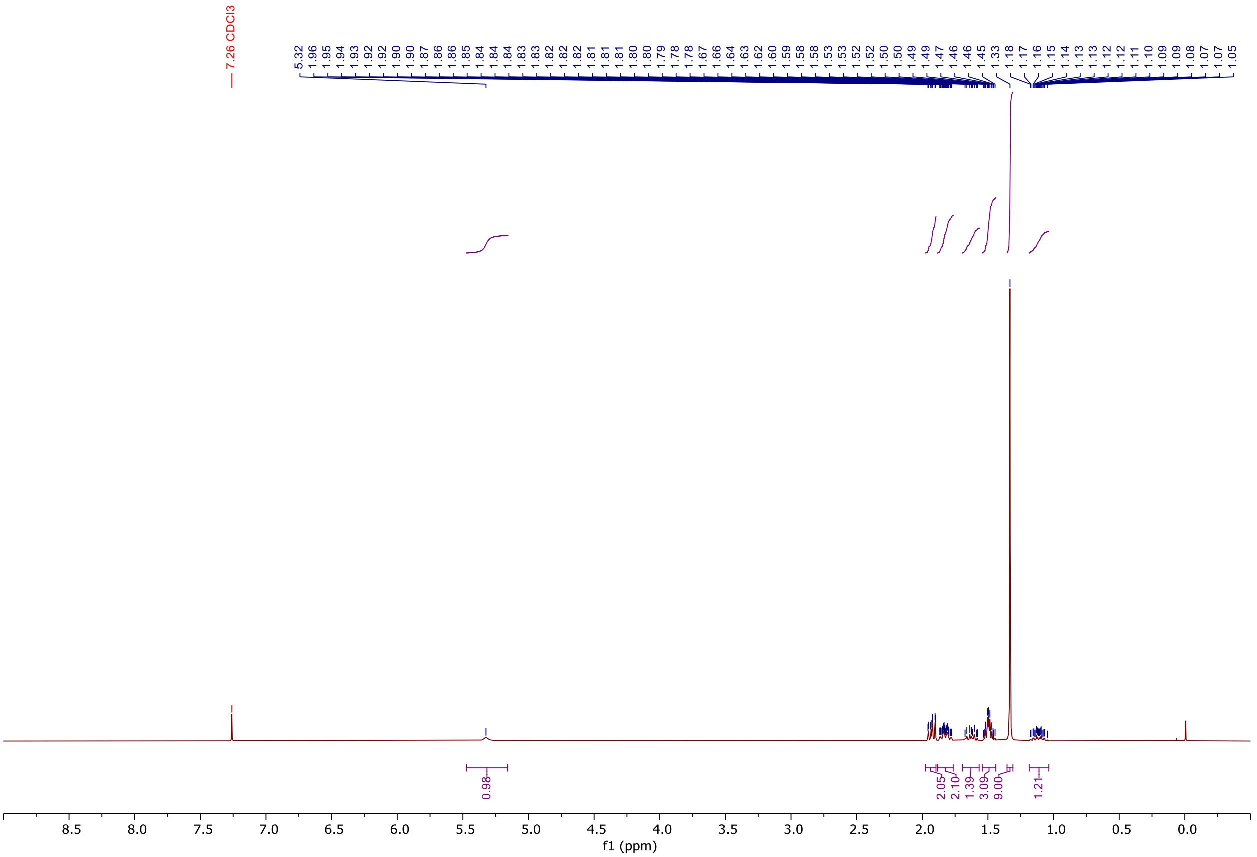

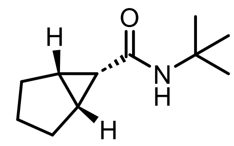

^{13}C NMR (101 MHz, $CDCl_3$)

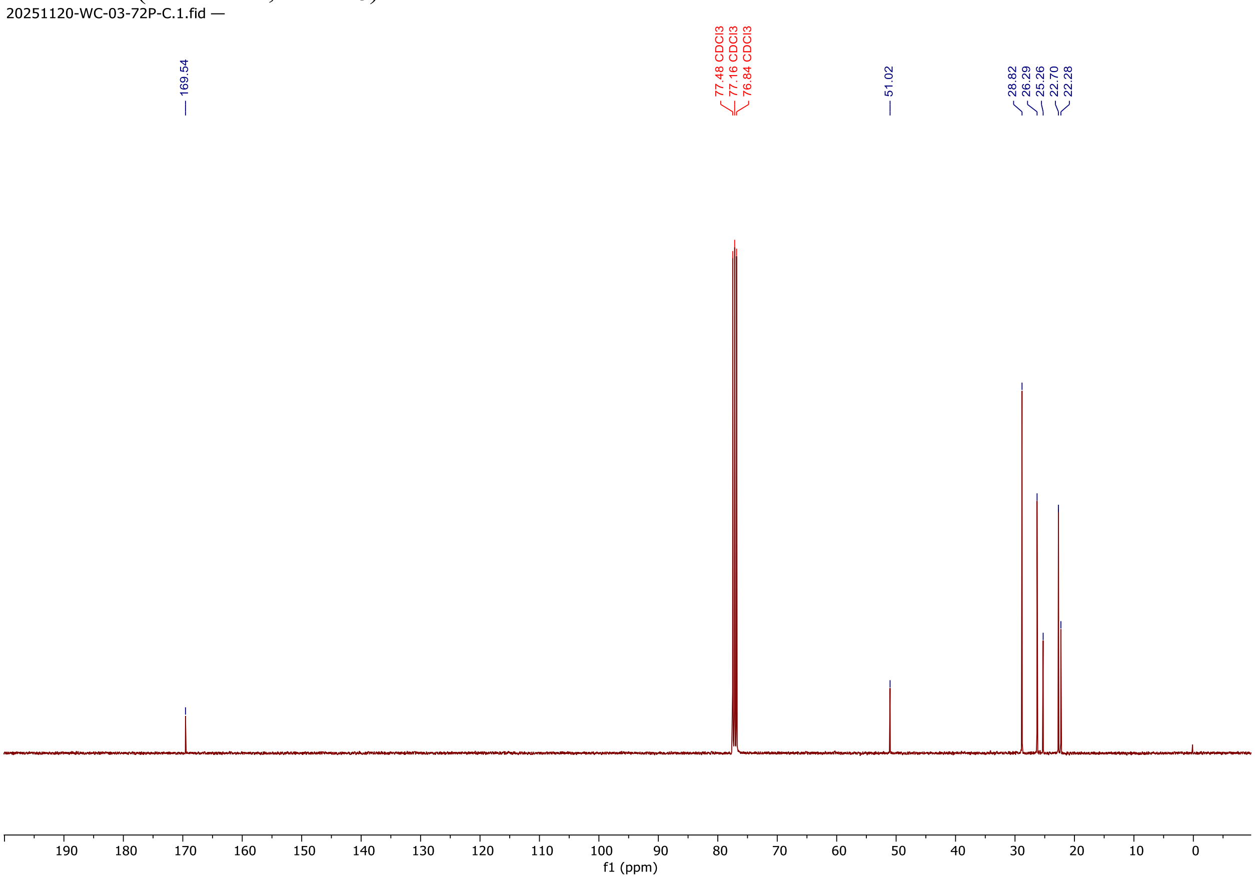

Supplement: Supplementary file 1 [file ol6c00392_si_001.pdf]
